# Progastrin Promotes Colorectal Cancer Stem Cell‐Like Properties via the Receptor PZR

**DOI:** 10.1002/advs.202502136

**Published:** 2025-08-03

**Authors:** Julie Nguyen, Marie Lafitte, Maud Barbery, Kevin Espie, Maya Jeitany, Romain Larive, Jihane Vitre, Conception Paul, Yvan Boublik, Elise Fourgous, Valérie Simon, Audrey Sirvent, Madeline Neiveyans, Steeve Thirard, Lucile Bansard, Morgan Maillard, Nathalie Coutry, Jacques Colinge, Philippe Jay, Pierre Martineau, Michael Hahne, Julie Pannequin, Serge Roche

**Affiliations:** ^1^ Equipe Labellisée LIGUE 2020 and FRM 2023 CRBM Univ Montpellier CNRS Montpellier 34293 France; ^2^ IGF Univ Montpellier CNRS Montpellier 23094 France; ^3^ IGMM Univ. Montpellier CNRS Montpellier 34293 France; ^4^ IRCM Univ Montpellier ICM INSERM Montpellier 34298 France

**Keywords:** cancer stem cells, colon cancer, mAb, receptor, signaling, targeted therapy, tyrosine kinase

## Abstract

The tumor microenvironment promotes cancer progression in part by supporting cancer stem cells (CSC). In colorectal cancer (CRC), progastrin (PG), an orphan growth factor secreted by tumor cells within the tumor and its microenvironment, maintains CSCs by unidentified mechanisms. Here, the orphan receptor Protein Zero‐Related protein (PZR) is identified as an essential component of PG activity and demonstrated its utility as a therapeutic target. PZR is essential for growth of PG‐expressing tumors, while genetic inactivation in mice of *Mpzl1*, which encodes PZR, disrupted chemically‐induced colon transformation. Mechanistically, PG binds cellular glycosylated and dimeric PZR and promotes SHP2/SRC/β‐catenin‐dependent CSC‐like signaling. Blocking PZR by monoclonal antibodies inhibited PG‐dependent expansion of tumoroids derived from murine intestinal tumors and patient‐derived CRC cell lines, while in mice, it reduced adenoma formation triggered by *Apc* loss in stem cells and disrupted the tumor‐initiating capacity of PG‐expressing CRC cells. High *GAST* (which encodes PG) and *MPZL1* transcript levels in primary colon cancer patients is predictive of worse prognosis. Collectively, these findings support the inhibition of PZR as a potential targeted treatment of PG‐expressing CRC.

## Graphical abstract

The tumor microenvironment supports colorectal cancer (CRC) progression by maintaining cancer stem cells (CSCs) through progastrin (PG). This study identifies Protein Zero‐Related protein (PZR) as an essential receptor for PG activity, promoting CSC‐like signaling via SHP2/SRC/β‐catenin. Monoclonal antibody‐mediated PZR inhibition suppresses CSC‐like properties in experimental CRC models, highlighting PZR as a promising therapeutic target in PG‐expressing CRC.

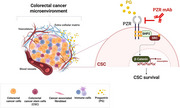

## 1 Introduction

Colorectal cancer (CRC) is a leading cause of malignancy‐related death worldwide. Currently, the clinical management of localized tumors involves surgery combined with adjuvant chemotherapy. However, tumor recurrence and metastatic spread occur in 40% of patients, resulting in a poor 5 year survival prognosis (<10%).^[^ ^1^ ^]^ Although targeted therapies and immune checkpoint inhibitors have been developed for CRC, they display only moderate clinical effects.^[^ ^2^, ^3^ ^]^ Therapeutic failure in CRC is often caused by intratumor heterogeneity, which is partially induced by cancer stem cells (CSCs) properties of resistant CRC cells.^[^ ^4^, ^5^ ^]^ This enables tumor relapse and/or metastatic spread. Deregulation of Wnt/β‐catenin signaling in CRC cells plays an essential role in this tumor process and is primarily caused by genetic alterations in signaling components.^[^ ^4^, ^5^ ^]^ Although this oncogenic pathway is of obvious therapeutic interest, developed inhibitors have not reached the clinic due to significant toxicity.^[^ ^6^ ^]^ Interestingly, this oncogenic pathway can be overactivated by signaling cues induced by tumor cells or their microenvironment.^[^ ^5^ ^]^ Understanding the signaling mechanisms that sustain Wnt/β‐catenin signaling and CSC‐like activity may be of therapeutic interest in CRC.

Tyrosine kinases (TKs) phosphorylate proteins on tyrosine residues to activate intracellular signaling^[^ ^7^ ^]^ and coordinate intestinal epithelial cell communication and fate decisions.^[^ ^8^ ^]^ Dysregulation of these activities promotes CRC development.^[^ ^9^ ^]^ Notably, the SRC oncogene, an essential membrane‐anchored signaling TK,^[^ ^10^ ^]^ drives intestinal stem/progenitor cell proliferation, tissue regeneration, and tumor formation in Drosophila and mouse models.^[^ ^11^ ^]^ Aberrant SRC expression and activity is observed in 50% of CRC patients and is associated with poor clinical prognosis, therapeutic resistance, and metastasis.^[^ ^12^ ^]^ This tumor activity is linked to CSC and epithelial‐to‐mesenchymal transition (EMT) traits, in particular though increasing Wnt/β‐catenin activity in these CRC cells.^[^ ^13^ ^]^ However, the mechanisms responsible for SRC oncogenic induction remain elusive, notably because SRC is rarely activated by somatic mutations in human cancer, and upstream receptors activating SRC signaling in CSC cells are not fully characterized.^[^ ^12^, ^14^ ^]^ Consistently, SRC inhibitors that were developed for the clinic have failed in CRC due to ineffective signaling inhibition and inadequate patient selection.^[^ ^15^ ^]^

SRC overactivation implicates the inhibition of control mechanisms,^[^ ^16^, ^17^, ^18^ ^]^ including inactivation of its regulatory tyrosine (i.e. Y530) by an SHP2‐dependent pathway, which impacts β‐catenin signaling.^[^ ^19^ ^]^ Furthermore, SRC‐dependent phospho‐proteomics in CRC revealed a forward loop mechanism in which SRC phosphorylates upstream receptors to induce its oncogenic function.^[^ ^20^, ^21^ ^]^ Interestingly, the orphan receptor protein zero‐related (PZR) was reported to interact with SHP2 and an SRC substrate^[^ ^22^, ^23^ ^]^. It can promote the EMT and CSC traits of various epithelial cancer cells,^[^ ^24^, ^25^, ^26^, ^27^, ^28^ ^]^ suggesting that PZR could serve as an activator of the oncogenic function of SRC in CRC. PZR is a glycosylated single‐pass transmembrane protein with an extracellular immunoglobulin domain and a short intracellular domain containing Y241 and Y263.^[^ ^22^ ^]^ When phosphorylated at these residues, it recruits and activates SHP2 via an SH2‐dependent mechanism.^[^ ^29^ ^]^ Phosphorylated PZR triggers SRC‐like signaling through an SHP2‐dependent mechanism, promoting epithelial cell migration in vivo.^[^ ^24^ ^]^ PZR is involved in SHP2‐dependent pathologies, including Noonan and LEOPARD autosomal dominant syndromes caused by SHP2‐activating mutations.^[^ ^24^, ^30^ ^]^ The *MPZL1* gene encoding PZR was identified as a marker of poor prognosis in various human epithelial cancers, notably in hepatocellular carcinoma (HCC), where PZR overexpression promoted SRC‐dependent tumor formation in a nude mouse model.^[^ ^26^ ^]^ However, it is unclear how aberrant *MPZL1* expression impacts tumor development, particularly the activation of SRC‐like signaling, which enables the β‐catenin oncogenic pathway. Notably, there is no identified extracellular cue expressed in human cancer that binds and activates PZR tyrosine phosphorylation. Interestingly, the plant glycoprotein concanavalin‐A, which is known to bind to N‐glycosylated proteins, displays binding affinity for PZR and induces receptor signaling, implicating a receptor dimerization and an SRC‐dependent mechanism.^[^ ^23^ ^]^

The hormone precursor of gastrin is a peptide secreted in the CRC microenvironment and a growth factor that promotes tumor development and therapeutic resistance.^[^ ^31^ ^]^ Progastrin (AA 21–101) is produced by aberrant GAST expression in CRC cells induced by oncogenic KRAS and β‐catenin/Tcf‐4^[^ ^32^, ^33^, ^34^, ^35^ ^]^ and is observed in more than 60% of CRC.^[^ ^31^ ^]^ Due to the lack of maturation enzymes in the colon (i.e., prohormone convertases and carboxypeptidase E), GAST upregulation results in the secretion of unprocessed hormone, which accumulates in the tumor and its microenvironment. PG promotes CRC formation in mice implicating SRC‐ and β‐catenin‐dependent pathways.^[^ ^36^, ^37^, ^38^, ^39^, ^40^ ^]^ For example, transgenic mice overexpressing PG in the intestine develop more preneoplastic lesions and carcinogen‐induced adenocarcinoma.^[^ ^41^ ^]^ Conversely, the inactivation of PG in human CRC cells impairs tumor formation, implicating the inhibition of Wnt‐ and Notch‐dependent signaling.^[^ ^39^ ^]^ Mechanistically, the Wnt/β‐catenin target and Notch ligand Jagged‐1 (JAG1) mediates the activation of Notch signaling by PG.^[^ ^42^ ^]^ PG induces angiogenesis and is a critical factor for CSCs in CRC.^[^ ^43^, ^44^ ^]^ During cytotoxic and hypoxic stress, PG tumor secretion increases significantly, which enables CSC survival and tumor progression.^[^ ^40^ ^]^ Consistent with these findings, antibody‐mediated PG inhibition reduced chemotherapeutic resistance and liver metastasis in experimental CRC.^[^ ^40^ ^]^ In addition, studies have identified the plasma level of PG as a biomarker for tumor development, therapeutic resistance, and metastasis^[^ ^40^, ^45^ ^]^ in CRC and other cancers.^[^ ^46^ ^]^

Although the malignant function of PG is well established, the cognate receptor mediating these pathological effects remains unclear. Notably, the CCKBR receptor for the mature amidated Gastrin peptides G‐34 (57‐94‐NH2) and G‐17 (76‐94‐NH2) contributes to PG CRC function,^[^ ^38^ ^]^ but this receptor displays negligible ligand affinity.^[^ ^47^ ^]^ GPR56^[^ ^48^ ^]^ and Annexin II^[^ ^47^, ^49^ ^]^ have been proposed as PG receptors, but validated cell surface receptors in human CRC cells remain elusive. Therefore, identifying such receptors could lead to the discovery of an attractive target in CRC.

In the present study, we demonstrated that the glycosylated and dimeric forms of PZR mediated PG binding to CRC cells. PG induced PZR phosphorylation at Y241 and Y263, enabling SHP2 activation and SRC/β‐catenin‐dependent CSC signaling. PZR expression mediated the tumor development of PG‐high CRC cells in nude mice, while murine *Mpzl1* deletion reduced AOM/DSS‐induced colon transformation. Antibody‐mediated PZR inhibition successfully reduced adenoma formation upon loss of *Apc* in mouse intestinal stem cells and the tumor‐initiating capacity of PG‐high CRC cells in immune‐deficient mice. In agreement, high *GAST* and *MPZL1* transcript levels predicted worse prognosis in primary colon cancer patients.

## 2 Results

### 2.1 PZR Mediates PG Binding to CRC Cells

**Figure 1:**
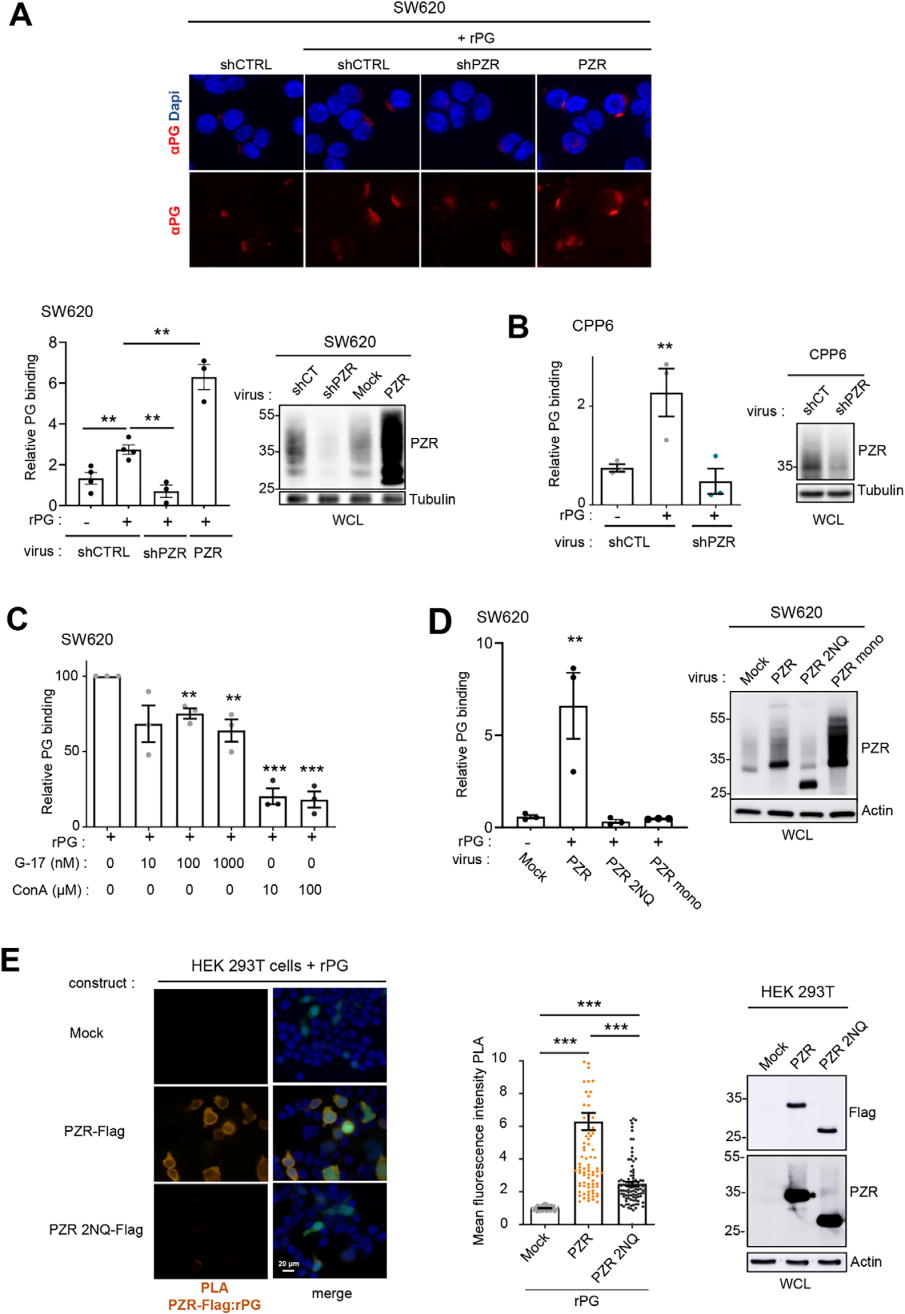
PZR mediates PG binding to CRC cells. A,B) PZR‐dependent PG binding to CRC cells. A representative example of rPG (10 nm) cellular binding at 4 °C (top) and its quantification (relative fluorescence intensity per cell; bottom) from SW620 cells (A) and CPP6 (B) cells transduced with indicated PZR constructs as shown (mean ± SEM, *n* = 3–4). WB analysis of PZR levels in SW620 and CPP6 CRC cells transduced with the indicated construct. C) rPG cell binding competition by G‐17 and Con‐A. Shown is the quantification of rPG binding to SW620 cells (% maximum binding) in the presence of indicated concentrations of G‐17 and Concanavalin‐A (mean ± SEM, n = 3). D) Contribution of PZR dimerization and N‐glycosylation to rPG cellular binding. Relative rPG binding to SW620 cells expressing indicated PZR mutants (mean ± SEM, *n* = 3). WB analysis of WT and mutants PZR in SW620 cells. E) PLA analysis of rPG binding on HEK293T cells transfected with PZR‐FLAG WT or 2NQ mutant constructs. Transfected cells were detected by GFP  co‐expression. Left: representative example; middle: PLA quantification (mean fluorescence intensity) (mean ± SEM of 150 cells analyzed from three independent experiments); right: WB analysis of indicated PZR‐FLAG levels. ^**^ *p* < 0.01; ^***^ *p* < 0.001; Student's *t* test. MW (kDa) are indicated in WBs.

To identify a PG cell surface receptor that mediates SRC oncogenic signaling in CRC cells, we searched for an orphan receptor and an SRC substrate that in turn activates SRC kinase activity. Taking advantage of our previous SRC‐dependent phospho‐proteomic analyses in PG‐expressing SW620 CRC cells (PG‐high CRC cells), ^[^ ^50^ ^]^ we identified PZR as an attractive candidate. We first confirmed that SRC regulates PZR phosphorylation at Y241 and Y263 in these CRC cells (Figure S1A,B, Supporting Information). Next, we investigated the contribution of PZR expression to PG binding in CRC cells. The cells were incubated at 4 °C to allow the accumulation of TK signaling receptors on the cell surface, followed by incubation with or without recombinant PG (rPG). After extensive washing, bound PG was quantified by indirect immunofluorescence using a specific anti‐PG antibody, as described^[^ ^51^ ^]^ (**Figure** **1**A). We first detected specific rPG binding, which was enhanced in PZR‐overexpressing cells and reduced upon *MPZL1* silencing, indicating that PZR expression mediates PG cellular binding (Figure 1A). This was confirmed by similar results obtained in the patient‐derived PG‐high CRC line CPP6^[^ ^44^, ^52^ ^]^ (Figure 1B). A low signal was also detected in the absence of rPG, which was attributed to endogenous PG, as it was reduced by shRNA‐mediated PG cell depletion (Figure S2A,B, Supporting Information). These findings revealed the dependence of PG binding on PZR expression in CRC cells.

Next, we determined the PZR properties involved in this molecular process. First, we investigated whether PZR binds to other gastrin peptides. A 100‐fold excess of the mature peptidic hormone G‐17 (1 µm) resulted in at most a 35% reduction in cellular PG binding, which is inconsistent with a shared PG binding site mediated by PZR expression in CRC cells (Figure 1C). PZR was found to be highly glycosylated in CRC cells, as evidenced by 35–55 KDa entities detected by anti‐PZR WB, which were attenuated by N‐glycanase treatment (Figure 1A; Figure S3B, Supporting Information). Interestingly, Concanavalin‐A, at a concentration range that binds N‐glycosylated and dimeric PZR,^[^ ^53^ ^]^ strongly reduced PG binding, suggesting a contribution of PZR N‐glycosylation and/or dimerization in rPG binding (Figure 1C). Consistent with these findings, mutation of the predicted PZR N‐glycosylation sites (N50Q/N130Q mutations, PZR 2NQ)^[^ ^23^ ^]^ or the dimeric interface (PZR S86G/V145G/Q146K/P147T/G148S, PZR mono)^[^ ^53^ ^]^ abolished PG binding on PZR overexpressed SW620 cells (Figure 1D; Figure S3, Supporting Information). Finally, the PZR‐dependent PG cell binding was validated by a proximity ligation assay (PLA). PZR‐PG cell interactions were readily detected by PLA upon PZR expression in HEKT293 cells, unlike PZR 2NQ (Figure 1E). Overall, these results demonstrate the contribution of PZR N‐glycosylation and dimerization to PG cellular binding.

### 2.2 PG Induces PZR‐SHP2 Phospho‐Signaling in CRC Cells

**Figure 2:**
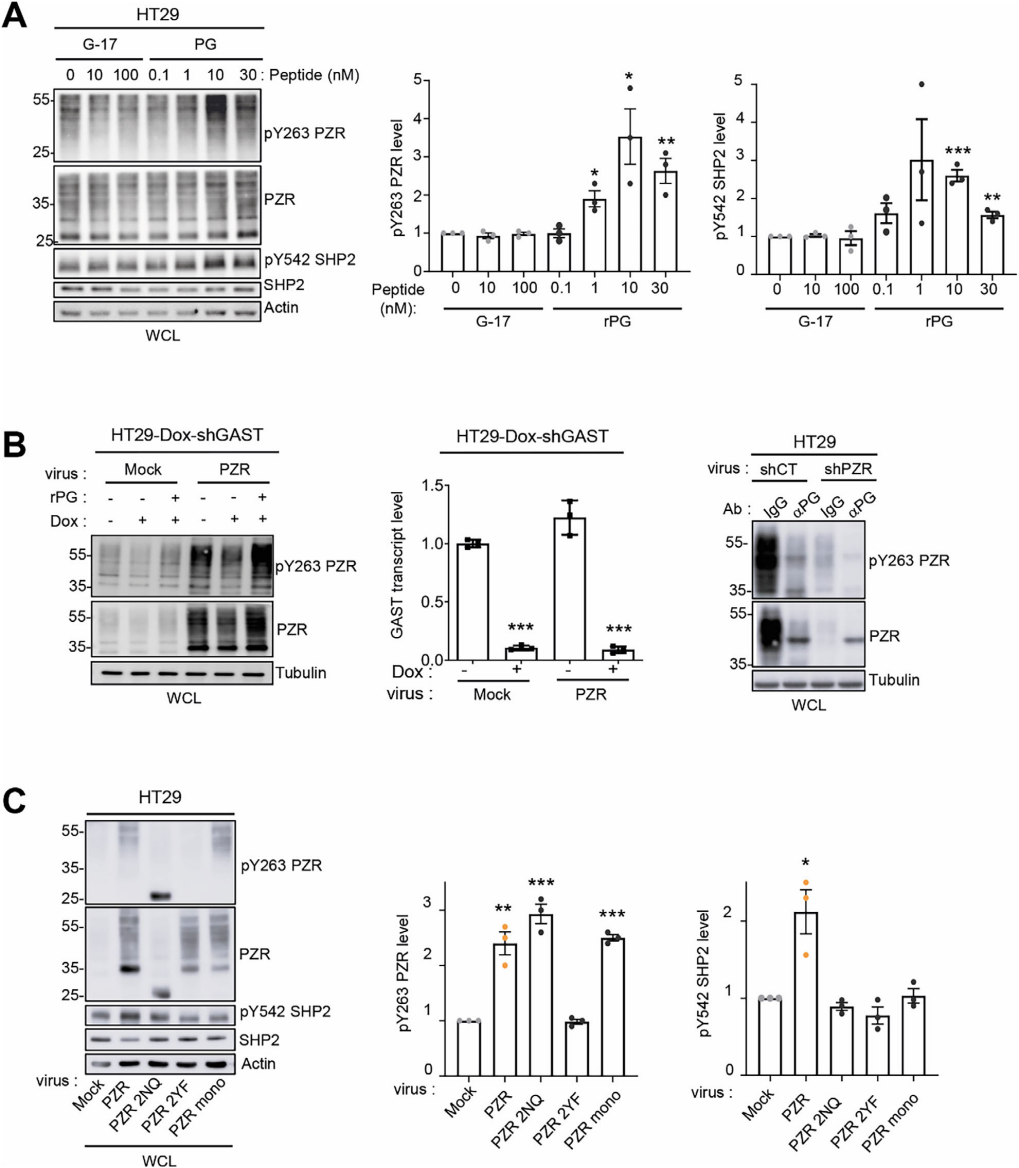
PG induces PZR‐SHP2 phospho‐signaling in CRC cells. A) Dose‐response effect of PZR‐SHP2 phospho‐signaling induced by rPG in HT29 cells. WB analysis of pY263 PZR and pY542 SHP2 levels in HT29 cells stimulated for 5 min with indicated concentrations of PG or G‐17. Left: a representative example; right: relative quantification (mean ± SEM, *n* = 3). B) PG‐dependent PZR phospho‐signaling in HT29 cells. Left: WB analysis of pY263 PZR levels in HT29 cells expressing doxycycline (Dox)‐induced shGAST (pTRIPZshGAST) and PZR constructs, as indicated. Cells were treated with Dox (1 µm) (or vehicle as a control) for 24 h before cell‐lysis. PG specific effects was evaluated by stimulated GAST‐silenced cells with rPG (10 nm) for 5 min as indicated. Middle: relative GAST transcript level assessed by qPCR (mean ± SEM, *n* = 3). Right: pY263 PZR levels in HT29 cells treated with an anti‐PG (or control IgG; 50 µg mL^−1^) for 24 h. WB PZR signal specificity was evaluated by PZR depletion (shPZR). Is shown a representative example of three independent experiments. C) Structure‐phospho‐signaling activity of PZR in HT29 cells. WB analysis of pY263 PZR and pY542 SHP2 levels in HT29 cells expressing indicated PZR mutants. Left: a representative example; right: relative quantification (fold control) expressed as the mean ± SEM, *n* = 3; ^*^ *p* < 0.05^; **^ *p* < 0.01; ^***^ *p* < 0.001; Student's *t* test. MW (kDa) are indicated in WBs.

We next investigated the effect of PG on PZR phospho‐signaling by focusing on human HT29 CRC cells, which express a lower level of endogenous PG.^[^ ^44^ ^]^ rPG (10 nm) stimulation induced a rapid and large increase in cellular PZR phosphorylation at Y263 and Y241, which was detected after 1 min and peaked after 5 min of stimulation (Figure S4A, Supporting Information). A dose‐response experiment revealed an EC50 of 2–3 nm with a maximal effect at 10 nm (**Figure** **2**A). This molecular response was not observed with 10–100 nm G‐17, consistent with the specific PG‐PZR signaling pathway (Figure 2A). rPG induced a similar effect on the activation of SHP2 (as measured by phosphorylation at Y542, pSHP2), the main effector of PZR (EC50 of ≈2–3 nm with a maximal effect at 10 nm, no effect of G‐17) (Figure 2A). siRNA‐mediated PZR inhibition reduced SHP2 activation, validating the implication of PZR in this PG signaling response (Figure S4B, Supporting Information). Conversely, inhibition of endogenous PG reduced PZR phosphorylation at Y263, an effect that was reversed by rPG (Figure 2B). Finally, we performed a structure‒function analysis to determine the mechanism by which PZR induces SHP2 phosphorylation in these CRC cells (Figure 2C). PZR overexpression led to an increase in pY263‐PZR and pY542‐SHP2 levels, which was abolished by the mutation of Y263 and Y241 to F (PZR 2YF mutant). Mutation of the glycosylation site (PZR 2NQ) or the dimeric interface (PZR mono) affecting PG binding to PZR also inhibited SHP2 activation (Figure 2C). Collectively, these data support the notion that PG activates PZR‐SHP2 signaling in CRC cells.

### 2.3 PZR Depletion in Mice Reduces Colon Transformation

To investigate the role of PZR during colorectal cancer (CRC) development, we first generated a PZR‐deficient mouse model by inserting a *LacZ* cassette into the *Mpzl1* gene, resulting in depletion of PZR expression. The successful inactivation of *Mpzl1* in the colon was confirmed by reduced transcript levels and increased colonic β‐galactosidase activity (Figure S5A,B, Supporting Information). PZR‐deficient mice did not exhibit any overt phenotype in body weight or colonic morphology, suggesting that PZR is dispensable for normal colonic homeostasis (Figure S5C–E, Supporting Information). In agreement with this, organoids derived from colonic crypts of wild‐type (WT) and *Mpzl1*‐deficient mice showed no differences in baseline growth (Figure S6A, Supporting Information). However, treatment with recombinant PG (rPG) significantly promoted organoid expansion in wild‐type (WT) but not in PZR knockout (KO) organoids, (Figure S6A, Supporting Information) indicating that PZR is functionally required for PG‐induced stem cell‐like properties in the colon. Although previous studies have implicated the gastrin receptor CCKBR and the inhibition of transforming growth factor−β (TGF‐β) superfamily members, such as Bone Morphogenetic Protein (BMP), in the mitogenic activity of PG,^[^ ^38^, ^51^ ^]^ the CCKBR antagonist L365,260^[^ ^54^, ^55^ ^]^ did not affect PG‐induced organoid expansion (Figure S6B, Supporting Information). Likewise, withdrawal of the BMP antagonist Noggin from the medium had no impact on PG‐mediated mitogenic effects in either genotype (Figure S6C, Supporting Information). These results collectively support a PG–PZR‐dependent mitogenic function in the colon that is independent of CCKBR and BMP signaling pathways.

**Figure 3:**
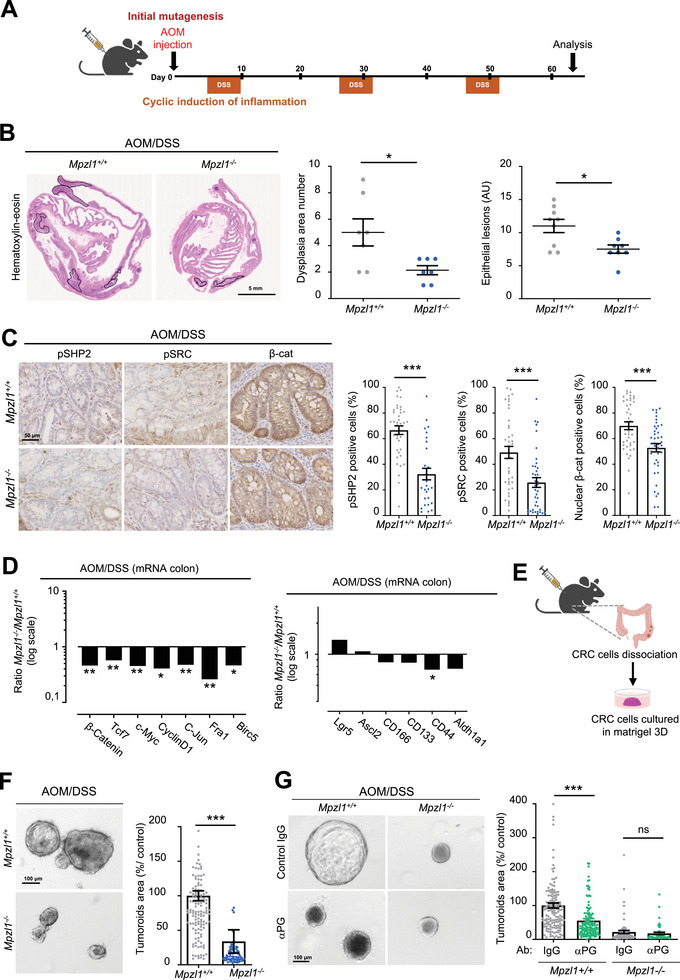
PZR gene inactivation reduces murine colonic transformation. A) Workflow of colonic transformation by mice AOM/DSS treatment. B) PZR gene (*Mpzl1*) inactivation reduces colonic transformation. Left: a representative example of colonic transformation following AOM/DSS treatment stained with HE, where transformed areas are highlighted. Right: quantification of dysplasia area and epithelial lesion between *Mpzl1+/+* and *Mpzl1‐/‐* mice (mean ± SEM, *n* = 7–9 mice per group; ^*^ *p* < 0.05 Mann–Whitney test). C) pSHP2/pSRC/β‐catenin signaling is reduced in transformed colonic epithelium of *Mpzl1*‐/‐ mice. IHC analysis of pSHP2, pSRC, and active β‐catenin level (% of positive cells) from AOM/DSS‐treated transformed colon of *Mpzl1+/+* and *Mpzl1‐/‐* mice. Left: a representative example; right: quantification as the mean ± SEM of 10 area/mice, *n* = 4–5 mice per group; ^***^ *p* < 0.001 Mann–Whitney test). D) Expression of selected β‐catenin‐dependent transforming genes is reduced in the proximal colon of AOM/DSS‐treated *Mpzl1*‐/‐ mice. Is shown the transcript logarithmic ratio of indicated β‐catenin‐targeted (left) and CSC genes (right) between AOM/DSS‐treated colon of *Mpzl1+/+* and *Mpzl1‐/‐* mice (mean ± SEM, *n* = 4–6 mice per group; ns: *p* > 0.05; ^*^ *p* < 0.05; ^**^ *p* < 0.01; ^***^ *p* < 0.001; Student's *t* test). E) Workflow of colonic tumoroid development derived from AOM/DSS‐treated mice. F) PZR regulates colonic tumoroid development. A representative example and quantification of tumoroids area (% control) of isolated colonic epithelial cells derived from AOM/DSS‐treated *Mpzl1+/+* and *Mpzl1‐/‐* mice (mean ± SEM of >100 tumoroids analyzed per mouse, *n* = 3 mice per group; ns, *p* > 0.05; ^***^ *p* < 0.001; Mann–Whitney test). G) PG‐dependent PZR colonic tumoroid function. The contribution of secreted PG on tumoroid development was assessed by treating cells adding anti‐PG antibody or control IgG (50 µg/mL^−1^) in the culture medium. Is shown a representative example (left) and the quantification of tumoroids area (% control) from isolated colonic epithelial cells derived from AOM/DSS‐treated *Mpzl1+/+* and *Mpzl1‐/‐* mice (mean ± SEM of ≈100 tumoroids analyzed per mouse, *n* = 3 mice per group; ns, *p* > 0.05; ^***^ *p* < 0.001; Mann–Whitney test).

Next, mice were subjected to carcinogen‐induced colon transformation by injection of the mutagen azoxymethane (AOM) and subsequent addition of dextran sodium sulfate (DSS) to the drinking water (3 DSS treatments for 5 days) for 90 days (**Figure** **3**A; Figure S7A, Supporting Information), which induced erosion of the colonic mucosa, leading to chronic inflammation.^[^ ^56^ ^]^ This well‐established model of colon carcinogenesis mimics human colitis‐associated carcinogenesis (CAC).^[^ ^40^ ^]^ Interestingly, PG was involved in AOM‐induced murine colon carcinogenesis,^[^ ^41^, ^57^ ^]^ consistent with a PG‐dependent transforming effect in this model. We observed that the number of epithelial lesions, as well as the area of dysplasia, were reduced in the *Mpzl1*‐deficient mice (Figure 3B). A similar trend was observed for macroscopically detectable tumors, despite the low number of tumors observed in our setting (Figure S7B, Supporting Information). An increase in epithelial cell apoptosis was detected in the transformed colon area of *Mpzl1*‐deficient mice, while no clear difference in epithelial cell proliferation and immune cell infiltration was observed (Figure S7C,D, Supporting Information). Next, immunohistochemical (IHC) analysis revealed inhibition of active pSHP2, pY418 SRC (pSRC) and active nuclear β‐catenin levels in the transformed colon of *Mpzl1*‐deficient mice (Figure 3C), consistent with a reduction in the expression of established PG‐dependent and oncogenic β‐catenin target genes (i.e., *β‐catenin, Tcf7, c‐Myc, Cyclin D1, c‐Jun, Fra1* and *Birc5*) (Figure 3D). Conversely, an increase in *Mpzl1* promoter activity (as evaluated in β‐Gal‐positive cells) was noted in the epithelial lesions of *Mpzl1*‐deficient mice (Figure S7E, Supporting Information), suggesting that *Mpzl1* transcriptional upregulation occurs upon colonic transformation. We next used this CAC mouse model to evaluate the contribution of PZR to colonic CSC‐like properties. Among the CSC markers tested, the transcript level of the PG‐CSC‐like target gene *CD44* ^[^ ^51^ ^]^ (which encompasses CD44 and its isoforms) was significantly reduced in PZR‐deficient mice (Figure 3D). By generating tumoroids from the isolated colonic epithelium of AOM/DSS‐treated mice (Figure 3E), we found that PZR depletion strongly reduced tumoroid expansion (Figure 3F). The addition of a PG‐neutralizing antibody^[^ ^39^ ^]^ had a similar inhibitory effect, revealing a PG‐secreting autocrine loop involved in CSC‐like properties (Figure 3G). However, PG inactivation had no effect on PZR‐depleted cells (Figure 3G), revealing the PZR‐dependent CSC‐like function of PG in these transformed colonic epithelial cells.

### 2.4 PZR Mediates the Tumor Properties of PG‐Expressing CRC Cells

**Figure 4:**
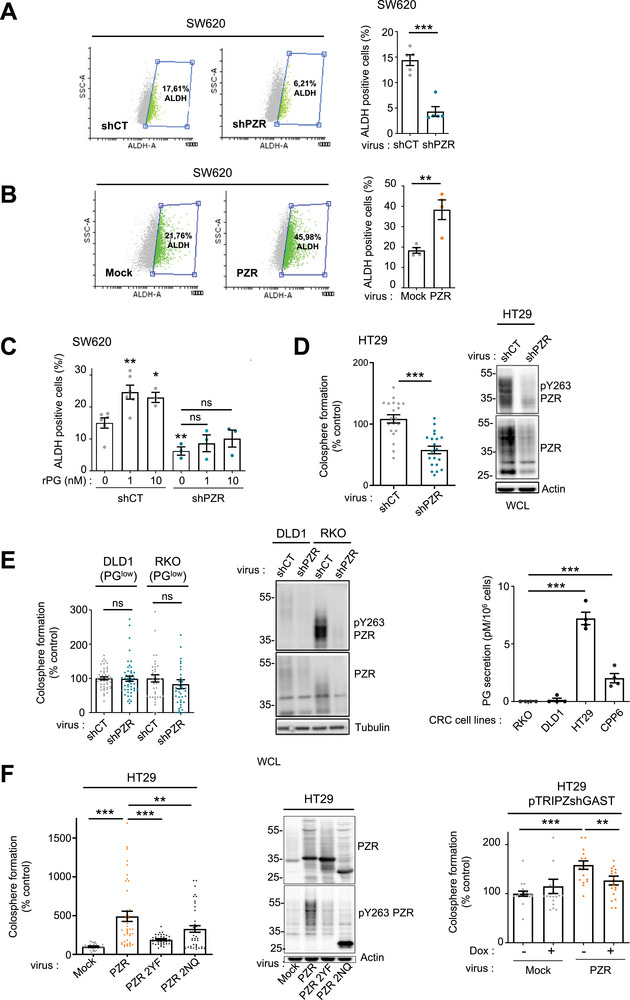
PZR mediates PG‐dependent CRC CSC‐like properties. A) PZR regulates the activity of the CSC marker ALDH in SW620 cells. B) Overexpression of PZR increases ALDH activity. Shown is a representative example of FACS analysis of ALDH‐positive cells (left) and its quantification. C) ALDH activity induced by rPG is dependent on PZR expression. Shown is the ALDH activity induced by increasing doses of rPG in cells infected with the indicated virus (shCT versus shPZR). (A–C) shows the mean ± SEM; *n* = 3‐5; ns: *p* > 0.05; ^*^ *p* < 0.05; ^**^ *p* < 0.01; ^***^ *p* < 0.001; Student's *t*‐test. D) PZR depletion reduces colonosphere formation of PG‐high (HT29) cells. Colonosphere formation (% control) of CRC cells infected with the indicated virus. E) PZR depletion does not reduce colonosphere formation of PG‐low (PG^low^) CRC cells (DLD1, RKO) Colonosphere formation (% control) of CRC cells infected with the indicated virus. Is shown the concentration of secreted PG of indicated CRC cell‐lines was measured by ELISA (mean ± SEM of 4 independent replicates; ns, *p* > 0.05; ^***^ *p* < 0.001; Mann–Whitney test). WB analysis of PZR expression. F) PZR expression increases colonosphere formation in HT29 cells, implying its dimerization and N‐glycosylation. Colonosphere formation (% control) of cells infected with the indicated virus. WB analysis of PZR expression. (D–F) shows the mean ± SEM of 5–10 replicates / condition, *n* = 3‐4 independent experiments; ns, *p* > 0.05; ^***^ *p* < 0.001; Mann–Whitney test.

The CSC‐like function of PZR was further investigated in human PG‐high CRC cell lines using established cancer stem cell (CSC) markers, including ALDH enzymatic activity and the cell surface expression of CD44 variant isoform v6 (CD44v6) and CD26.^[^ ^44^ ^]^ PZR knockdown significantly reduced ALDH activity in SW620 cells (**Figure** **4**A) and in HT29 cells (Figure S8A, Supporting Information), along with decreased surface expression of CD44v6 and CD26 in SW620 cells (Figure S8B, Supporting Information). Conversely, PZR overexpression in SW620 cells led to increased expression of these CSC markers (Figure 4B; S8C, Supporting Information). Stimulation with recombinant PG (rPG) also enhanced ALDH activity in a dose‐dependent manner, an effect that required PZR expression (Figure 4C). This response was abolished by pharmacological inhibition of SHP2 and SRC family kinases (Figure S8D, Supporting Information), supporting a role for SHP2/SRC signaling in PG‐mediated CSC‐like activity.

To further investigate the role of PZR in CSC properties, we evaluated colonosphere formation. PZR depletion impaired sphere formation in PG‐high HT29 cells (Figure 4D) but had no significant effect in PG‐low CRC cell lines DLD1 and RKO, which exhibited minimal PG secretion as determined by ELISA (Figure 4E). Conversely, PZR overexpression in HT29 cells led to an increased number of colonospheres, a function dependent on both endogenous PG expression and PZR post‐translational modifications, including tyrosine phosphorylation and N‐glycosylation (Figure 4F). This PZR‐mediated enhancement of colonosphere formation was markedly suppressed by inhibitors targeting SRC and SHP2, as well Wnt/β‐catenin and Notch signaling pathways (Figure S9A, Supporting Information), supporting the involvement of a PZR‐SRC‐SHP2‐β‐catenin–Notch axis in regulating CSC features of PG‐high CRC cells. Similar results were obtained in tumoroid models (Figure S9B,C, Supporting Information), further validating the role of this PZR pathway in promoting CSC‐like properties in PG‐high CRC cells. Consistent with these findings, both phosphorylated PZR (pPZR) and total PZR protein levels were elevated in SW620 cells cultured under sphere‐forming conditions, which enrich for CSCs, a response that was dependent on endogenous PG secretion (Figure S10A,B, Supporting Information).

**Figure 5:**
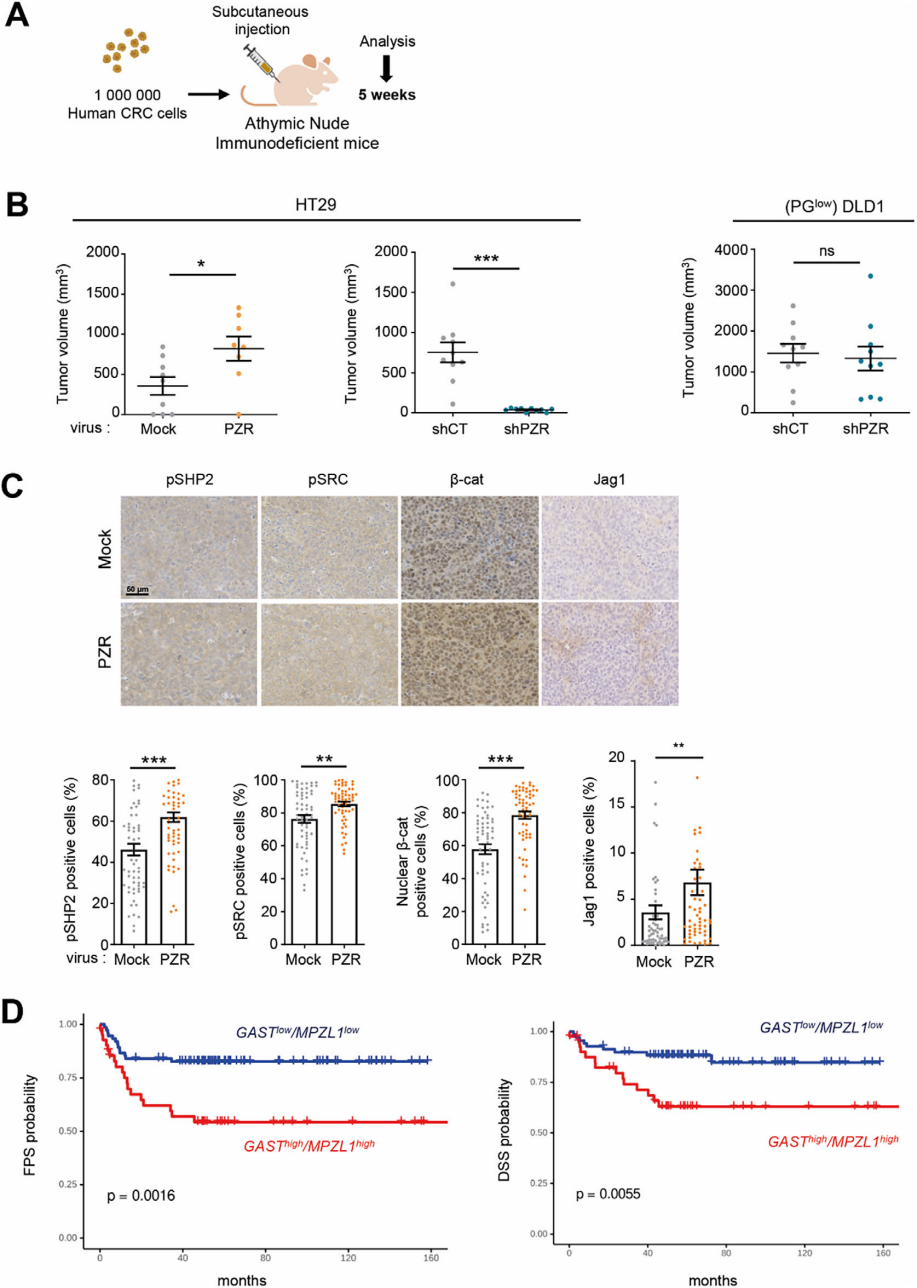
PZR pro‐tumor function in PG‐high CRC cells. A) Workflow of CRC formation in nude mice. B) PZR affects subcutaneous tumor growth of PG‐high HT29 cells, but not of PG‐low (PG^low)^ DLD1 cells. Cells infected with the indicated vectors were inoculated s.c. into nude mice. Graphs show the volume of the isolated tumors at day 30 post‐transplantation (means ± SEM; *n* = 8–10 mice/group; ns, *p* > 0.05; ^*^ *p* < 0.05; ^***^ *p* < 0.001; Student's *t* test). C) PZR overexpression enhances pSHP2/pSRC/β‐catenin signaling and JAG1 protein level in HT29 cells in vivo. IHC analysis of pSHP2, pSRC, active β‐catenin and JAG1 protein levels from subcutaneous tumors. A representative example (top) and the quantification (% of positive cells) from indicated tumors (mean ± SEM, 10 area/mouse, *n* = 6 mice per group; ^**^ *p* < 0.01, ^***^ *p* < 0.001 Mann–Whitney test). D) *GAST* and *MPZL1* expression are associated with shorter progression‐free survival (FPS) and disease‐specific survival (DSS) in a cohort of primary CRC patients^[^ ^59^ ^]^. Is shown the Kaplan‐Meyer of *GAST^low^/MPZL1^low^* (blue lines) versus *GAST^high^/MPZL1^high^* transcript levels (red lines).

The pro‐tumoral function of PZR was next evaluated in vivo using immunodeficient mouse xenograft models (**Figure** **5**A). PZR overexpression enhanced the tumorigenicity of HT29 cells (Figure 5B), which was accompanied by increased CRC cell proliferation (Figure S11A, Supporting Information). IHC analysis demonstrated elevated SHP2 and SRC activities, as indicated by increased pSHP2 and pSRC levels, along with enhanced nuclear β‐catenin and expression of its PG‐dependent gene target, the Notch signaling activator JAG1^[^ ^42^ ^]^ in CRC cells expressing PZR (Figure 5C; Figure S11B, Supporting Information). Furthermore, elevated levels of the angiogenic factors CD131 and VEGFA were observed in PZR‐expressing tumors (Figure S11C, Supporting Information), consistent with the reported pro‐angiogenic roles of PZR and PG in CRC.^[^ ^43^, ^58^ ^]^ Conversely, PZR knockdown strongly impaired tumor formation in HT29 cells, whereas DLD1 cells, which express low PG levels, were minimally affected (Figure 5B). Finally, analysis of a primary colon cancer patient cohort^[^ ^59^ ^]^, revealed that high *GAST* and *MPZL1* transcript levels were associated with shorter progression‐free survival and disease‐specific survival (Figure 5D; Figure S11D, Supporting Information). In contrast, *CCKBR* expression was barely detectable in this CRC cohort, suggesting a limited role for this receptor in mediating PG‐driven tumorigenic effects (Figure S12A, Supporting Information). Accordingly, pharmacological inhibition of CCKBR did not impact on colonosphere or tumoroid formation of PG‐high CRC cell lines HT29 and SW620 (Figure S12B,C, Supporting Information). Collectively, these findings support a pro‐tumoral function of PZR in PG‐high CRC cells.

### 2.5 Antibody‐Mediated PZR Inhibition Reduces Tumoroid Growth

**Figure 6:**
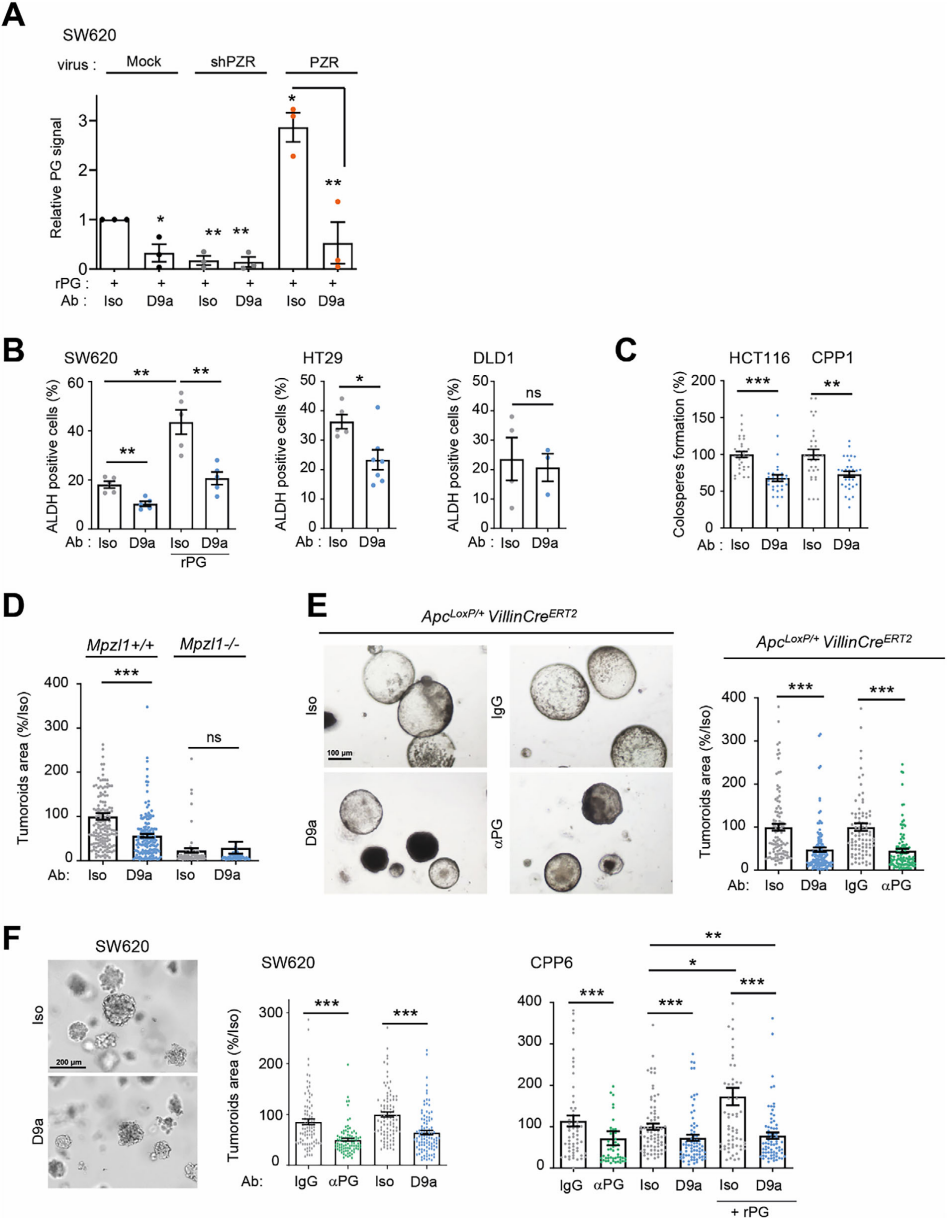
Antibody‐mediated PZR inhibition reduces tumoroids derived from murine intestinal tumors and CRC patients. A) PZR mAb D9a reduces rPG binding to SW620 cells. Cells were pretreated overnight with D9a (10 µg mL^−1^) or Iso as a control before performing rPG cell binding using the protocol and cell lines described in Figure 1. Is shown the quantification (relative fluorescence intensity per cell expressed as mean ± SEM, *n* = 3; ^*^ *p* < 0.05; ^**^ *p* < 0.01; ^***^ *p* < 0.001; Student's *t*‐test) (right). B) The PZR mAb D9a reduces PG‐dependent ALDH activity in CRC cells. FACS analysis of ALDH activity of PG‐high (SW620, HT29) and PG‐low (DLD1) CRC cells treated with the indicated mAb (10 µg mL^−1^) for 24 h. The PG‐dependent effect was confirmed in SW620 stimulated with 1nm (rPG). ALDH activity (% positive cells) is expressed as the mean ± SEM; *n* = 3; ns: *p* > 0.05; ^*^ *p* < 0.05; ^**^ *p* < 0.01; Student's *t* test. C) PZR mAb D9a reduces colonosphere formation in PG‐high CRC cells. HCT116 and CPP1 cells were treated with the indicated mAb (10 µg mL^−1^) twice weekly. Colonosphere formation (% control) is expressed as mean ± SEM 10 replicates/condition; *n* = 3; ns: *p* > 0.05; *p < 0.05; ^**^ *p* < 0.01; Man‐Witney test. D) PZR mAb D9a reduces tumoroids derived from colonic epithelial cells of AOM/DSS‐treated mice. Quantification of tumoroid area (% control) of isolated colonic epithelial cells treated with indicated mAb (10 µg mL^−1^). E) PZR mAb D9a reduces tumoroids derived from intestinal epithelial cells of tamoxifen‐treated *Apc^LoxP/+^*‐*Villin‐Cre^ERT2^* mice for 30 d. A representative example (left) and quantification (right) of tumoroid area (% control) of isolated intestinal epithelial cells treated with indicated antibodies (mAb: 10 µg mL^−1^, IgG control, and anti‐PG: 50 µg mL^−1^). (D,E show the mean ± SEM of >20 tumoroids analyzed from *n* = 3 mice per group; ns, *p* > 0.05; ^***^ *p* < 0.001; Mann–Whitney test). F) PZR mAb D9a reduces tumoroids derived from PG‐high CRC cells. A representative example (left) and quantification (right) of tumoroid area (% control) from SW620 and CPP6 CRC cells treated with indicated antibodies (mAb: 10 µg mL^−1^, IgG control, and anti‐PG: 50 µg mL^−1^) and 10 nm rPG where indicated. D and E show the mean ± SEM of >10 tumoroids analyzed from *n* = 3 mice per group; ns, *p* > 0.05; ^***^ *p* < 0.001; Mann–Whitney test).

To investigate whether PZR could be a therapeutic target in CRC, we developed a PZR‐blocking monoclonal antibody (mAb) by phage display screening against the extracellular domain of human PZR (PZR‐EXT). D9a was selected based on its ability to reduce cellular pY263 PZR levels and colonosphere formation of HT29 CRC cells (Figure S13A,B, Supporting Information). PZR specificity was verified by ELISA, PLA of HEK293T cells overexpressing PZR‐FLAG, and FACS analysis of epithelial cancer cells (Figure S13C–F, Supporting Information). In contrast to the control isotype (Iso), D9a inhibited PZR‐dependent rPG binding to CRC cells (**Figure** **6**A; Figure S14A,B, Supporting Information) and CSC‐like properties in vitro, particularly ADLH activity in PG‐high CRC cells and rPG‐stimulated CRC cells (Figure 6B), and formation of colonospheres derived from PG‐high CRC cells (Figure 6C).

The anti‐CSC‐like effect of D9a was next examined on tumoroids derived from murine intestinal cancer models (Figure 6D). D9a inhibited colon tumoroid expansion derived from AOM/DSS‐treated mice; however, no inhibitory effect was observed in *Mpzl1*‐deficient mice, showing D9a specificity. Similarly, D9a reduced the size of tumoroids derived from Wnt‐dependent intestinal tumors induced by monoallelic inactivation of the intestinal *Apc* gene in Apc^LoxP/+^ Villin‐Cre^ERT2^ mice^[^ ^60^ ^]^ (Figure 6E). Antibody‐mediated PG inactivation in the culture medium had a similar effect, consistent with a PG‐secreting autocrine loop involved in β‐catenin CSC‐like function (Figure 6E). D9a reduced the size of human tumoroids derived from SW620 cells and the patient‐derived PG‐high CRC line CPP6 (Figure 6F). Mechanistically, D9a increased cell apoptosis, inhibited cell proliferation (Figure S15A,B, Supporting Information), and downregulated the SHP2/SRC/β‐catenin signaling pathway (Figure S16A, Supporting Information), which are implicated in tumoroid development of SW620 cells (Figure S16B, Supporting Information). The PG‐dependent activity of D9a on these tumoroids was next examined. Antibody‐mediated PG inhibition produced similar inhibitory effects, demonstrating that PG affects tumoroids derived from these PG‐high CRC cells (Figure 6F). Unlike PG‐low CRC cell lines, D9a inhibited the tumoroid‐promoting effect of rPG on CPP6 cells as well as on tumoroids derived from additional patient‐derived PG‐high CRC cells (Figure 6F; Figure S17A,B, Supporting Information). Conversely, the observed D9a inhibitory effect obtained on CPP6 and SW620 cells were reversed by the addition of the recombinant soluble form of the PG effector JAG1 (rJAG1) to the medium, further validating the PG‐dependent D9a activity (Figure S18A,B, Supporting Information). Finally, D9a showed a similar inhibitory effect compared to that of the clinical EGFR mAb cetuximab in this set of experiments (Figure S17C, Supporting Information), confirming the therapeutic potential of a PZR mAb in PG‐expressing CRC cells.

### 2.6 D9a Targeting of PZR Reduces CSC‐Like Properties In vivo

**Figure 7:**
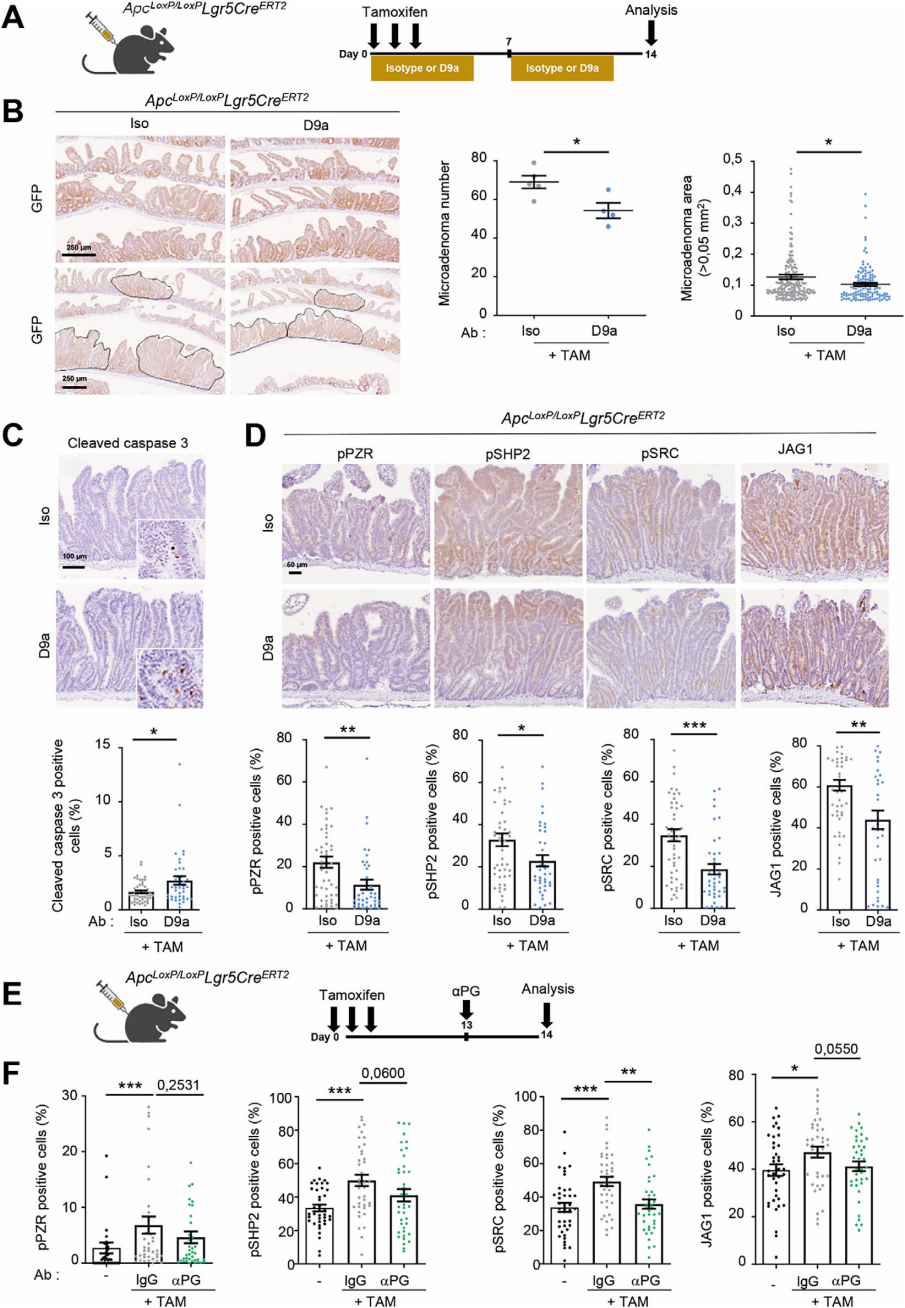
Antibody‐mediated PZR inhibition reduces Wnt‐dependent murine intestinal adenoma. A) Workflow to evaluate the contribution of PZR to adenoma formation induced by ISC transformation in mice. B) PZR inhibition by D9a reduces tamoxifen‐induced Wnt‐dependent adenoma formation in the *Apc^LoxP/LoxP^‐Lgr5‐Cre^ERT2^‐IRES‐eGFP* transgenic mouse. A representative example (left) and quantification of the in vivo effect of D9a on microadenoma number (mean ± SEM; *n* = 4–5 ^*^ *p* < 0.05 Student *t* test) and area (3‐29 adenoma per mouse). Shown is the IHC analysis of GFP expression in the transformed intestine with microadenomas and their area highlighted. C) D9a increases intestinal tumor cell apoptosis. A representative example (top) and quantification (mean ± SEM, *n* = 4 mice; ^*^ *p* < 0.05) (bottom) of IHC analysis of cleaved caspase‐3, a marker of cell apoptosis. D) pSHP2/pSRC/β‐catenin/JAG1 signaling is reduced in transformed intestinal epithelium of D9a‐treated mice. IHC analysis of pSHP2, pSRC, active nuclear β‐catenin and JAG1 protein levels from Tam‐induced adenoma of D9a (or control Iso)‐treated mice. Left: a representative example; right: quantification (% of positive cells) in intestinal adenoma (mean ± SEM of 10 are/mouse, *n* = 4–5 mice per group; ^*^ *p* < 0.05; ^**^ *p* < 0.01; ^***^ *p* < 0.001 Mann–Whitney test). E) Workflow to evaluate the PG‐dependent PZR signaling in adenoma. Increased PZR phopho‐signaling during Wnt‐dependent adenoma formation is affected in anti‐PG treated mice. F) pPZR, pSHP2, pSRC, active nuclear β‐catenin and JAG1 protein levels (% of positive cells) in the intestine of treated mice as shown (mean ± SEM of 10 area/mouse, *n* = 4 mice per group; *p* value >.0.05 are indicated; ^*^ *p* < 0.05; ^**^ *p* < 0.01; ^***^ *p* < 0.001 Mann–Whitney test).

To investigate the anti‐CSC‐like activity of D9a in vivo, we used the *Apc^LoxP/LoxP^‐Lgr5 Cre^ERT2^‐IRES‐eGFP* transgenic mouse model, which induces Wnt‐dependent intestinal adenomas only after 2 weeks of tamoxifen treatment^[^ ^61^ ^]^. Tamoxifen induces biallelic inactivation of the *Apc* gene in Lgr5‐positive cells in vivo, resulting in Wnt/β‐catenin‐dependent intestinal stem cell (ISC) transformation (**Figure** **7**A). Daily treatment with D9a specifically reduced the number and size of the microadenomas (Figure 7A,B). An increase in epithelial cell apoptosis was detected in the microadenomas from the D9a‐treated mice (Figure 7C). Next, IHC analysis revealed activation of pPZR/pSHP2/pSRC signaling along with increased JAG1 protein levels during intestinal transformation, which was inhibited by D9a (Figure 7D). This antibody also reduced the transcript level of the CSC‐like marker CD44 (including its variants), consistent with the anti‐CSC‐like activity of PZR (Figure S19A, Supporting Information); however, only a few selected oncogenic β‐catenin target genes were affected by D9a (Figure S19B, Supporting Information), possibly because of the residual PZR signaling that was not targeted by D9a as well as too high levels of Wnt/β‐catenin activity induced by biallelic *Apc* inactivation. Nevertheless, the protein level of JAG1 was inhibited by D9a, confirming the PG‐dependent inhibitory effect of PZR inhibition (Figure 7D). These data are consistent with a pro‐tumorigenic function of PG established in Wnt/β‐catenin‐dependent intestinal transformation.^[^ ^32^, ^39^, ^40^ ^]^ To further investigate PG‐dependent PZR tumor signaling in this model, mice were treated with PG antibody one day prior to analysis (Figure 7E). In vivo PG inhibition resulted in a significant reduction in active pSHP2, pSRC, and JAG1 tumor levels (Figure 7F). A trend toward inhibition of pY263‐PZR level was also observed (Figure 7F). Collectively, these data support a PG‐dependent PZR signaling function during intestinal tumorigenesis that can be targeted with an anti‐PZR mAb.

**Figure 8:**
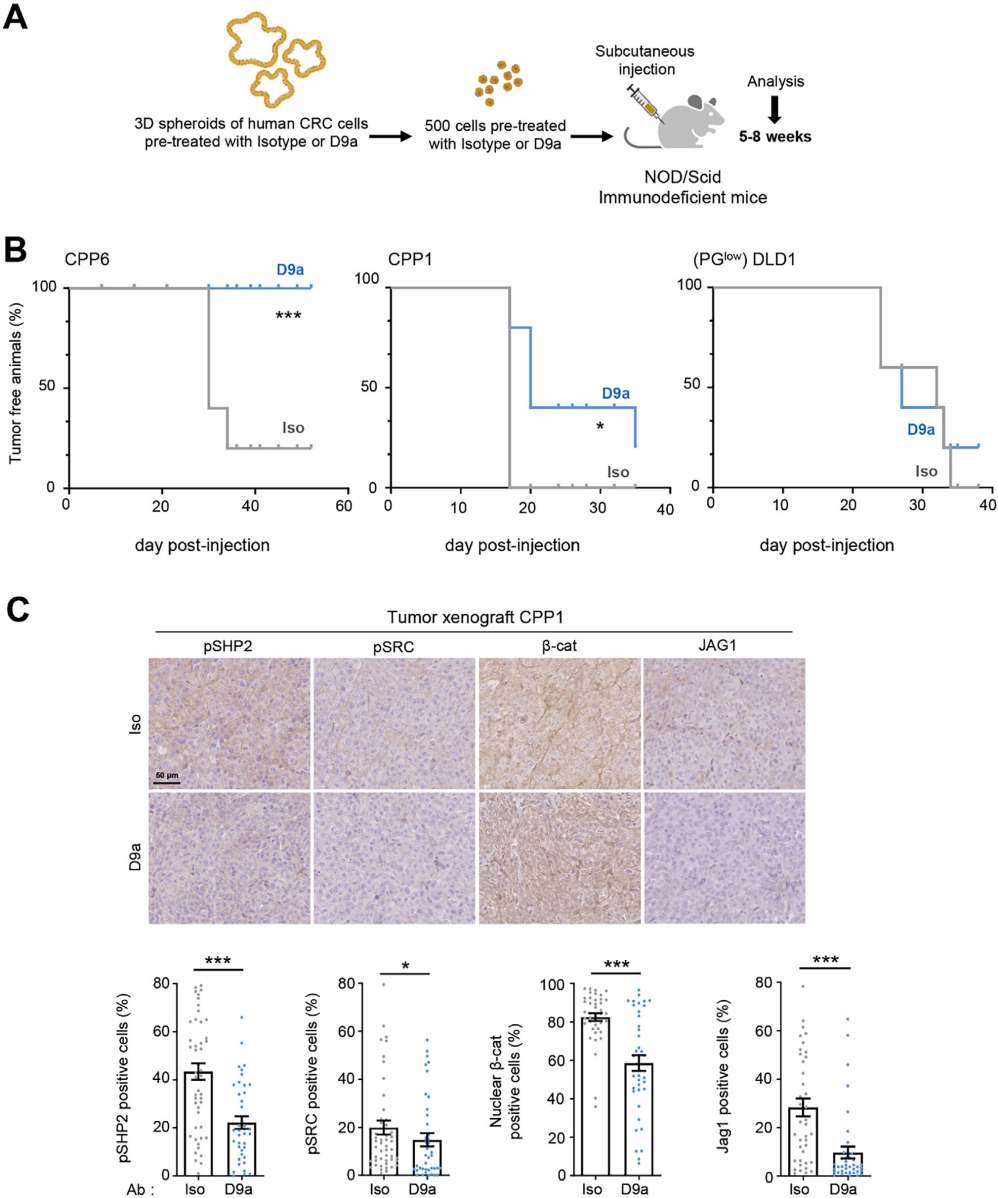
Antibody‐mediated PZR inhibition reduces tumor the initiating capacity of PG‐high CRC cells in immunodeficient mice. A) Workflow to evaluate the contribution of PZR to the tumor‐initiating capacity of human CRC cells in immunodeficient mice. B) D9a reduces tumor the initiating capacity of PG‐high (PG^high^) CRC cells inoculated into SCID mice. Kaplan‐Meyer plot of tumor‐free animals injected with indicated CRC cells over time (mean ± SEM, *n* = 5 mice per group; ^*^ *p* < 0.05; ^***^ *p* < 0.001 Mann–Whitney test). C) pSHP2/pSRC/β‐catenin/JAG1 signaling is reduced in tumors derived from D9a‐pretreated CPP1 cells. A representative example (top) and quantification (% positive cells) of CPP1 tumors (mean ± SEM of 10 area analyzed, *n* = 4 mice per group; ^*^ *p* < 0.05; ^**^ *p* < 0.01; ^***^ *p* < 0.001 Mann–Whitney test).

Finally, to investigate the anti‐CSC‐like activity of D9a on patient‐derived CRC lines in vivo, we evaluated the tumor‐initiating capacity of D9a pre‐treated colonospheres in NOD/Scid mice. Subcutaneous injection of 500 isolated cells resulted in detectable tumors 15–30 days after cell inoculation (**Figure** **8**A). In contrast to Iso treatment, D9a treatment strongly affected the tumor‐initiating capacity of the PG‐high CRC line CPP6, with no detectable tumors observed even 58 days after injection (Figure 8B). A significant inhibitory effect was also observed in the patient‐derived CPP1 CRC line,^[^ ^44^ ^]^ which expresses lower levels of PG (Figure 8B; Figure S17A, Supporting Information). In contrast, no D9a inhibitory effect was observed in PG‐low DLD1 cells (Figure 8B). IHC analysis of CPP1 tumors xenografts revealed that D9a treatment led to reduced tumor cell proliferation, angiogenesis (Figure S20A,B, Supporting Information) and decreased pSHP2/pSRC/β‐catenin signaling and JAG1 protein levels in CPP1 tumors (Figure 8C). Thus, D9a also reduced in vivo CSC‐like properties of human CRC cells.

## 3 Discussion

Tumor relapse and metastatic spread caused by intratumor heterogeneity remain major clinical issues in CRC.^[^ ^4^, ^5^ ^]^ The tumor microenvironment plays a pivotal role in this pathological process by promoting the CSC traits of CRC cells.^[^ ^4^, ^5^ ^]^ Elucidating the signaling mechanisms involved in this neoplastic process may be of therapeutic interest in treating advanced CRC.

Progastrin (PG) is a growth factor peptide secreted in the CRC microenvironment that promotes colonic CSC‐like functions. By investigating the mechanism by which PG induces SRC‐like signaling in CRC cells, we identified PZR as an essential receptor for PG to induce this neoplastic feature. This finding was demonstrated using several in vitro and in vivo experimental tumor models. Specifically, we demonstrated that PZR mediates the binding of PG to CRC cells, and a direct PG–PZR interaction was confirmed in HEK293T cells co‐expressing both proteins. PG–PZR signaling promotes colonic epithelial cell proliferation under physiological conditions and drives the expression of CSC‐like markers and behaviors in both murine intestinal tumor cells and human CRC cells in vitro. Genetic inactivation of PZR significantly reduced CSC‐like traits in murine intestinal cells and in CRC cells with high PG expression in vivo. Furthermore, PZR regulates PG‐dependent activation of Wnt/β‐catenin and Notch signaling pathways, both known to contribute to CSC‐like functions.^[^ ^42^ ^]^ Although not investigated in more detail, our results indicate that PZR may mediate additional reported functions of PG in CRC, including angiogenesis^[^ ^43^ ^]^ and tumor cell migration^[^ ^37^ ^]^. In agreement with this notion, PZR was shown to induce cancer cell migration in a large number of epithelial cancers^[^ ^25^, ^26^, ^27^, ^62^ ^]^. Collectively, these results may explain the functional link between *GAST* and *MPZL1* expression in terms of progression‐free survival and disease‐specific survival observed in primary colon cancer patients.

Our study revealed a complex mechanism by which PG induces PZR signaling in CRC cells. The cellular PZR‐PG interaction was influenced by N‐glycosylation and the dimeric/multimeric state of the receptor. Aberrant N‐glycosylation contributes to tumor cell metabolism, which impacts the CSC‐like properties of tumor cells^[^ ^63^ ^]^. Interestingly, PG reportedly regulates glycolytic activity in CRC CSCs to promote their self‐renewal ability.^[^ ^44^ ^]^ These findings raise the possibility that PG‐PZR signaling may sense the metabolic state of CSCs to maintain their glycolytic state via PZR N‐glycosylation. Another layer of PG signaling complexity arises from the observation that PG produced by CRC cells undergoes serine/threonine phosphorylation and tyrosine sulfation.^[^ ^40^ ^]^ It is anticipated that these chemical modifications may also influence PG‐PZR interactions and receptor signaling. Like many TK‐associated receptors, PZR self‐association and/or association with another receptor may be an important mechanism of receptor signaling.^[^ ^64^, ^65^ ^]^ Although our study revealed that PG activates PZR phospho‐signaling, this finding does not exclude the possibility that an additional receptor or co‐receptor promotes PZR dimerization and signaling. In agreement, MET was recently reported to interact with PZR to induce glioblastoma cell migration.^[^ ^66^ ^]^ Similarly, we cannot exclude the possibility that additional receptors, notably G‐protein coupled receptors (GPCRs), may contribute to the protumor functions of PG in CRC, as previously reported.^[^ ^38^, ^48^ ^]^ In support to this notion, 2 orphan GPCRs were identified as SRC substrates in SW620 cells through phospho‐proteomic analyses^[^ ^50^ ^]^, which could potentially participate in SRC oncogenic signaling induced by PG.

PZR has been identified as an SHP2 interactor and an SRC substrate, two key signaling proteins activated by PG in the colonic mucosa^[^ ^22^, ^36^ ^]^. In vitro, PZR promotes CSC‐like properties primarily through SHP2/SRC/β‐catenin/Notch signaling. Additionally, PZR functions as an upstream activator of β‐catenin/TCF4 signaling, which is implicated in the CSC‐like behavior of lung cancer cells ^[^ ^28^ ^]^. These findings align closely with our proposed model of a PG/PZR/SHP2/SRC/β‐catenin signaling axis driving CSC‐like renewal in the colon. Mechanistically, SHP2 enhances SRC oncogenic activity by dephosphorylating its negative regulators, such as CBP/PAG, which are CSK‐binding proteins, in CRC.^[^ ^19^ ^]^ SHP2 acts in a similar way to potentiate β‐catenin oncogenic function, notably through protein stabilization via Parafibromin dephosphorylation^[^ ^67^ ^]^. Active SRC further promotes β‐catenin signaling by direct phosphorylation and/or phosphorylation of YAP, a crucial nuclear interactor and transcriptional co‐activator ^[^ ^68^ ^]^, an important nuclear interactor and transcriptional co‐activator, both of which may contribute to the maintenance of the PZR/SRC/β‐catenin axis in CRC cells. Given that PG is a known β‐catenin/TCF4 target gene, our results support the existence of a feedforward loop involving PG, PZR, SRC, and β‐catenin that sustains CSC‐like functions in CRC. Finally, prior genetic studies in animal models have shown that SRC promotes ISC proliferation during tissue self‐renewal, regeneration, and tumorigenesis through mechanisms that remain incompletely understood.^[^ ^11^ ^]^ Our findings position PZR as a novel upstream activator of SRC in regulating intestinal CSC‐like activity.

The results obtained with our PZR antibody suggest a new therapeutic strategy aimed at inhibiting the CSC properties of PG‐high CRC cells. Our experiments in a Wnt‐dependent tumor mouse model validated the antitumor effect of our PZR mAb in an immunocompetent cancer model. Interestingly, an anti‐PG mAb was developed that could decrease the self‐renewal of colonic CSCs both in vitro and in vivo, either alone or in combination with chemotherapy.^[^ ^39^, ^40^ ^]^ This report, together with our study, suggests anti‐CSC‐like activity of these mAbs also in KRAS‐mutated CRC cells, which are known to be resistant to anti‐EGFR mAbs^[^ ^3^ ^]^. Taken together, these results support the therapeutic potential of PG‐PZR mAbs in advanced CRC. However, we anticipate an additional antitumor effect of the PZR mAb, which may also target the PG‐independent CSC‐like function of PZR caused by aberrant PZR expression and/or SRC overactivation. The fact that *Mpzl1* mouse genetic inactivation did not result in any major defect under normal conditions precludes of any severe toxicity with PZR inhibitors. Collectively, these results increase the potential therapeutic interest of PZR mAb in PG‐high CRC.

Pro‐tumor functions of PZR have been reported in many other tumors, including lung cancer and HCC, notably MPZL1‐amplified tumors.^[^ ^25^, ^26^, ^28^, ^69^, ^70^ ^]^ These reports, together with our study, suggest that PZR mAb may have an anti‐tumor effect in these cancers as well. Interestingly, these tumor cells also express PG, and circulating levels of this peptide have been proposed as a promising biomarker for disease monitoring.^[^ ^46^, ^71^ ^]^ We therefore propose that PG secreted in their microenvironment may also contribute to these PZR tumor functions, similar to what we observed in CRC, and that circulating PG levels may serve as a biomarker of anti‐tumor activity of PZR inhibitors.

## 4 Conclusion

In conclusion, our study proposes the orphan receptor PZR as an essential receptor for the tumor microenvironment orphan peptide PG to induce colon CSC‐like features by promoting SHP2/SRC/β‐catenin signaling. By developing a PZR antibody that successfully reduced adenoma formation upon loss of *Apc* in mouse intestinal stem cells and the tumor‐initiating capacity of patient‐derived PG‐high CRC cells in immunodeficient mice, our work suggests that PZR antibodies could be of therapeutic interest in CRC, and the circulating PG level is a potential biomarker.

## 5 Experimental Section

### Reagents

Recombinant PG (rPG) was produced in E. Coli essentially as described in.^[^ ^44^ ^]^ Briefly, His‐SUMO‐PG fusion protein was induced by IPTG induction for 4h à 37 °C and affinity‐purified on a Ni‐NTA column (Cytiva) followed by SUMO protease cleavage to remove the His‐SUMO sequence. G‐17, recombinant human JAG‐1, and Concanavalin A peptides were from Sigma. Purified PZR‐EXT‐His (#PKSH032771) was from Elabscience and EGFR‐EXT‐His from Sinobiological (#10001‐H08H). Polyclonal rabbit anti‐PG antibodies production and purification on an affinity column was described.^[^ ^44^ ^]^ Anti‐PG N‐terminus (anti‐PG N) was raised against the *GAST* products peptides corresponding to pre‐progastrin 22–35 AA, and anti‐PG C‐terminus against 93–101 AA. The mixture of anti‐PG N+C (1:1) was used for functional studies (anti‐PG). Non immune control rabbit IgG was from Sigma. mAb PZR antibodies used in this study were produced in CHO cells (Evitria AG, Zürich, Switzerland) (50‐100 mg), control Iso isotype was purchased at Leinco Technologies (#I‐118) and cetuximab was from Merck (Erbitux). Antibody used in for biochemistry: anti‐Src pY419 SRC (#2101L), anti‐SRC (clone 327 #ab16885, Abcam) anti‐Myc (#2276S), anti‐PZR (#9893), anti‐pY263 PZR (#8088) and anti‐pY241 PZR (#8131), anti‐SHP2 (#3397), anti‐pY541 SHP2 (#3751) were from Cell Signaling Technology (CST), anti‐FLAG (M2 antibody, Sigma), anti‐HA (2‐2.2.14 In Vitrogen, # 26183), anti‐tubulin (gift from N. Morin, CRBM, Montpellier, France), anti‐Actin (Sigma #A2228), anti‐pTyr 4G10 (gift from P. Mangeat, CRBM, Montpellier, France). Small inhibitors used in this study: ABL inhibitor nilotinib (MedChemExpress), SRC‐like inhibitors bosutinib (1 µm, MedChemExpress) and AZD0530 (1 µm, MedChemExpress), SRCi eCF506 (0.1 µm, MedChemExpress #HY‐112096) and the SHP2 inhibitor SHP2i (1 µm, SHP099 Selleckchem), CCKBR inhibitor (1 µm L365260 – Tocris Bioscience), Wnt inhibitor (10 µm IWR1 – Sigma–Aldrich) and Notch inhibitor (1 µm LY411575 – Sigma–Aldrich). pT7‐HIS‐SUMO was from Novopro (V015042). Human PZR‐FLAG pcDNA3 expression vector was a gift of Dr Bennet (Yale University School of Medicine, New Haven, USA).^[^ ^24^ ^]^ PZR mutants were obtained using the QuickChange Site‐Directed Mutagenesis Kit (Agilent) using oligonucleotides listed in Table S1 (Supporting Information). PZR tagged with FLAG, Myc and HA sequence were inserted in pCDNA3 and retroviral pMX‐pS‐CESAR expression vectors. Pol, GAG, Env and SRC (pMX‐SRC) expression vectors were described in ref. [16], retroviral pRETROSUPER‐GFP‐shGAST in,^[^ ^43^ ^]^ and inducible shGAST pTRIPZshGAST (Dox‐shGAST) in.^[^ ^44^ ^]^ PZR‐EXT‐Fc expression construct was produced by inserting PZR extracellular domain (PZR S36‐V162, PZR‐EXT in pFUSE‐hlgG1‐Fc1 (Invivogen). The shRNA containing oligonucleotides directed against PZR GAGAATACCTAGAACATAT was inserted in to pSUPER‐neoGFP‐shPZR construct (shPZR). siRNA sequence used in this study are listed in Table S2 (Supporting Information).

### Biochemistry

Co‐immunoprecipitation and Western Blotting (WB) were described in ref. [16] Briefly, cells were lysed at 4 °C with RIPA buffer (20 mm Tris‐HCl pH 7.5, 150 mm NaCl, 1 mm EDTA, 1% NP‐40, 1% sodium deoxycholate) or lysis buffer (20 mm Hepes pH 7.5, 150 mm NaCl, 0.5% Triton X‐100, 6 mm β‐octylglucoside) supplemented with complete mini‐EDTA free protease and phosphatase inhibitor cocktail tablets (ROCHE). 15–35 µg of protein lysates were loaded on SDS‐PAGE gels, transferred to PVDF membranes using Trans‐Blot® Turbo™ (Bio‐Rad), and then incubated overnight at 4 °C with the appropriate primary antibody (1:1000 dilution) and for 45 min with the secondary HRP‐conjugated antibody (Cell Signaling, 1:4000 dilution). Signal detection was performed using the ECL Plus reagent (Amersham Biosciences) and the Amersham Imager 600 system (GE Healthcare Life Sciences). Signal quantification and analysis were performed using ImageJ software. For PZR self‐association experiments, HEK293T cells were co‐transfected with indicated Flag‐tagged and HA‐tagged PZR constructs for 40 h. Cell‐lysates were subjected to PZR‐FLAG immunoprecipitation using anti‐FLAG magnetic beads (#A3697, Pierce) and the presence of PZR‐HA was detected by anti‐HA WB. PZR deglycosylation assays were performed as described in ref. [22] using 1h treatment with purified PNGase F (Sigma) before PZR WB analysis. Raw blots images are shown in Figure S21 (Supporting Information).

### Cell Cultures, Retroviral Infections and Transfections

Cell lines (HEK293T, DLD1, RKO, HCT116, HT29, SW480, SW620, T84, JMT1) (ATCC, Rockville, MD), SRC‐overexpressing CRC cells (HCT116 and SW620)^[^ ^16^, ^50^ ^]^ and patient‐derived CRC lines (CPP1, 6, 19, 43 and 44)^[^ ^52^ ^]^ were cultured at 37 °C and 5% CO2 in a humidified incubator in DMEM (Life Technology) supplemented with 10% FCS and 1% Penstrep and checked for mycoplasma once a week using the MycoAlert detection kit (LT07‐318, Lonza). Cells were transfected with jetPEI Polyplus according to the manufacturer's instructions and infected as described in ^[^ ^16^, ^50^ ^]^ Retroviral production and cell infection were performed as described.^[^ ^16^, ^50^ ^]^ Cells expressing pTRIPz inducible lentiviral short hairpin RNA containing oligonucleotides directed against GAST mRNA (Dox‐shGAST)^[^ ^44^ ^]^ were treated with doxycyline (1 µg mL^−1^) for 24 h to induce shRNA GAST expression. For siRNA studies, cells were transfected with Lipofectamine RNAiMAX (Invitrogen) or INTERFERin (Polyplus) according to the manufacturer's recommendations.

### PG Cellular Binding and Proximity Ligation Assay (PLA)

For PG cellular binding, CRC cells were plated on glass coverslips or in 8‐well Lab‐Tek.II chamber slides were infected or transfected with mutant constructs. Cells were incubated at 4 °C for 1 h and treated with 10 nm rPG in the presence or absence of G‐17 and concanavalin‐A (indicated concentrations) for 1 h before fixation (4% paraformaldehyde for 10 min). Slides were incubated with primary antibody (rabbit anti‐PG N‐terminal) for 1 h at room‐temperature and then with fluorescent anti‐rabbit secondary antibody conjugated to Alexa‐488 or cyanin3 and Hoechst (2 µg mL^−1^, Sigma–Aldrich). rPG cell binding was analyzed by fluorescence using an upright fluorescence microscope. PLA was performed according to the kit protocol (#DUO92101, MERCK). HEK293T cells plated on glass coverslips were transfected with mock, PZR‐Flag, PZR 2NQ‐Flag, PZR‐myc, and PZR mono‐Myc mutant constructs for 48 h and fixed with 4% paraformaldehyde, 0.5% Triton for 20 min. In some experiments, cells were incubated at 4 °C for 1 h and treated with 10 nm rPG for 1 h prior to fixation. Glass slides were blocked in Duolink® Blocking Solution for 1 h at 37 °C and then incubated with primary antibodies in Duolink® Antibody Diluent for 1 h at room‐temperature (Table S3, Supporting Information). After washing, the slides were incubated with PLUS and MINUS PLA diluted in Duolink® Antibody Diluent (1:5 dilution) for 1 hat 37 °C. After three washes, the slides were incubated in the ligation solution during 30 min at 37 °C and the amplification solution for 100 min at 37 °C. Finally, after washes, the slides were mounted with the Duolink® In Situ Mounting Medium with DAPI and observed with an upright fluorescence microscope.

### Analysis of CSC Markers

For CD44v6 and CD26 staining, 200 000 CRC cells were treated with isotype control antibody conjugated to APC (Biotechne, 1:100) and CD44v6 antibody anti‐human conjugated to APC (Miltenyi Biotec, 1:300 dilution) or CD26 antibody anti‐human conjugated to APC (Miltenyi Biotec, 1:300 dilution) for 15 min at 4 °C and washed. Cells were treated with Sytox blue (Thermofisher, 1:300) and analyzed by flow cytometer. The ALDH assay was performed according to the protocol of the ALDEFLUORTM kit (#01700, Stemcell).

### Colospheres Formation

100 cells per well were seeded in ultra‐low adherent 96‐well plates (Corning) in 100 mL DMEM/F12 (Life technologies) supplemented with 1% Penstrep, 2 mm L‐glutamine, N‐2 (Life technologies), EGF (20ng mL^−1^ – Bio‐techne) and FGF (10ng mL^−1^ – Bio‐techne). After 7 days, photographs of each sphere were taken and sphere size was measured using ImageJ software.

### Organoids Derived from Colonic Crypts

The colons were collected from WT and Mpzl1tm1.1(KOMP)Vlcgmice (Mpzl1‐/‐ mice) and washed with PBS containing antibiotics. They were then cut open and further dissected into small pieces then washed with a wash buffer containing PBS1X supplemented with 1% Penstrep and 1% fungin (Invivo Gen) until the supernatant is clear. These pieces were then incubated in a crypt isolation buffer for 20 min on ice (Wash buffer, 1% BSA fraction and 25 mm EDTA). Crypt fractions were isolated after vigorous pipetting then centrifuged at 300g, 4°C for 5 min. 500 crypts were resuspended in a mixture M1 media (Advanced DMEM/F12 supplemented with 1% Penstrep, 1% fungin, 10 mm HEPES, 1% Glutamax (Gibco), N‐2 (Life technologies), B‐27 (Life technologies), 1 mm N‐acetyl cysteine (Sigma–Aldrich)) and Matrigel (Corning) (ratio 1:2). The mixture was plated in a 24 well plate. After polymerization of Matrigel, 500 µL of M2 media (M1 media supplemented with EGF (50 ng mL^−1^, Bio‐techne), Noggin (100 ng mL^−1^ – Stem cell technologies), R‐spondin1 (500 ng Ml^−1^ – Stem cell technologies), Y27 (10 µm – Sigma–Aldrich), CHIR‐99021 (3 µm – Tebu‐Bio) and WNT3a (50 ng mL^−1^, Thermo Fisher Scientific)) was added to each well containing or not the recombinant PG (100 nm) and CCK2 inhibitor (1 µm L365260 – Tocris Bioscience).

### Tumoroid Formation

2000–5000 human CRC cells were resuspended in Advanced DMEM/F12 supplemented with 1% Penstrep, 2 mm L‐glutamine, N‐2 (Life Technologies), and Matrigel (Corning) (1:2 ratio) prior to plating in 24‐well plates. After polymerization of Matrigel, 0.5 mL Advanced DMEM/F12 supplemented with EGF (20 ng mL^−1^, Biotechne) and FGF (10 ng mL^−1^, Biotechne) was added. Tumoroids were cultured at 37 °C and 5% CO2 in a humidified incubator. Tumoroids were treated with specific antibody or drug (2/week) for 7 days and then analyzed: Mouse isotype control antibody (10 or 50 µg mL^−1^, Leinco Technologies), mouse D9a antibody (10–50 µg mL^−1^), rabbit IgG control antibody (50 µg mL^−1^), rabbit anti‐PG antibody (50 µg mL^−1^), nilotinib (0.1 µm), bosutinib (1 µm), SHP2 inhibitor (1 µm), cetuximab antibody (10 µg mL^−1^), and recombinant human Jagged‐1 protein (1 µg mL^−1^). For BrdU experiment, tumoroids received a BrdU pulse (2 h 10 µm) before analysis. For mouse tumoroids, colonic tumors were microdissected and collected from Mpzl1tm1.1(KOMP)Vlcg and WT mice after colitis‐associated carcinogenesis treatment or from *Apc* *^L^ ^oxP/+^‐Villin‐Cre^ERT2^* conditional mouse model treated with tamoxifen. Mice were sacrificed 2 months after tamoxifen administration. CRC cells were dissociated using the Tumor Dissociation Kit mouse (Miltenyi Biotec). Dissociation was stopped by Advanced DMEM/F12 medium with 10% FCS. 10 000 cells were resuspended in Advanced DMEM/F12 supplemented with 1% Penstrep, 10 mm HEPES, and 2 mm L‐glutamine and Matrigel (Corning) (1:2 ratio) before plating in 24‐well plates. After polymerization of Matrigel, 0.4 mL Advanced DMEM/F12 supplemented with B‐27 (Life technologies), N‐2 (Life technologies), N‐acetylcysteine (1 mm – Sigma–Aldrich), EGF (50ng mL^−1^ – Bio‐techne) and FGF (10ng mL^−1^ – Bio‐techne) was added. Tumoroids were cultured at 37 °C and 5% CO2 in a humidified incubator. In some experiments, mouse tumoroids were treated with antibody for 7 days and then analyzed.

### RNA Extraction and qPCR

Total RNA was extracted from snap frozen tissues using TRIzol reagent (Invitrogen), and RNA was purified from cell or tumoroid cultures using RNeasy Mini Kit columns (Qiagen). First‐strand cDNA synthesis was performed with 1 µg of purified RNA using cDNA synthesis KIT (Thermo Fisher Scientific) according to the manufacturer's instructions. qRT‐PCR experiments were performed using LightCycler 480 SYBR Green I Master (Roche Diagnostics) on the LightCycler 480 system. The human and mouse primer sets used are shown in Table S4 (Supporting Information). Data were normalized to the expression levels of *Gapdh*, *Hprt*, *Mrpl32* and *Actin*. Relative expression was determined using the threshold cycle relative quantification method.

### Anti‐PZR Antibody Development

Three anti‐PZR human scFv antibodies were selected by phage display from the human scFv phage display library Husc I^[^ ^72^, ^73^ ^]^ after sequential panning using the PZR‐EXT‐Fc that was affinity‐purified on Protein A sepharose (Sigma) from cell‐lysates of HEK293T cells transfected with pFUSE‐PZR‐EXT‐hlgG1‐Fc1 construct. Antibodies were first expressed in the murine IgG2a format. The afucosylated D9a mAb was selected based on its inhibitory effect on PZR tyrosine phosphorylation and colonospheres formation of the PG‐high HT29 cell‐line.

### ELISA and FACS Analysis

For PZR mAb selection, ELISA plates (NUNC‐IMMUNO Maxisorp, Thermofisher) were coated with recombinant human MPZL1 protein (Elabscience) or recombinant human EGFR protein (Sinobiological) in PBS‐1X at 4 °C overnight (1 µg mL^−1^). Plates were washed and blocked with PBS‐1 × 0.1% Tween 5% BSA and incubated at room‐temperature for 1 h. Plates were treated with primary antibodies (isotype control and D9a) for 30 min at room‐temperature (indicated concentrations), and after washing, plates were incubated with anti‐mouse secondary antibodies conjugated to HRP (Cell Signaling, 1:10 000 dilution) for 30 min at room‐temperature. After washing, 100 µL TMB ELISA solution (Thermo Fisher) was added to each well and incubated for 5 min at room‐temperature. Then, 50 µL stop solution (H2SO4 2N) was added to each well and to the plate. The optical density of each well was determined using a Polarstar Omega multimode microplate reader. Signal Intensity = PZR‐His Intensity – EGFR‐EXT‐His Intensity. For FACS analysis, tumor cell lines were gently dissociated using Versene, and 500 000 cells were resuspended in 100 µL PBS‐1 × 5% FCS. Cells were treated with primary antibodies (isotype control and D9a antibody, 50 µg mL^−1^) for 1 h and 30 min at 4 °C, and after washing steps, cells were incubated with fluorescent anti‐mouse secondary antibodies conjugated to Alexa‐647 (Jackson ImmunoResearch Laboratories, 1:200 dilution) and Zombie NIR (Biolegend, 1:1000 dilution) for 1 h at 4 °C. Samples were run through the Novocyte ACEA 2 fluorescence‐activated cell sorting flow cytometer, and data were analyzed using NovoExpress software.

To detect PG in 5‐day conditioned medium from sphere cells (300 000 cells), 25 mL of M11 conditioned medium were concentrated 25‐fold using a 3 kDa cut‐off concentrator (Sigma–Aldrich). Progastrin quantification was then performed using the Human PG80 (Circulating Progastrin) ELISA Kit (Ozyme, AEFI00266) according to the manufacturer's instructions.

### Animal Experiments

All animal studies were performed in strict accordance with the guidelines of the European Community (Directive n°2010/63/EU) and approved by the French National Committee (APAFIS #32956‐2021081611417690 v3 and #201608021520787). All transgenic mice were on a C57BL/6 genetic background. Animals were maintained under pathogen‐free conditions in the animal facility of the institute. Male and female mice were analyzed between 10 and 16 weeks of age. To study the impact of MPZL1 loss in homeostasis and during colitis‐associated carcinogenesis, Mpzl1tm1.1(KOMP)Vlcg mice Mpzl1‐deificient mice were generated by the trans‐NIH Knockout Mouse Project (KOMP) and obtained from the KOMP repository. For AOM/DSS experiments, mice were injected intraperitoneally with azoxymethane (10 µg g^−1^ AOM of body weight) followed by three cycles of 2% (w/v) DSS in the drinking water. Mice were not treated for 2 weeks between cycles. Mice were sacrificed at the indicated time points. To investigate the anti‐tumor activity of the antibody, we crossed *Apc^LoxP/LoxP^* mice with the *Lgr5‐Cre^ERT2^‐IRES‐eGFP* knock‐in (KI) mouse model to generate the *Apc^LoxP/LoxP^‐Lgr5‐Cre^ERT2^‐IRES‐eGFP* ^[^ ^60^ ^]^. The *Apc^LoxP/+^*‐*Villin‐Cre^ERT2^* conditional mouse model was generated by crossing *Apc^LoxP/+^* mice with *Villin‐Cre^ERT2^* mice^[^ ^60^ ^]^. Tamoxifen (Sigma–Aldrich) was administered to induce APC deletion by intraperitoneal injection at a single daily dose of 1 mg for 3 days. Female Athymic Nude mice and NOD/scid mice were obtained from Charles River Laboratories. 500 (NOD/scid mice) or 1 000 000 cells (nude mice) were injected subcutaneously into the right flank of immunodeficient mice in a 1:1 mixture of Matrigel and with a final volume of 100 µL. Tumor detection was recorder over time and development (tumor volume ([length × width^2^]/2) was measured with a caliper. Mice were sacrificed when tumors reached 1 500 mm^3^.

### Genotyping

Tail punch biopsies were lysed in alkaline lysis reagent and incubated at 92 °C for 20 min. The reaction was stopped with neutralization reagent. PCR was performed using MyTaq HS DNA Polymerase Red Mix (Bioline). Samples were analyzed on a 2% agarose gel using ethidium bromide (Sigma–Aldrich), 1 kb DNA ladder (Thermo Scientific), and a ChemiDoc MP imager (Bio‐Rad). Genotyping primer sequences are provided in Table S5 (Supporting Information).

### Bright Field IHC and Fluorescence on Paraffin‐Embedded Tissue

Organs and tumoroids were fixed in 10% neutral buffered formalin (NBF) for 24 h, embedded in paraffin, and sectioned at 4 µm. Sections were incubated in successive baths of xylene and ethanol, and antigen blocking was performed by boiling the sections in 10 mm sodium citrate (pH 6.4) or Tris‐EDTA buffer (pH 9) for 20 min. After blocking non‐specific binding with TBS‐1 × 2% serum 0.1% Triton, samples were incubated with primary antibodies overnight at 4 °C (Table S3, Supporting Information). For bright field immunochemistry, sections were treated with BIOXALL (Vector Laboratories) for 10 min before blocking and incubation with primary antibodies. Secondary staining was detected with DAB (Vector Laboratories), and sections were counterstained with hematoxylin (Sigma–Aldrich) and mounted with mounting medium (Pertex). For fluorescence immunochemistry, sections were incubated with fluorescent secondary antibodies conjugated to Alexa‐488 or cyanin3 (Jackson ImmunoResearch Laboratories) and Hoechst (2 µg mL, Sigma–Aldrich) and mounted with aqueous mounting medium (Sigma–Aldrich). For histological examination, tissue sections were deparaffinized and stained with hematoxylin, eosin, and alcian blue.

### β‐galactosidase Activity

Mpzl1tm1.1(KOMP)Vlcg mouse strain contains a central targeting cassette with the lacZ reporter gene. To visualize the activity of the MPZL1 promoter driving lacZ expression, we used 5‐bromo‐4‐chloro‐3‐indolyl‐beta‐D‐galactopyranoside (Xgal, Sigma–Aldrich). Organs were cryopreserved, and 10 µm tissue sections were prepared. Sections were treated with 0.5% glutaraldehyde for 10 min at room‐temperature followed by overnight incubation at 37 °C in a staining solution containing 1 mg mL^−1^ X‐gal, 5 mm potassium ferricyanide (K3Fe(CN)6), 5 mm potassium ferrocyanide (K4Fe(CN)6) and 2 mm MgCl2, 0.1% Triton in PBS‐1X. The synthetic substrate X‐gal gives an insoluble blue precipitate when cleaved by β‐galactosidase. Samples were washed in PBS‐1X for 5 min and rinsed briefly with dH2O before mounting with aqueous mounting medium (Sigma–Aldrich).

### MPZL1/GAST Expression Analysis in CRC Patients

A cohort of 348 patients with primary CRC were selected from.^[^ ^59^ ^]^ Clinical characteristics and mRNA normalized levels were downloaded from cbioportal. 279 patients with tumor morphology classified as adenocarcinoma were retained while those with mucinous type were not included for further analysis. Patients with PZR expression lower than the median of all patients were considered ‘Low PZR’ and those with higher values “High PZR.” Patients with detectable GAST signal were classified as “GAST high.” Survival analysis was performed using “survival” R package, p‐value was calculated using the log‐rank test and visualization plots were generated using survmineR package as in.^[^ ^74^ ^]^

### Microscopy and Imaging

Histological slides were scanned using a Nanozoomer scanner (Hamamatsu) with a 40 × objective and visualized using NDP.view2 software (Hamamatsu). Fluorescence images were acquired with an AxioImager Z2 microscope (Zeiss) equipped with Zen software. Images were processed using ImageJ.

### Statistical Analysis

Statistical tests were performed using GraphPad Prism Version 9–11 using Mann–Whitney, ANOVA, or Student *t* test, with appropriate post hoc tests for multiple comparisons. In vivo survival curves were derived from Kaplan–Meier estimates, and the curves were compared by log‐rank tests. A 5% cutoff was used to validate result significance.

### Ethics Approval

Animal studies were performed in strict accordance with the guidelines of the European Community (Directive n°2010/63/EU) and approved by the French National Committee (APAFIS #32956‐2021081611417690 v3 and #201608021520787). Patient tumor‐derived cell lines of colon cancer cells were obtained from CRC patient biopsies provided by CHU‐Carémeau. The use of human specimens and informed consent were approved by the local ethic committee (Nîmes, France, ClinicalTrial.gov Identifier#NCT01577511).

## Conflict of Interest

The authors declare no conflict of interest.

## Author Contributions

M.B. and K.E. contributed equally to this work. S.R. and J.P. conceived and designed the study. J.N., M.L., M.J., M.B., K.E., R.L., Y.B., V.S., E.F., A.S., S.T., and L.B. performed experiments and acquired data. M.N. and P.M. developed the PZR antibody. C.P. and M.H. developed the *Mpzl1‐/‐* mice. N.C. and P.J. provided *Apc^LoxP/LoxP^‐Lgr5‐Cre^ERT2^‐IRES‐eGFP* and *Apc^LoxP/+^–Villin‐Cre^ERT2^* mouse models. M.M. and J.C. performed gene expression analysis in colorectal cancer (CRC) patients.

## Supporting information



Supporting Information

## Data Availability

The data that support the findings of this study are available from the corresponding author upon reasonable request.
