# Supplementary material for: Progastrin Promotes Colorectal Cancer Stem Cell‐Like Properties via the Receptor PZR
Source: Adv Sci (Weinh). 2025 Aug 3;12(38):e02136. doi: 10.1002/advs.202502136 (PMC12520572; doi:10.1002/advs.202502136)
Supplement: Supplementary file 1 — Supporting Information [file ADVS-12-e02136-s001.pdf]

ADVANCED SCIENCE
Open Access

Supporting Information

for *Adv. Sci.*, DOI 10.1002/advs.202502136

Progastrin Promotes Colorectal Cancer Stem Cell-Like Properties via the Receptor PZR

Julie Nguyen, Marie Lafitte, Maud Barbery, Kevin Espie, Maya Jeitany, Romain Larive, Jihane Vitre, Conception Paul, Yvan Boublik, Elise Fourgous, Valérie Simon, Audrey Sirvent, Madeline Neiveyans, Steeve Thirard, Lucile Bansard, Morgan Maillard, Nathalie Coutry, Jacques Colinge, Philippe Jay, Pierre Martineau, Michael Hahne, Julie Pannequin and Serge Roche**

Supporting information

Progastrin promotes colorectal cancer stem cell-like properties via the receptor PZR

Julie NGUYEN, Marie LAFITTE, Maud BARBERY, Kevin ESPIE, Maya JEITANY, Romain LARIVE, Jihane VITRE, Conception PAUL, Yvan BOUBLIK, Elise FOURGOUS, Valérie SIMON, Audrey SIRVENT, Madeline NEIVEYANS, Steeve THIRARD, Lucile BANSARD, Morgan MAILLARD, Nathalie COUTRY, Jacques COLINGE, Philippe JAY, Pierre MARTINEAU, Michael HAHNE, Julie PANNEQUIN & Serge ROCHE**

correspondence : serge.roche@crbm.cnrs.fr; julie.pannequin@igf.cnrs.fr

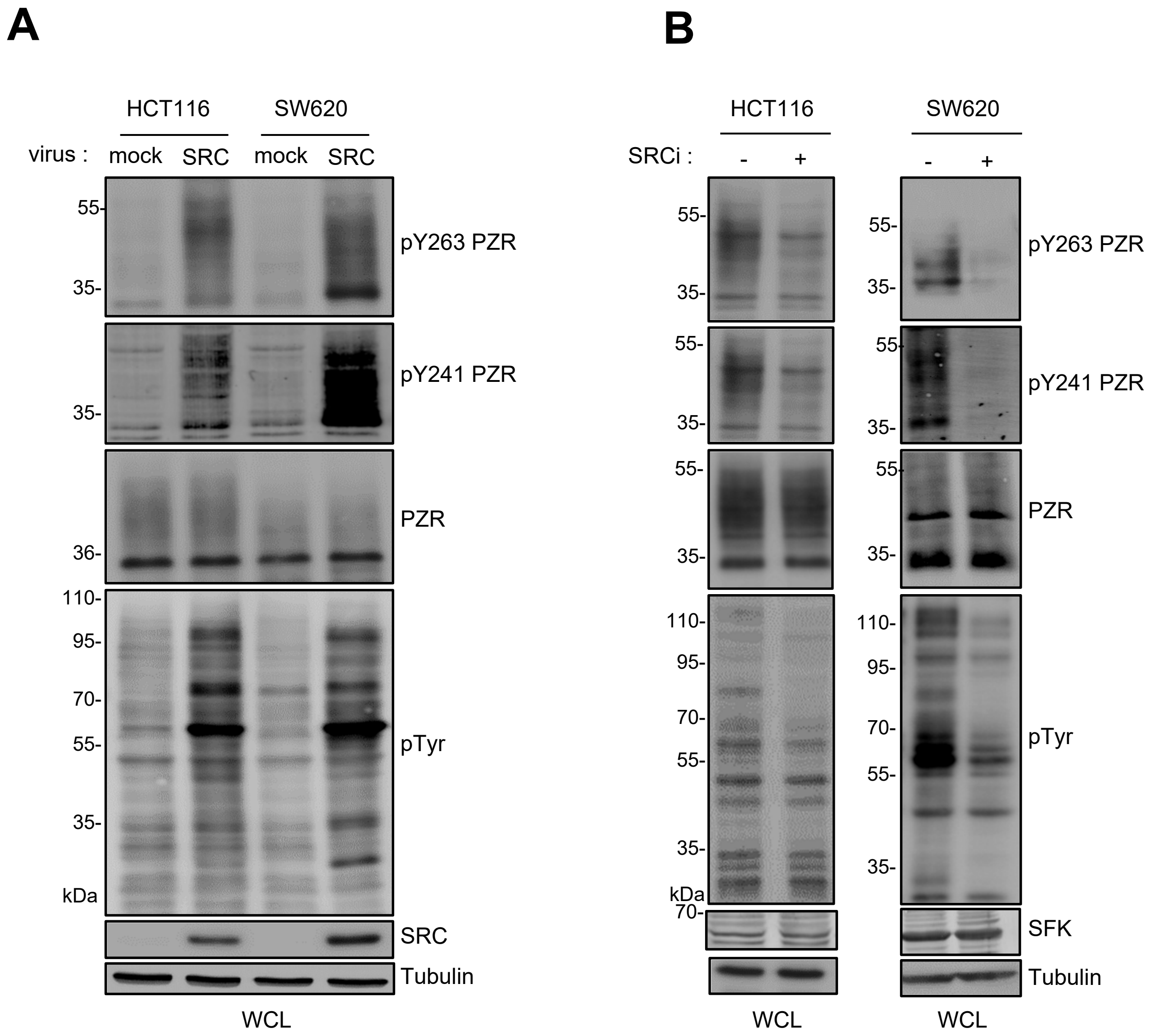

Figure S1: PZR is an SRC substrate in CRC cells. A: Phosphorylation of PZR at tyrosine residues Y241 and Y263 upon SRC overexpression. **B:** SRC-dependent phosphorylation of PZR is reduced by SRC inhibition (SRCi: 100 nM eCF506 for 1 hour). WCL: whole cell lysate. Molecular weights (MW) are indicated (kDa).

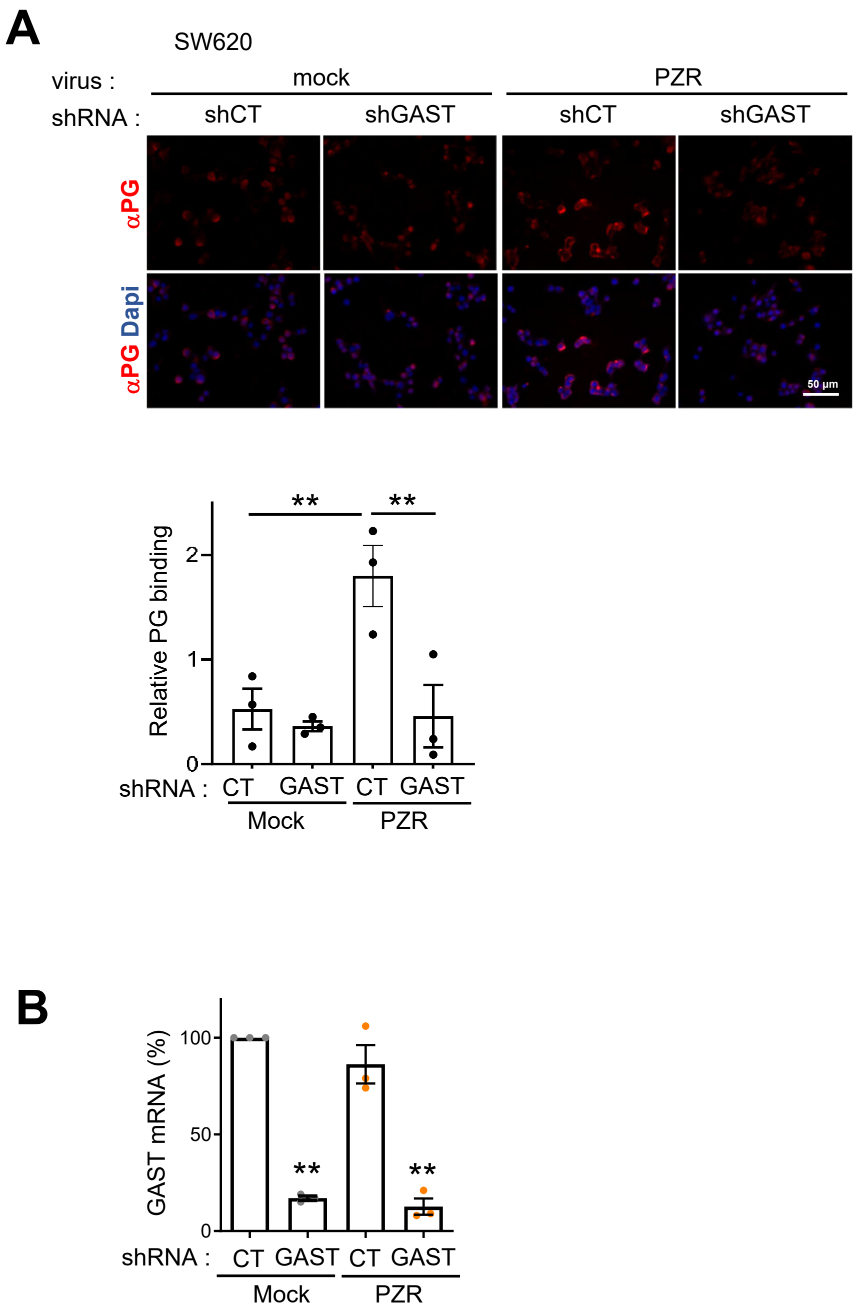

Figure S2: PZR mediates endogenous PG binding to CRC cells. **A**: Representative immunostaining (top) and quantification (bottom) of cellular PG binding (relative intensity/cell) on SW620 cells transduced with the indicated constructs, as described in Figure 1. **B**: The level of GAST transcript (% control) and nuclear cell staining (Dapi) is shown (mean ± SEM, n=3; **p<0.01; Student's t-test).

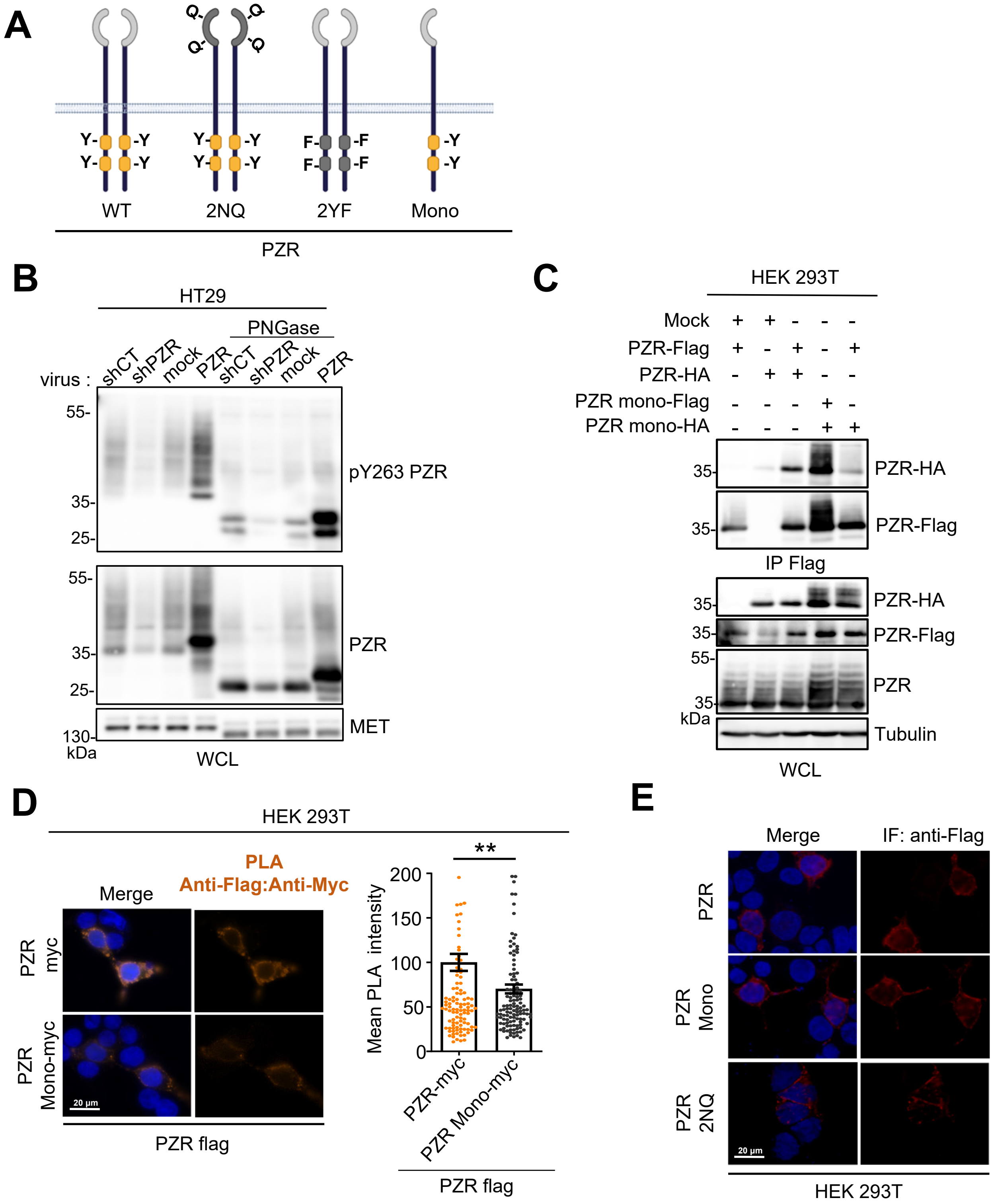

Figure S3: PZR self-association. **A:** PZR mutants used in this study. **B:** PZR N-Glycosylation in CRC cells. PZR expression in HT29 cells showed a high degree of N-glycosylation, as shown by SDS gel analysis (ladder PZR bands of 30-55 kDa), which was blunted by peptide N-glycanase (PNGase) treatment (sharp PZR band of 28 kDa). WB analysis of PZR entities from HT29 cells transduced with indicated constructs and treated with PNGase as indicated for 1h before analysis. **C:** PZR self-association in HEK293T cells that were transfected with indicated WT and monomeric mutant PZR constructs. WB analysis of the level of PZR-HA co-immunoprecipitated with PZR-FLAG constructs. **D:** PLA analysis of PZR self-association in HEK293T cells. Transfected cells were detected by GFP co-expression. Left: representative example; right: PLA quantification (PLA intensity) (mean ± SEM of 30 cells analyzed/assay; *n*=3; ***p*<0.01 Student's t-test). **E:** Representative immunofluorescence showing membrane localization of indicated PZR mutants transfected in HEK293T cells. MW (kDa) are indicated on WBs.

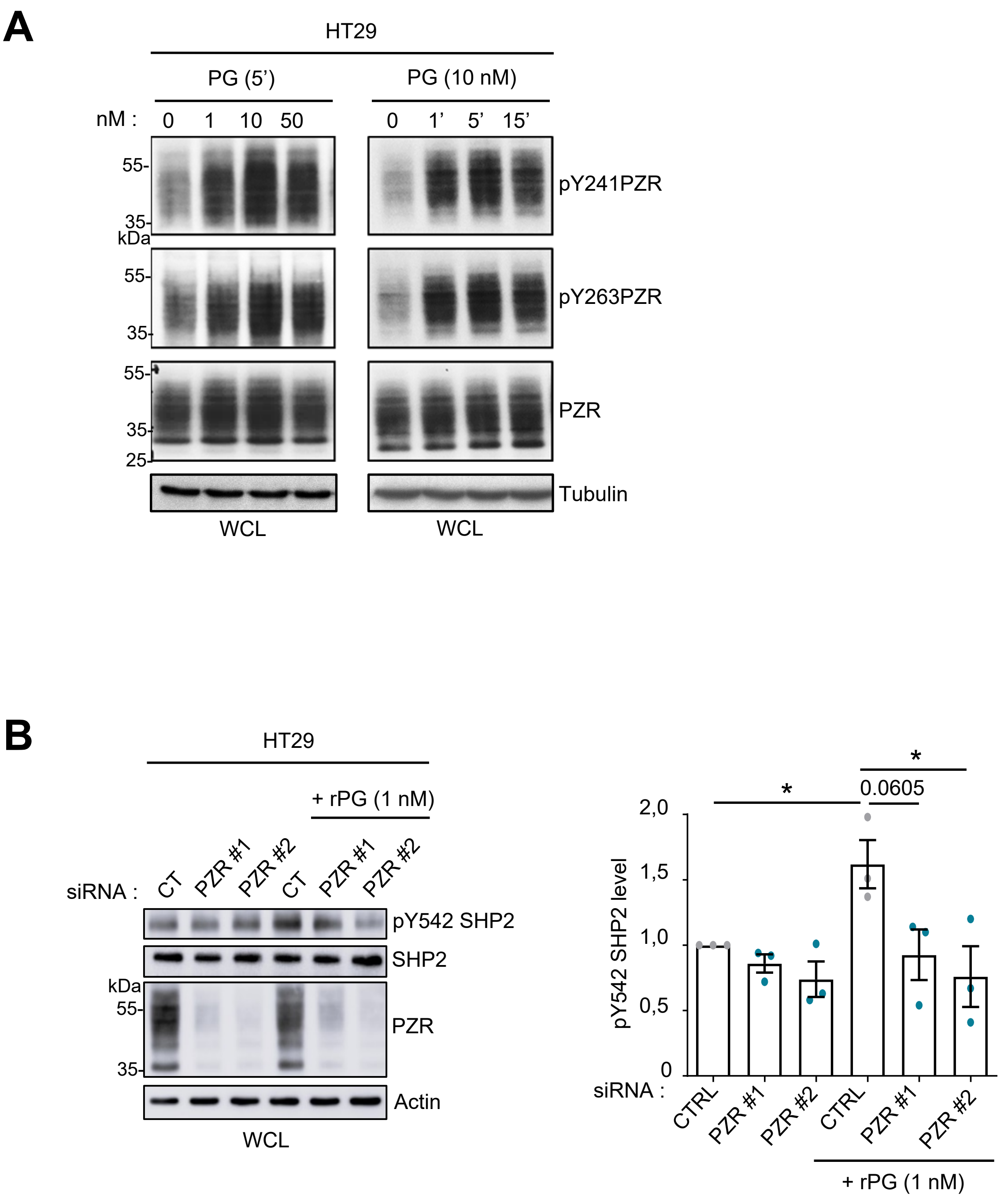

Figure S4: PZR-dependent PG phospho-signaling. A: Time course and dose-response effect of PZR phosphorylation by rPG in HT29 cells. **B**: PZR-dependent SHP2 activation by rPG. WB analysis of PZR, pPZR and pSHP2 levels in HT29 cells that were transfected with indicated siRNA and stimulated or not with rPG (5 min), as shown. Relative pSHP2 level as the mean ± SEM, n=3, p value close to 0.05 is shown *$p<0.05$; Student's t test. MW (kDa) are indicated on WBs.

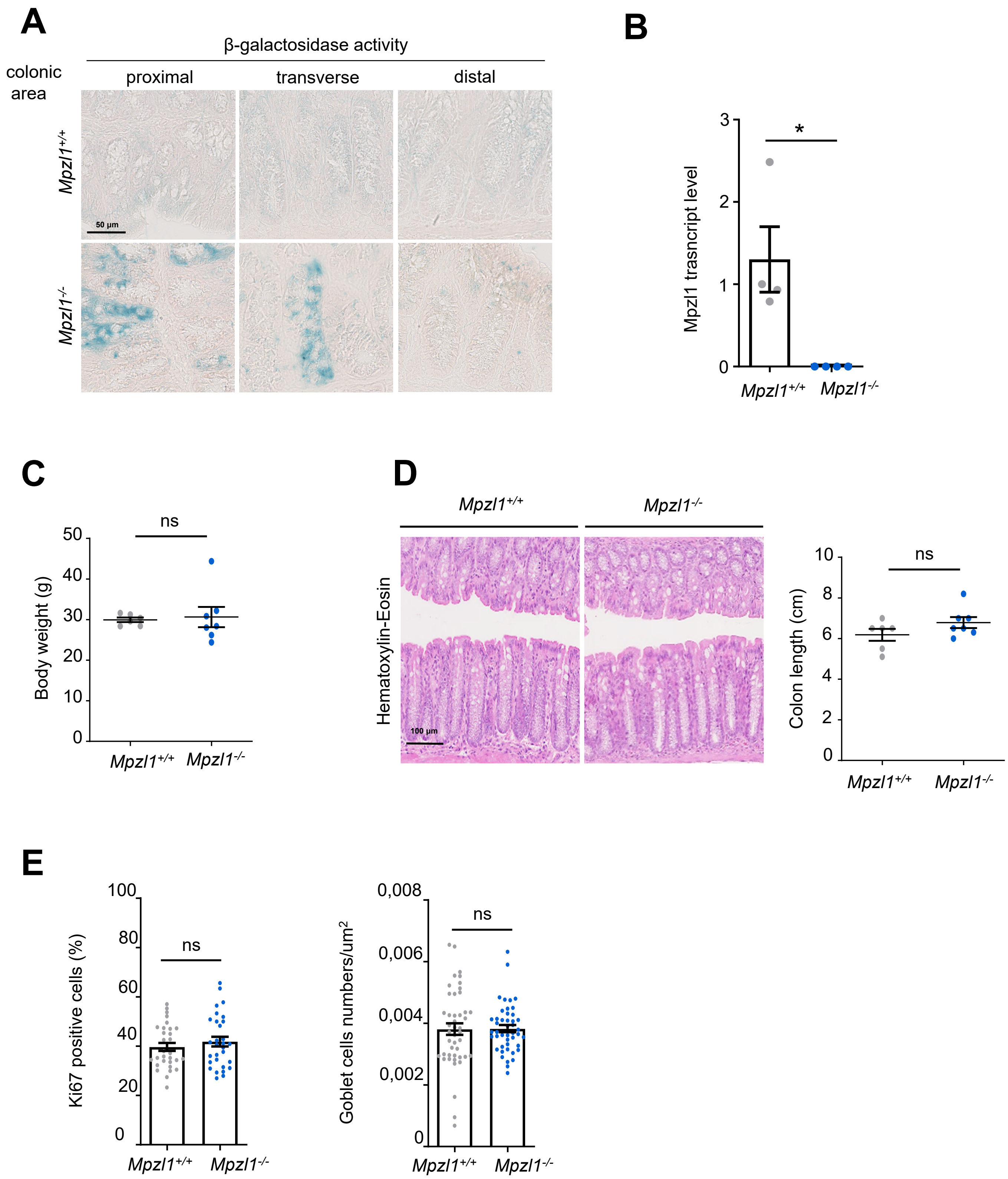

Figure S5: PZR gene (*Mpzl1*) inactivation does not affect intestinal homeostasis. A: *Mpzl1* colonic expression as assessed by beta-galactosidase activity in *Mpzl1-/-* mice. **B**: *Mpzl1* transcript levels in the intestine of *Mpzl1+/+* and *Mpzl1-/-* mice. **C-D**: No effect of *Mpzl1* inactivation on animal body weight or intestinal morphology (*n*=6-7 mice). **E**: *Mpzl1* inactivation does not affect colonic epithelial proliferation nor goblet cell number (mean ± SEM, 10 area/mouse *n*=3-5 mice; ns $p>0.05$; *$p<0.05$; Mann-Whitney).

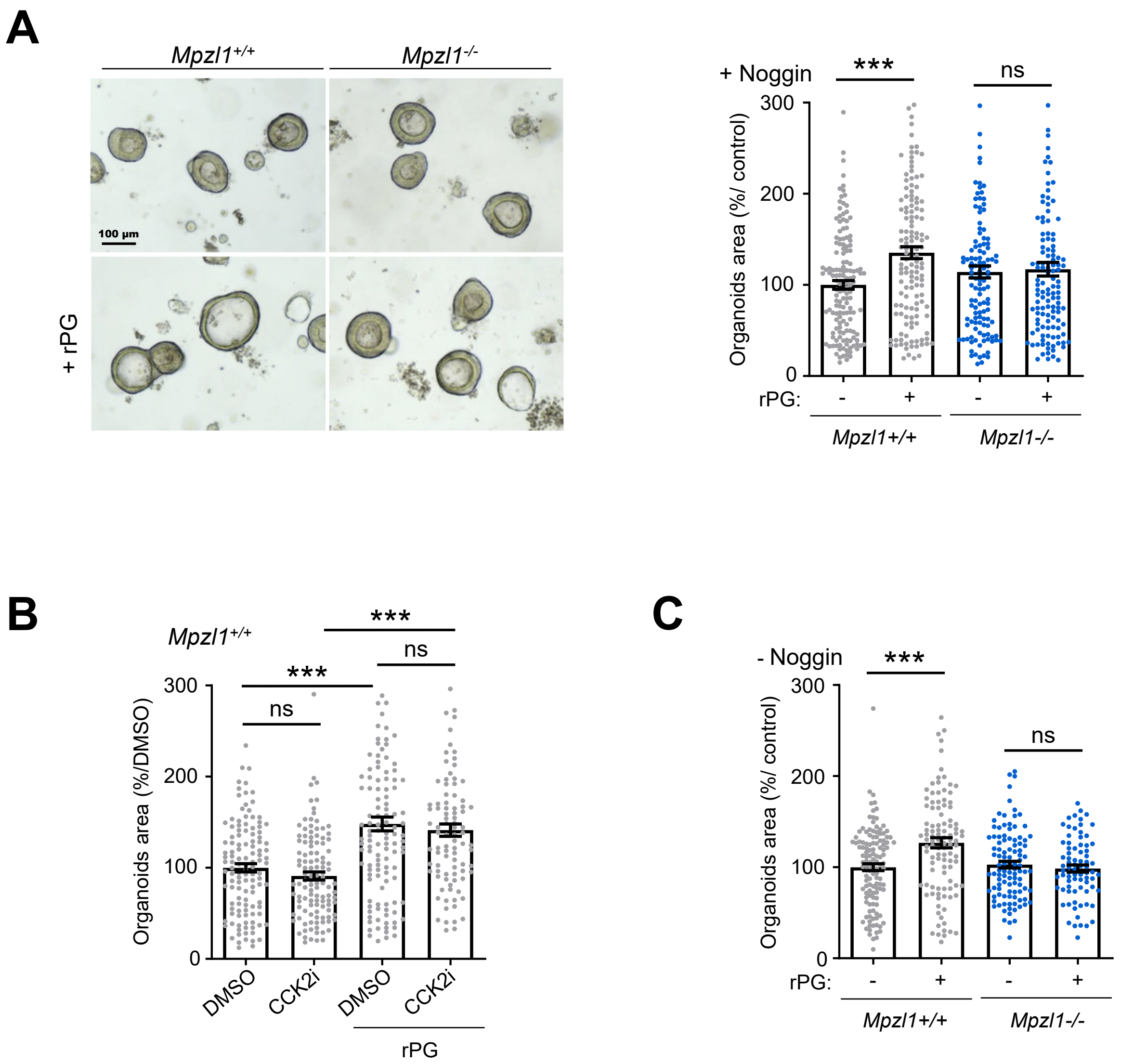

Figure S6: PZR mediates colon organoid development induced by rPG. A: Representative images showing the mitogenic effect of rPG (100 nM) on colon organoids derived from isolated crypts of wild-type (*Mpzl1+/+*) and *Mpzl1-/-* mice (left), and its quantification (right). **B:** The CCKBR inhibitor L-365,260 (1 µM) does affect the rPG-induced mitogenic effect. **C:** The colonic mitogenic effect of rPG is independent of Noggin. Data are presented as mean ± SEM, with 15–50 organoids analyzed per mouse (*n*=3 mice per group). Statistical significance was assessed using the Mann-Whitney test: ***$p < 0.001$; ns, not significant ($p > 0.05$).

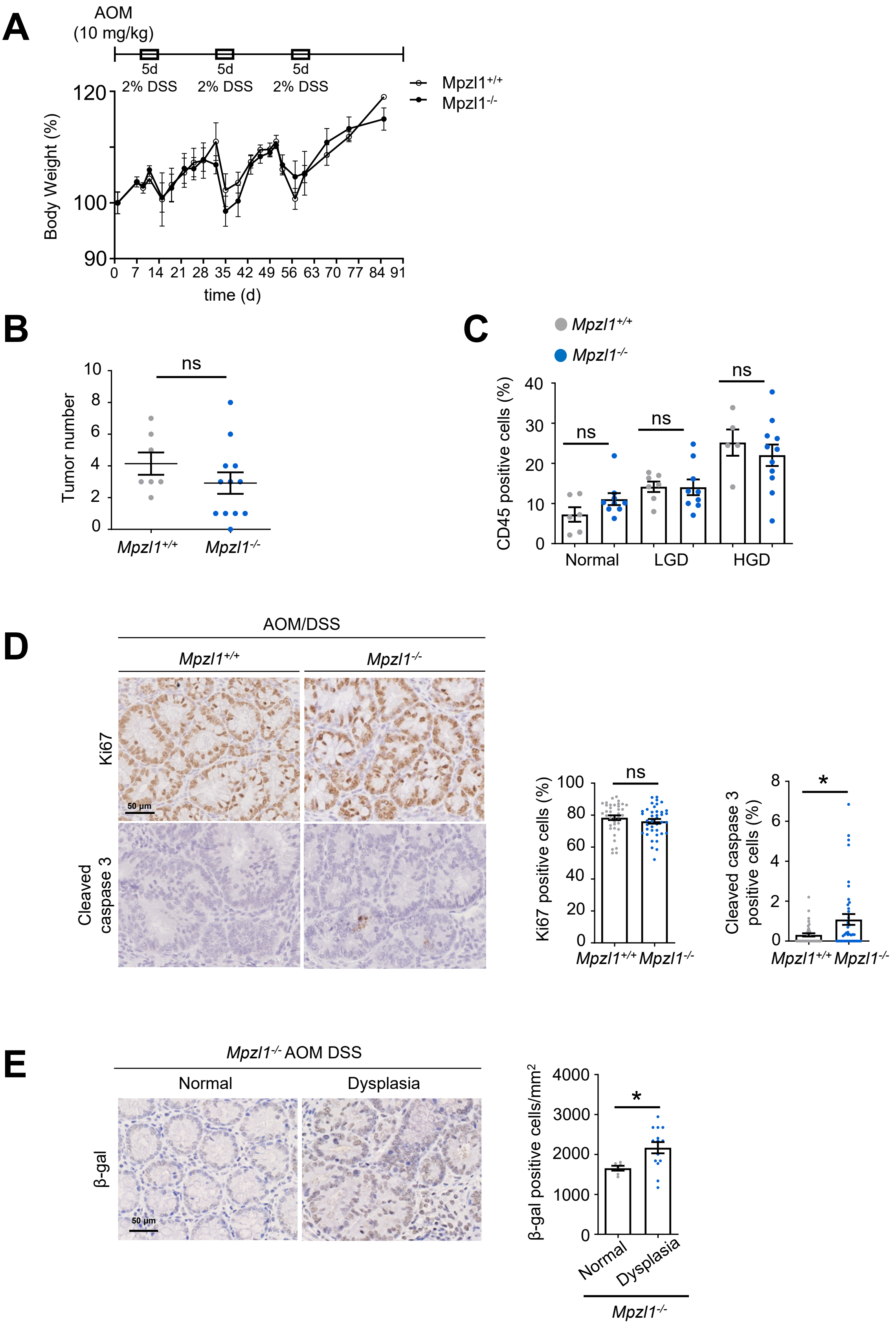

Figure S7: Mpzl1 inactivation reduces colonic transformation of AOM/DSS-treated mice. A: body-weight over time during AOM/DSS treatment (mean ± SEM, n=7-12 mice). **B**: tumor numbers. **C**: Immune infiltration (CD45 positive cells) in normal, low-grade (LGD) and high-grade (HGD) Dysplastic area. **D**: epithelial cell proliferation and apoptosis in the transformed colonic epithelium. **E**. *Mpzl1* promoter activation during colonic transformation (beta-galactosidase activity in AOM/DSS-treated *Mpzl1-/-* mice). Data are expressed as the mean ± SEM, *n*=3-6 mice ns *p*>0.05; **p*<0.05; Mann-Whitney test.

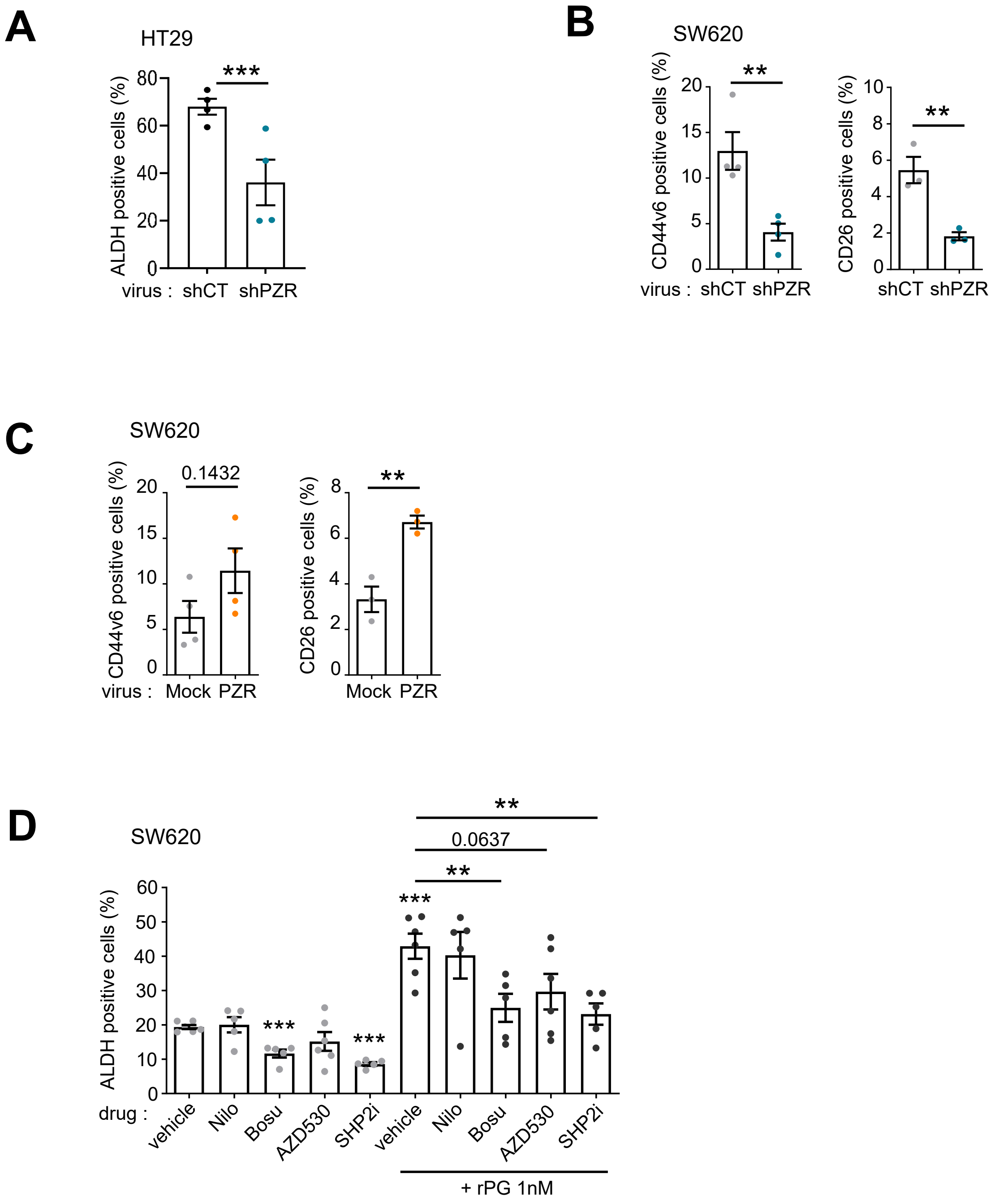

Figure S8: PZR mediates PG-dependent CSC-like properties of CRC cells. A: PZR depletion reduces ALDH activity in HT29 cells. **B:** PZR depletion reduces the cell surface level of CSC markers CD26 and CD44v6 in SW620 cells. **C:** PZR overexpression increases the cell surface levels of CSC markers CD26 and CD44v6 in SW620 cells. **D:** Inhibition of rPG-induced ALDH activity by SRC-like (bosutinib and AZD530 1 µM) and SHP2 inhibitors (SHP2i 1 µM). Is shown the mean ± SEM; *n*=3-6; *p* values close to 0.05 are indicated; ***p*<0.01; ****p*<0.001; Student's t-test.

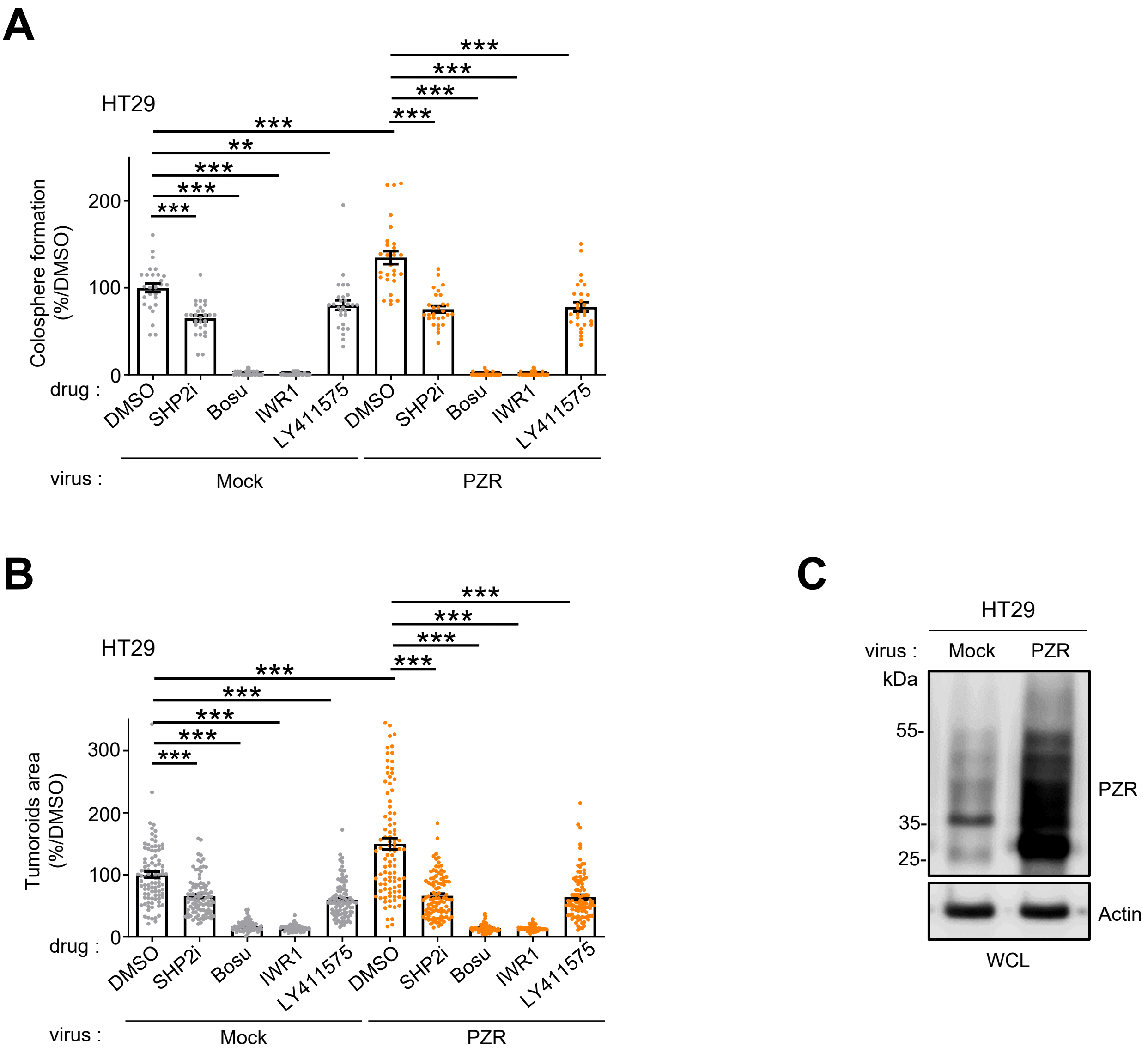

Figure S9: A SHP2/SRC/β-catenin/Notch signaling in PZR CSC-like effect in PG-high CRC cells. Effect of pharmacological inhibition of the PZR signaling components (i.e., SHP2i, SRC inhibitor bosutinib, and Notch inhibitor LY411575, 1 µM; Wnt inhibitor IWR1, 10 µM) on its CSC-like functions using PG-high HT29 cells. **A:** colonospheres (mean ± SEM of 10 replicates/condition, n=3 independent experiments; **p<0.01; ***p<0.001, Mann-Whitney test). **B**: tumoroids (mean ± SEM of 20-40 tumoroids analyzed *per* conditions, n=3; ***p<0.001, Mann-Whitney test). **C**: WB analysis of PZR overexpression. MW (kDa) are indicated on WBs.

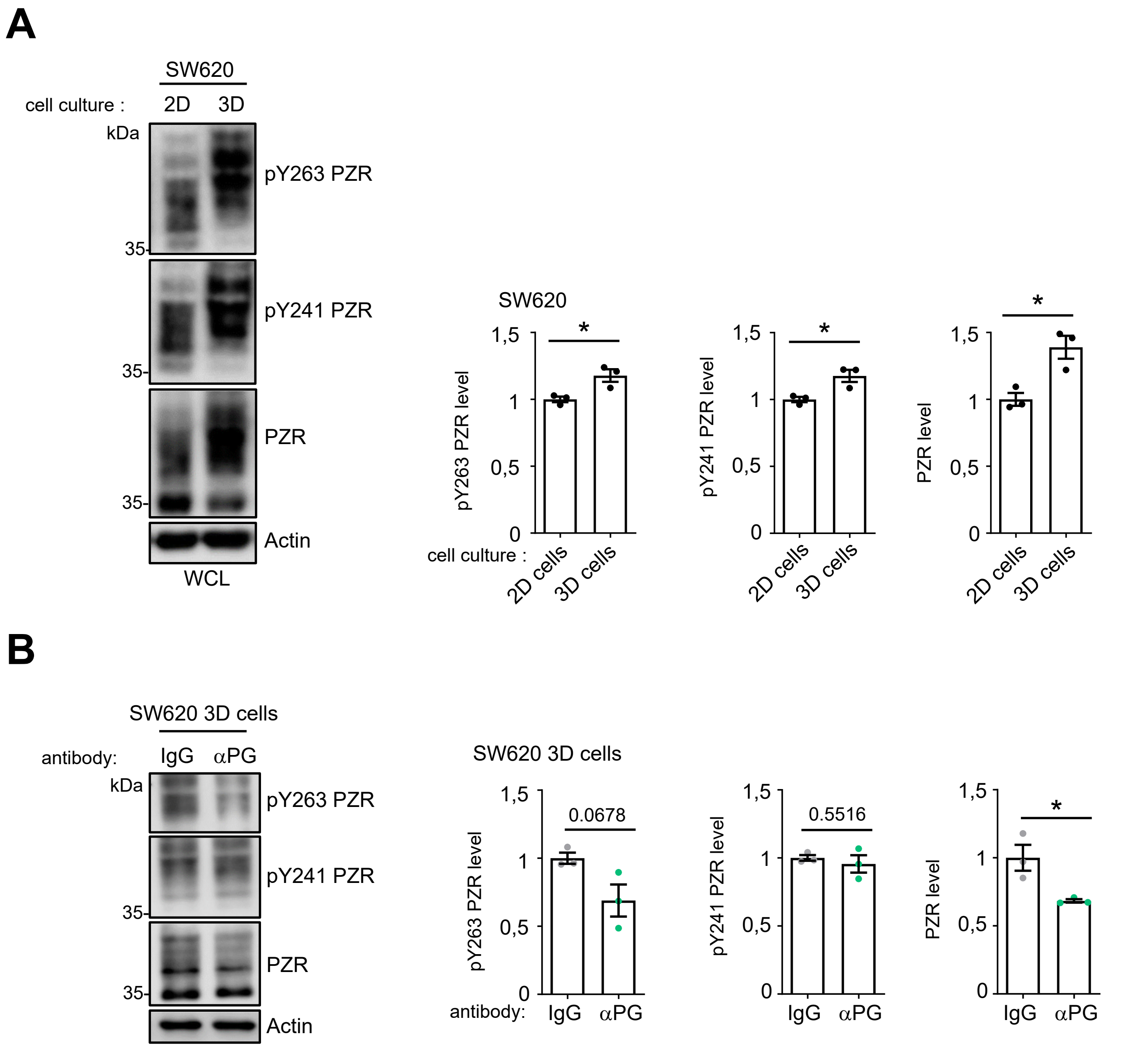

Figure S10: PZR activity is elevated in 3D colonospheres culture of SW620 cells. A: WB analysis of PZR and pPZR levels in cells grown as monolayers in serum-supplemented medium (2D) and as colonospheres (3D). **B**: quantification (fold control) as the mean ± SEM; *n*=3; **p*<0.05 Student's t-test. **C.** PG-dependent elevated pPZR levels in 3D. Secreted PG was inhibited by cells treatment with anti-PG antibodies (50 µg/ml). Is shown the quantification (fold control) as the mean ± SEM; *n*=3; **p*<0.05 Student's t-test. MW (kDa) are indicated.

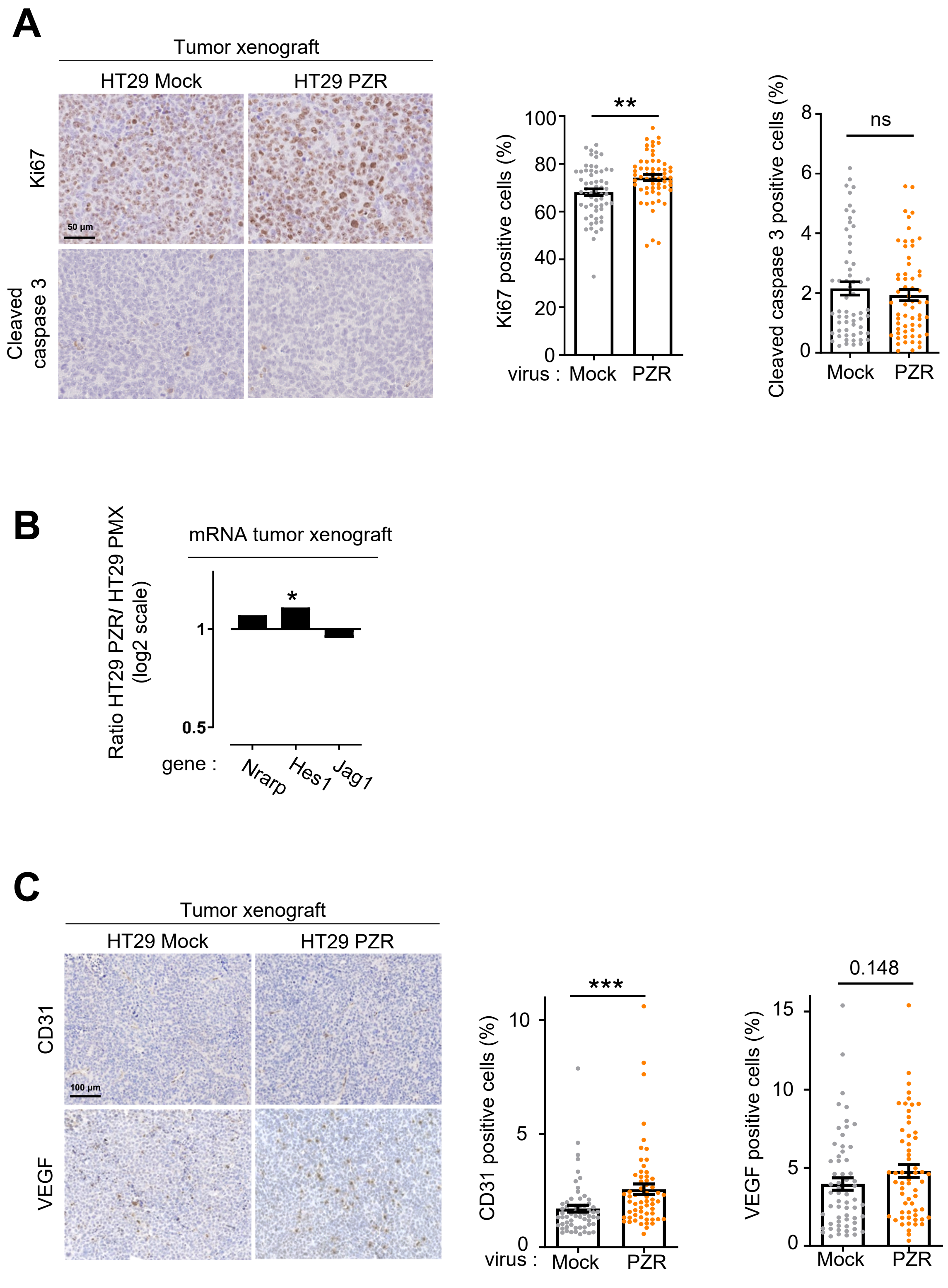
A
Tumor xenograft
HT29 Mock
HT29 PZR
Ki67
Cleaved caspase 3
50 µm
Ki67 positive cells (%)
100
80
60
40
20
0
**
virus : Mock PZR
Cleaved caspase 3 positive cells (%)
8
6
4
2
0
ns
Mock PZR
B
mRNA tumor xenograft
Ratio HT29 PZR/ HT29 PMX (log2 scale)
1
0.5
*
gene :
Nrarp
Hes1
Jag1
C
Tumor xenograft
HT29 Mock
HT29 PZR
CD31
VEGF
100 µm
CD31 positive cells (%)
10
5
0
***
virus : Mock PZR
VEGF positive cells (%)
15
10
5
0
0.148
Mock PZR

D

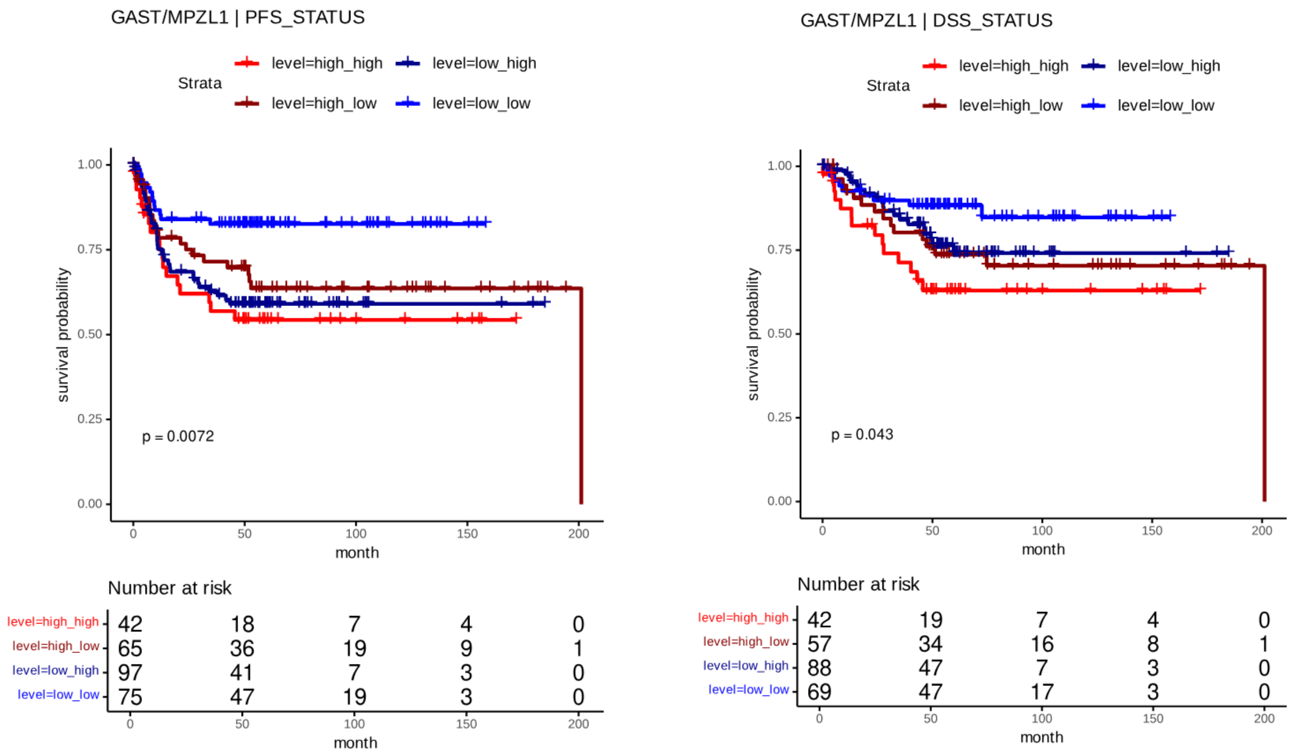

Figure S11: PZR overexpression increases tumor cell proliferation and angiogenesis in subcutaneous tumor of PG-high HT29 cells. A: IHC analysis of cell proliferation (Ki67) and apoptosis (cleaved Casapase-3). A representative example and the quantification (% of positive cells) from indicated tumors (mean ± SEM, 10 area/mouse, *n*=6 mice; ***p*<0.01, Mann Whitney test). **B:** PZR overexpression increases selected Notch target genes expression *in vivo*. qPCR analysis of indicated genes (mean ± SEM, *n*=4 mice per group; **p*<0.05, Mann Whitney test). **C:** IHC analysis of angiogenic markers. A representative example and the quantification (% of positive cells) from indicated tumors (mean ± SEM, 10 area/mouse, *n*=6 ; ns *p*>0.05; ****p*<0.001, Mann Whitney test). **D:** FPS and DSS probability in a cohort of primary CRC patients[57] depending on *GAST* and *MPZL1* transcripts levels, as indicated. The numbers of patients at risk are indicated.

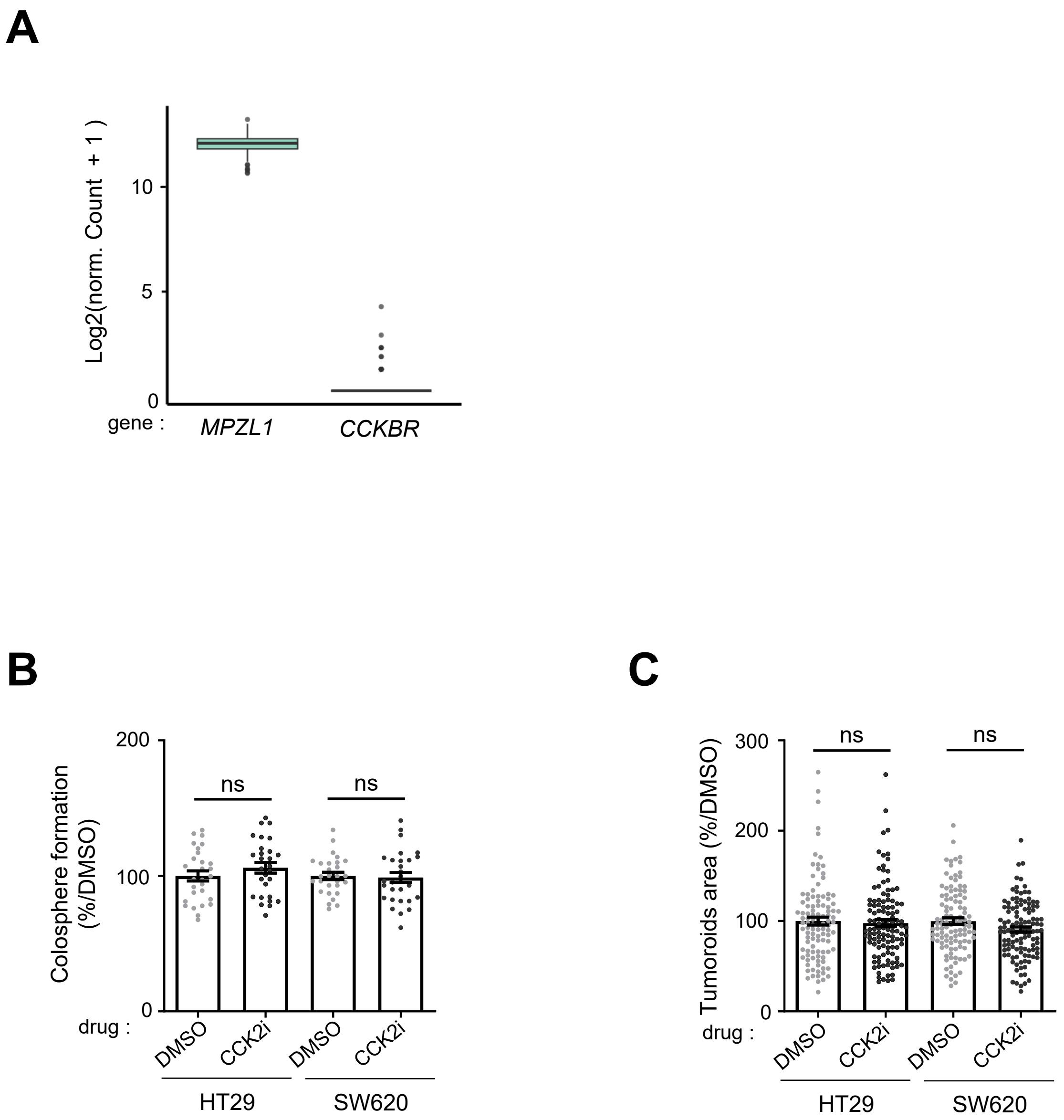

Figure S12: CCKBR does not play a major role in mediating PG's CSC-related effects in CRC cells. **A**: Log2 transcript level of *MPZL1* and *CCKBR* in the cohort of primary CRC patients described in Roelands et al (*Nat Med* 2023, 29, 127). **B and C:** Inhibition of CCKBR (L365,260, 1 µM) does not affect colonosphere (mean ± SEM of 9-10 replicates/condition, *n*=3 independent experiments; ns *p*>0.05; Mann-Whitney test) (panel B), nor tumoroid formation (mean ± SEM of 35-45 tumoroids analyzed per conditions, *n*=3; ns, *p*>0.05; Mann Whitney test) (panel C).

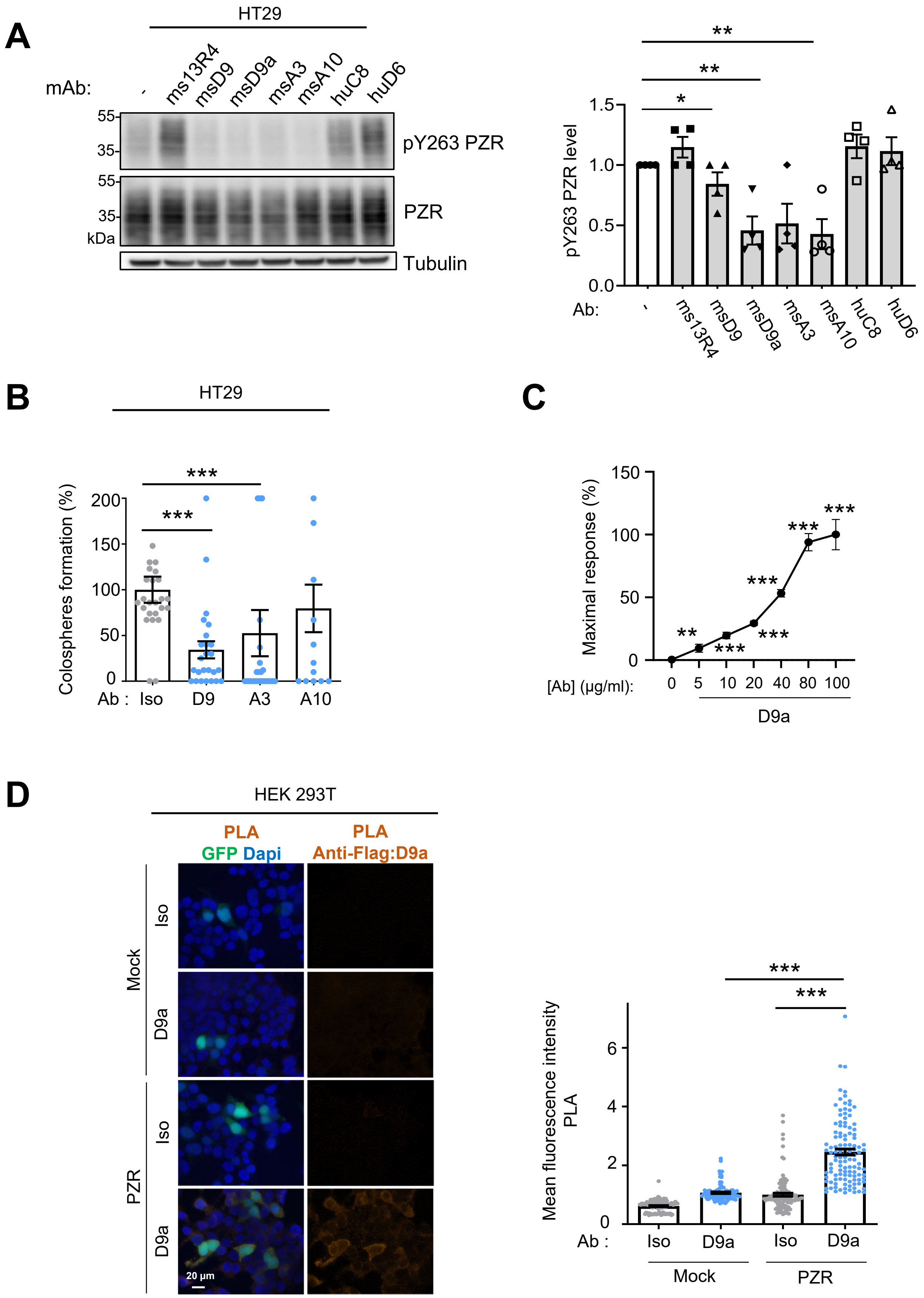
A
HT29
mAb:
-
ms13R4
msD9
msD9a
msA3
msA10
huC8
huD6
55
35
pY263 PZR
55
35
kDa
PZR
Tubulin
**
**
*
1.5
1.0
0.5
0.0
pY263 PZR level
Ab:
B
HT29
***
***
200
150
100
50
0
Colospheres formation (%)
Ab :
Iso
D9
A3
A10
C
150
100
50
0
Maximal response (%)
**
***
***
***
***
***
[Ab] (µg/ml):
0
5
10
20
40
80
100
D9a
D
HEK 293T
PLA
GFP Dapi
PLA
Anti-Flag:D9a
Mock
Iso
D9a
PZR
Iso
D9a
20 µm
***
***
6
4
2
0
Mean fluorescence intensity
PLA
Ab :
Iso
D9a
Iso
D9a
Mock
PZR

E

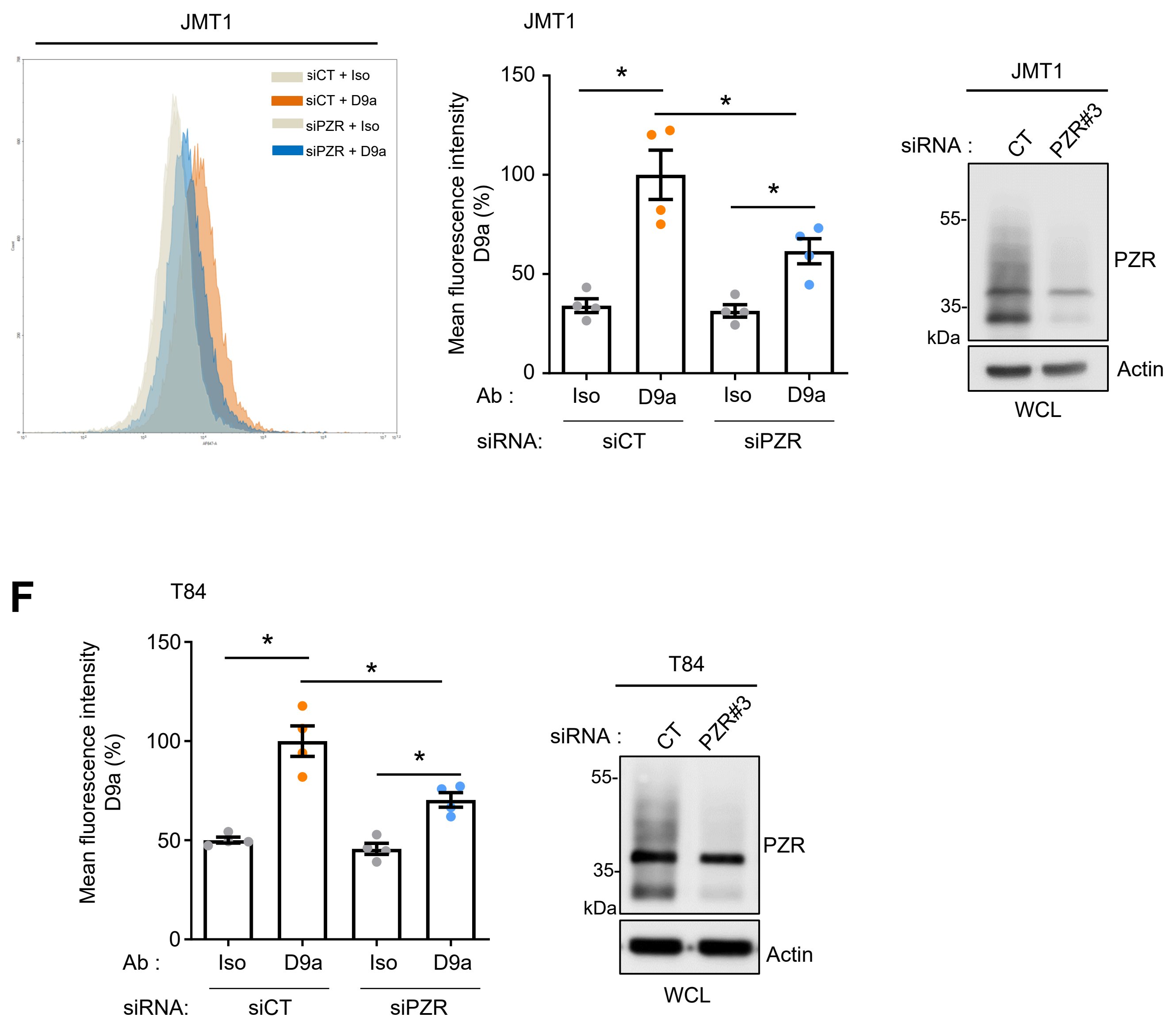

F

Figure S13: Characterization of selected PZR mAbs. A: WB analysis of the effect of the indicated mAb (cell treatment overnight at 10 µg/ml) on pPZR levels in HT29 cells. A representative example and quantification (% controls) as mean ± SEM; n=4; *p<0.05; **p<0.01; Student's t-test. **B:** Effect of the indicated PZR mAb on HT29 colonosphere formation (mean ± SEM >4 replicate/condition, n=3, ***p<0.001, Mann-Whitney test). **C:** ELISA with D9a on coated PZR-EXT-His using EGFR-EXT-His as negative control (% maximal intensity, mean ± SEM, n=3; **p<0.01; ***p<0.001, Student's t-test). **D:** PLA analysis of D9a on HEK293T cells transfected with PZR-FLAG constructs. Transfected cells were detected by GFP co-expression. A representative example and PLA quantification (mean fluorescence intensity) (mean ± SEM of >25 GFP+ cells analyzed per condition from 3 independent experiments; **p<0.01; ***p<0.001; Student's t-test). **E:** FACS analysis of D9a (or control Iso; 50 µg/ml) on JMT1 breast cancer cells transfected with the indicated siRNA. A representative example and its quantification (mean ± SEM, n=4 *p<0.05, Mann Whitney test). WB analysis of PZR levels (right panel). **F:** FACS analysis of D9a on T84 CRC cells. MW are shown (kDa).

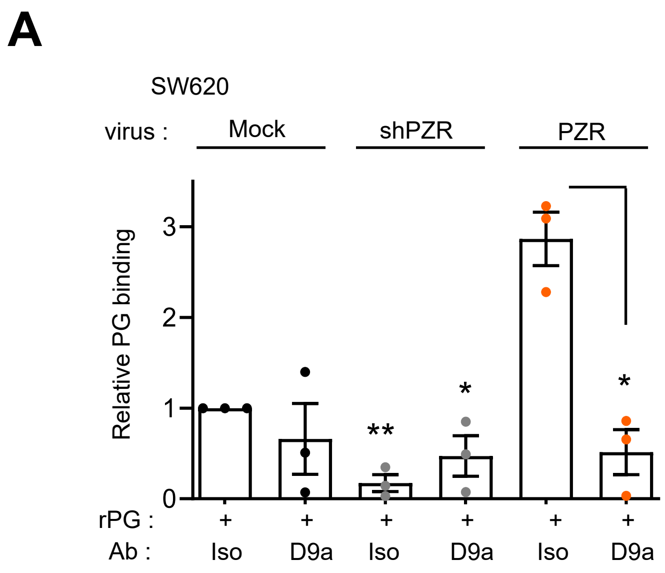

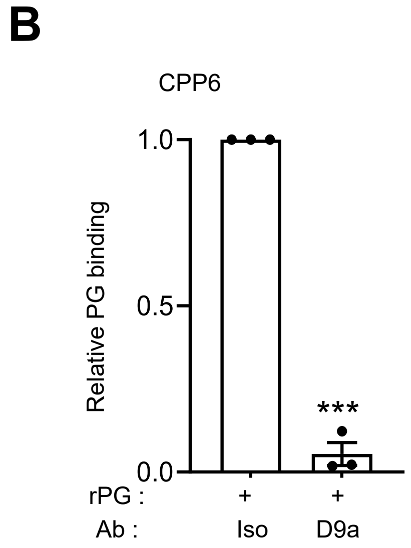

Figure S14: PZR mAb D9a reduces rPG binding to CRC cells. A: SW620 cells were pretreated 30 min with D9a (10 µg/ml) or Iso as a control before performing rPG cell binding using the protocol and cell lines described in Figure 1. **B:** CPP6 cells were pretreated overnight with D9a (10 µg/ml) or Iso as a control before performing rPG cell binding. Quantification (relative fluorescence intensity per cell expressed as mean ± SEM, n=3; *p<0.05; **p<0.01; ***p<0.001; Student's t-test).

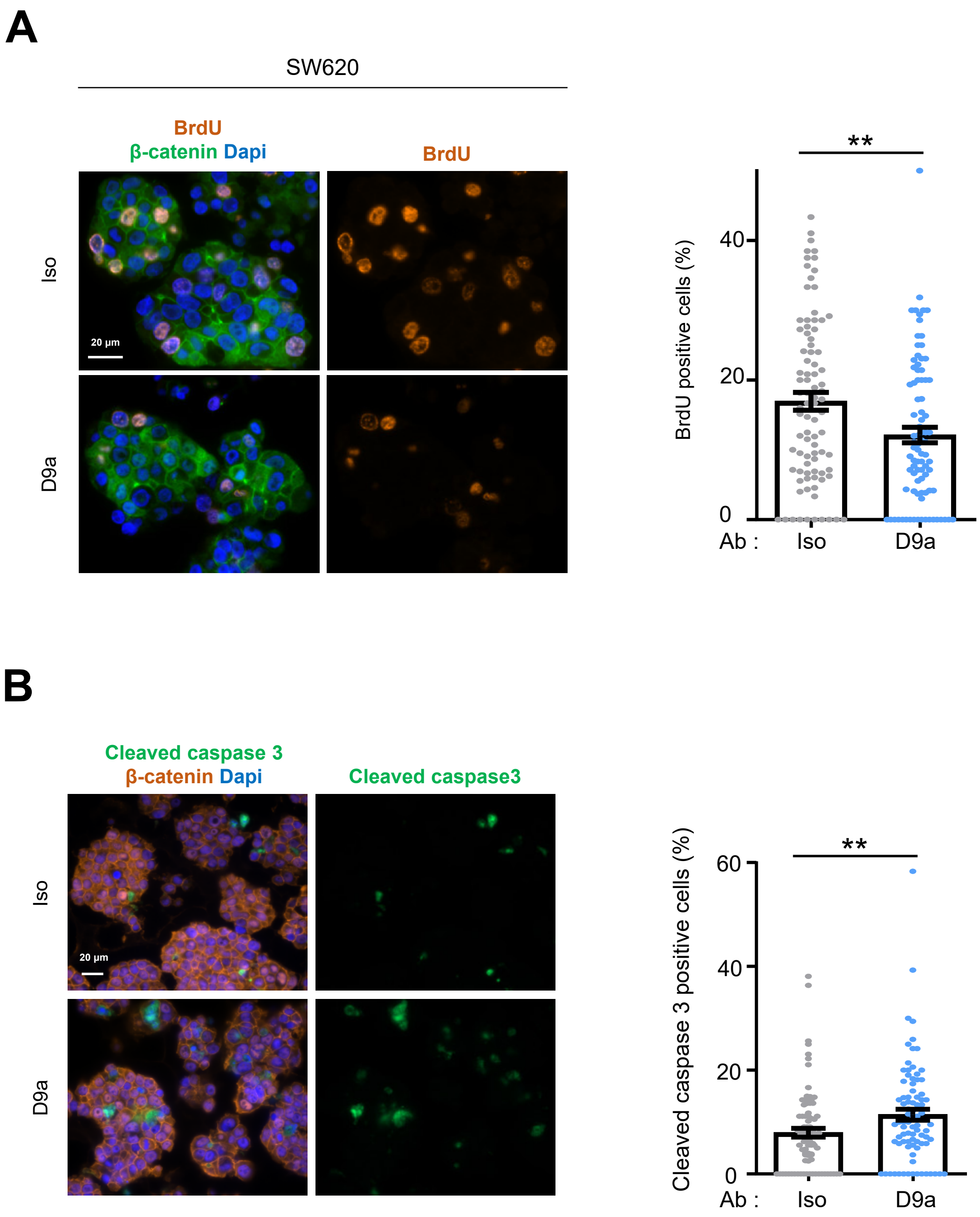

Figure S15: PZR mAb D9a reduces DNA synthesis and increases apoptosis in tumoroids derived from SW620 cells. A and B: Representative example (left) and quantification (right) of DNA synthesis (BrdU) (**A**) and apoptosis (cleaved-Caspase 3) (**B**) from SW620 cells treated with D9a (or control Iso, 10 µg/ml). Is shown the mean ± SEM of >25 tumoroids analyzed from n=3; ns, p>0.05; **p<0.01 ***p<0.001; Mann Whitney test).

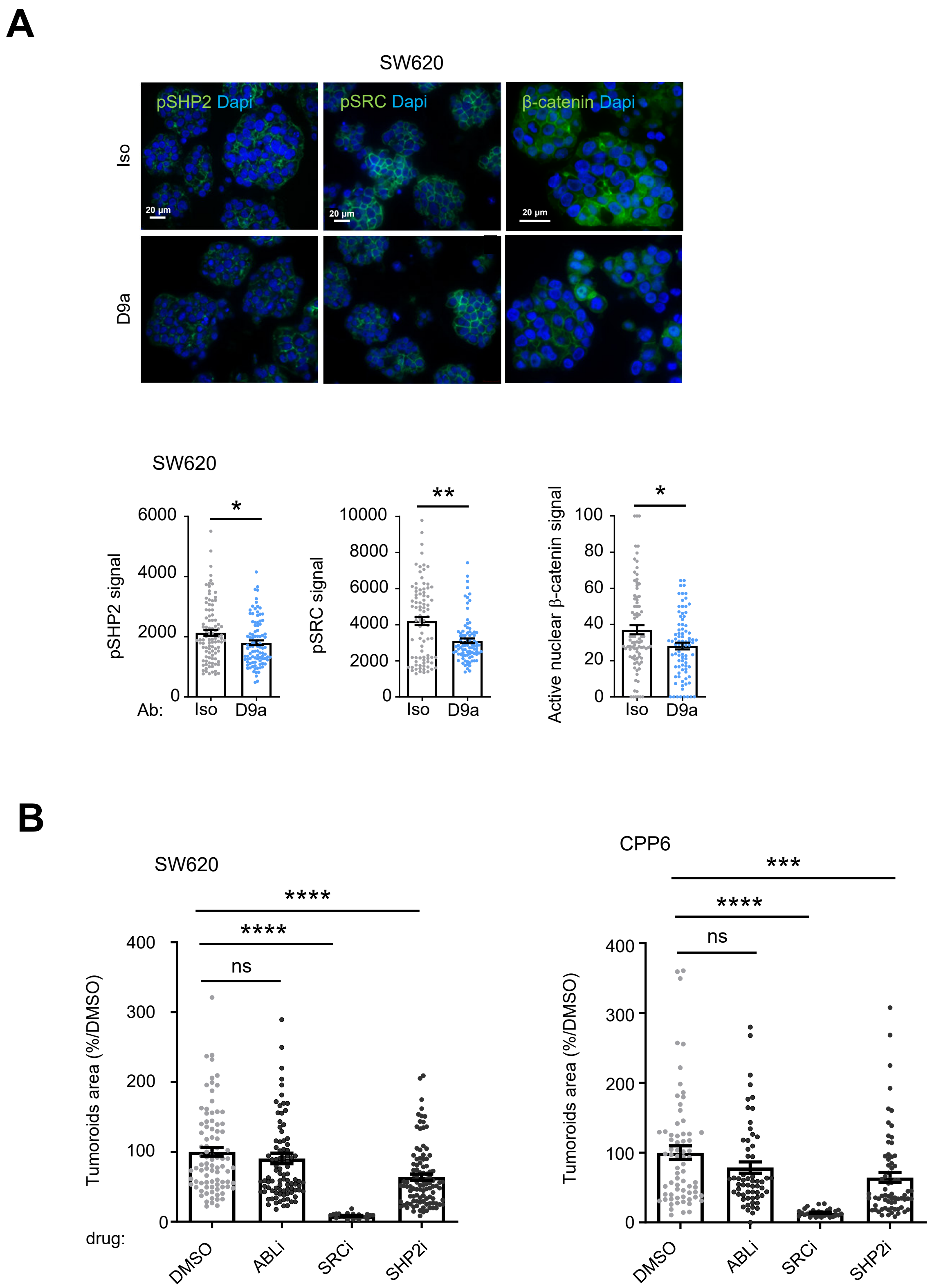

Figure S16: PZR mAb D9a reduces pSHP2/pSRC/beta-catenin signaling in tumoroids derived from SW620 cells. **A:** A representative example of immunofluorescence and quantification of pSHP2, pSRC and active nuclear beta-catenin in tumoroids treated with indicated mAb. **B:** tumoroids development is inhibited by SRCi (bosutinib) and SHP2i, but not ABLi (nilotinib). Is shown the mean ± SEM of about 25 tumoroids analyzed per condition; n=3; *p<0.05; **p<0.001; Mann Whitney test).

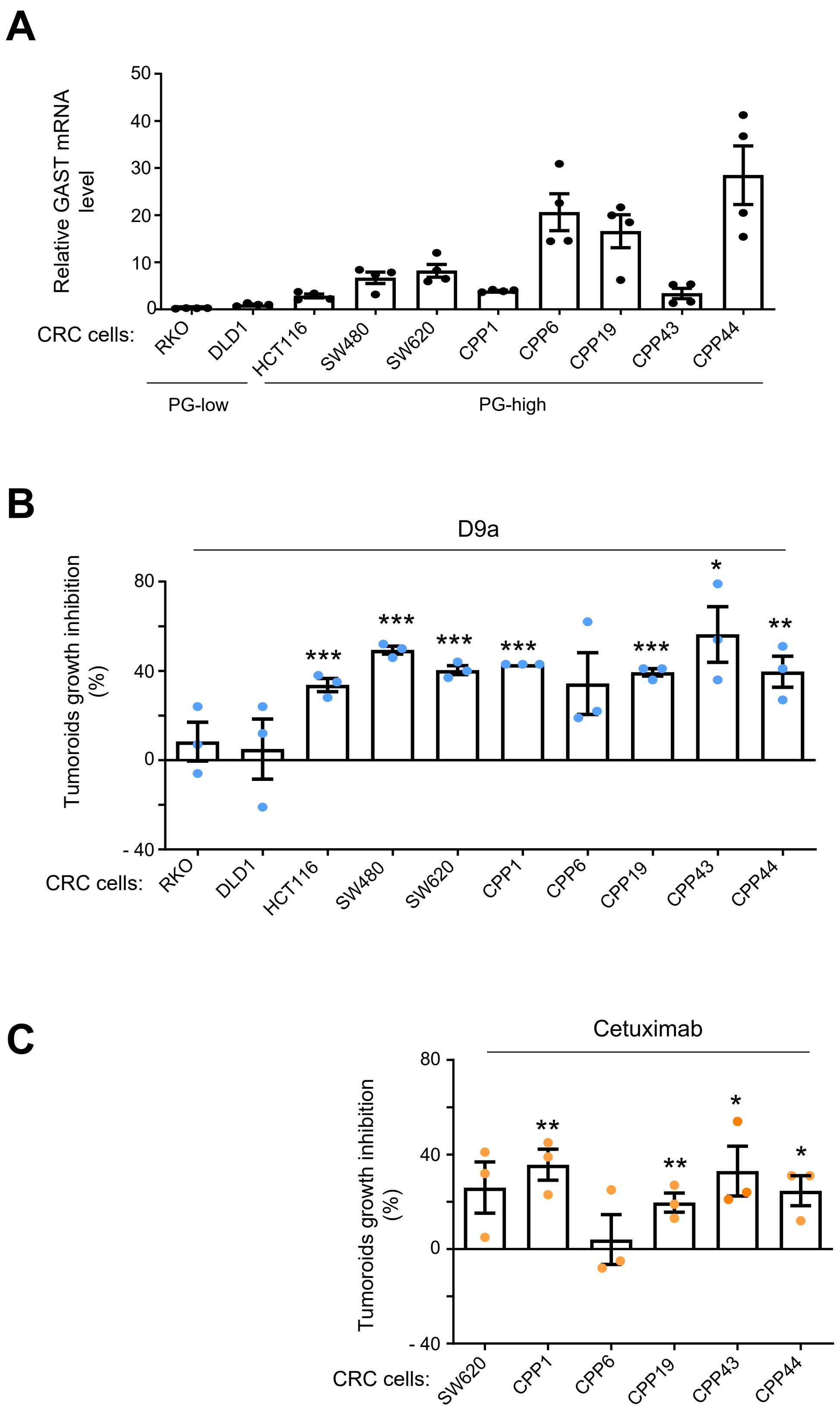

Figure S17: correlation between *GAST* expression in CRC colonospheres and D9a inhibition on tumoroids. A: *GAST* mRNA quantification in colonospheres of indicated CRC cells (relative to the level obtained in PG-low RKO cells, PG-high CRC cells: > 20 fold). **B:** D9a (10 µg/ml) inhibition (%). **C**: cetuximab (10 µg/ml) inhibition. Mean ± SEM; *n*=3; *p<0.05; **p<0.01; ***p<0.001 Student's t test.

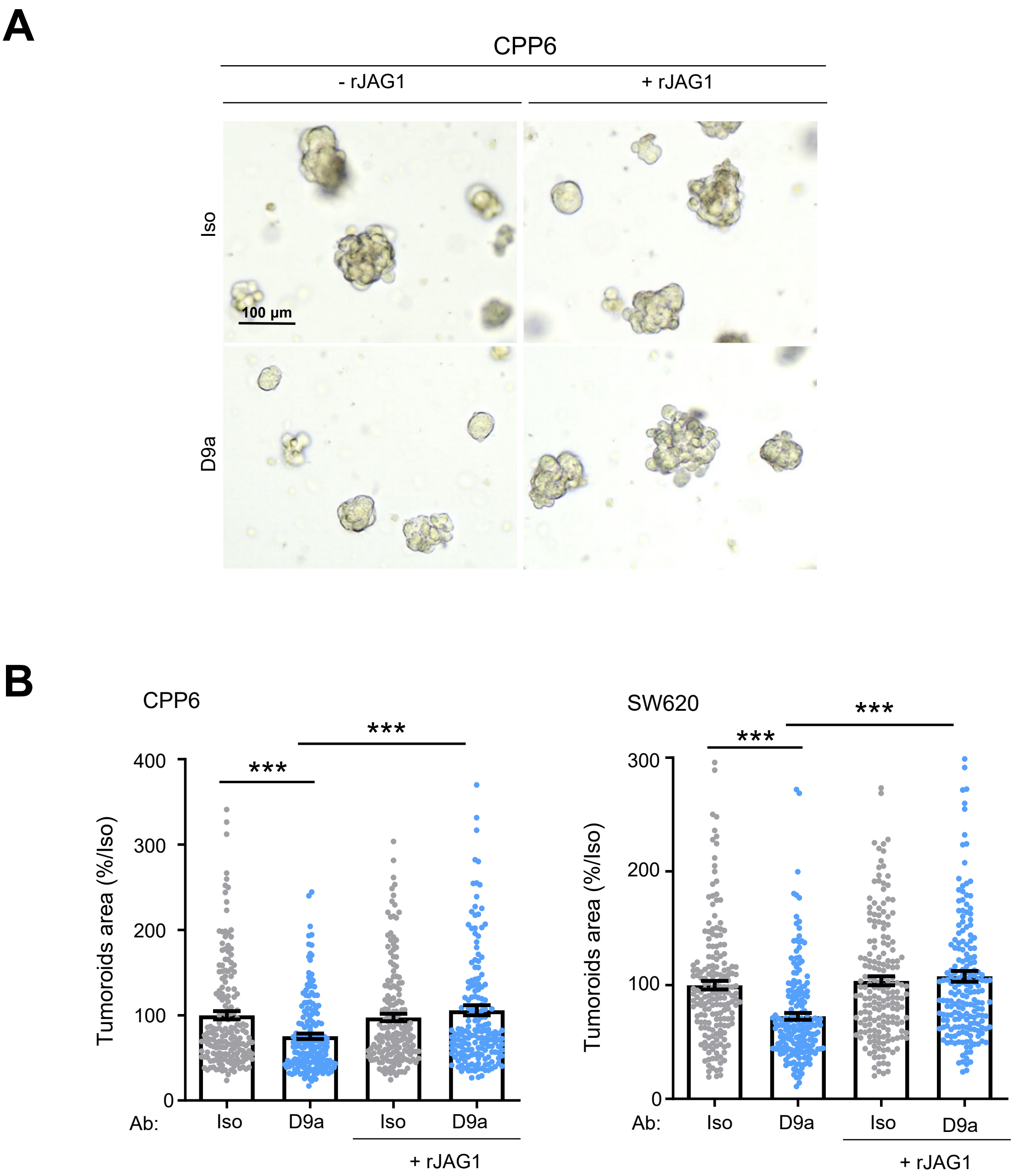

Figure S18: D9a inhibition on tumoroids is overcome by rJAG1 (10 µg/ml). A and B: Representative images (**A**) and quantification (**B**) of tumoroid size derived from the indicated PG-high CRC cells (mean ± SEM of >50 tumoroids analyzed per condition (*n*=3). Mann-Whitney test; ****p*< 0.001.

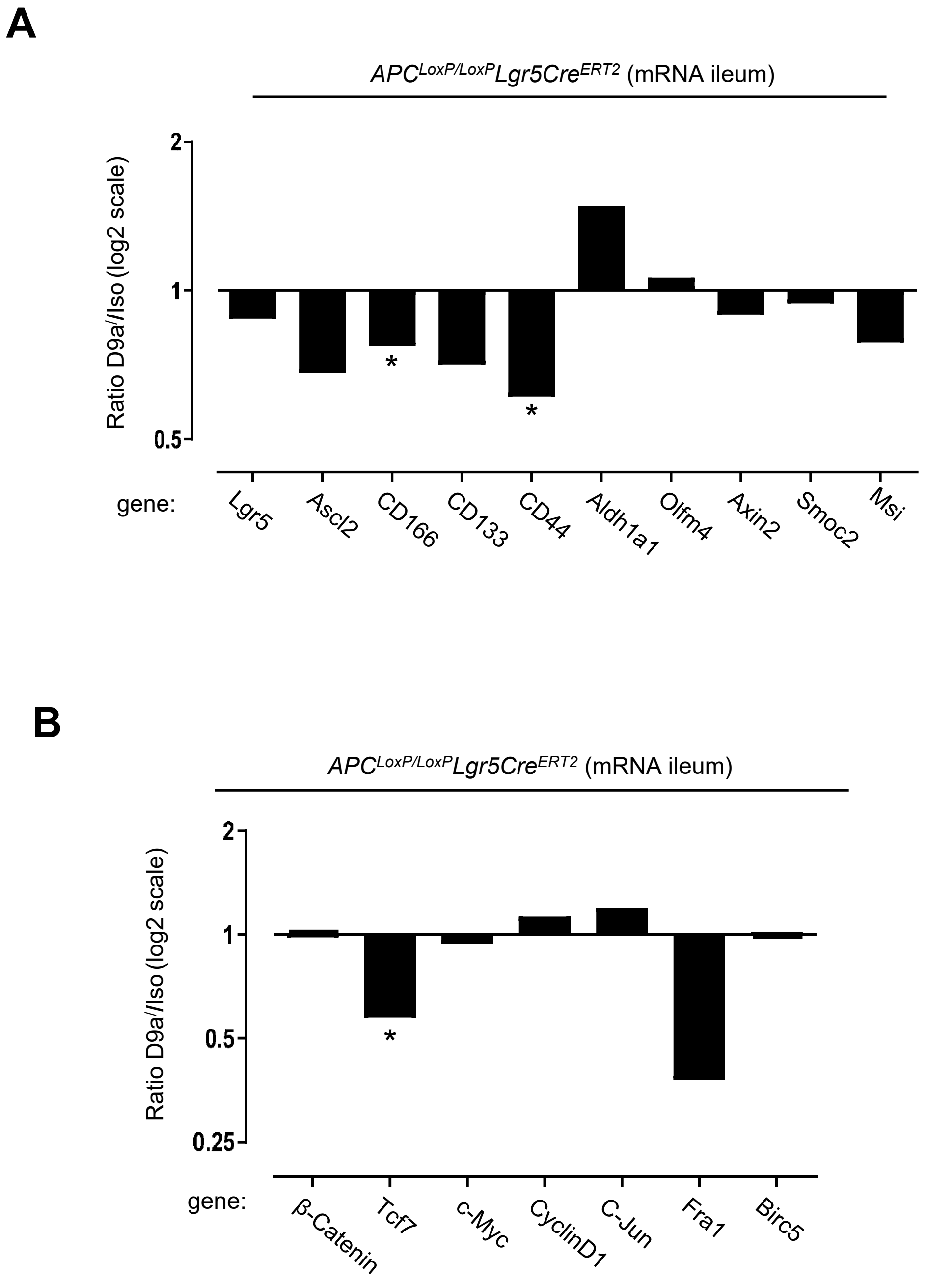

Figure S19: *In vivo* effect of D9a on selected CSC-dependent (A) and beta-catenin-dependent transforming (B) genes in the ileum of Apc$^{LoxP/LoxP}$Lgr5Cre^{ERT2}IRES-eGFP mice treated with tamoxifen. Is shown the transcript log2 ratio of indicated genes between Iso and D9a treated mice (mean ± SEM, *n*=4 mice per group; *p<0.05; Man Whitney test).

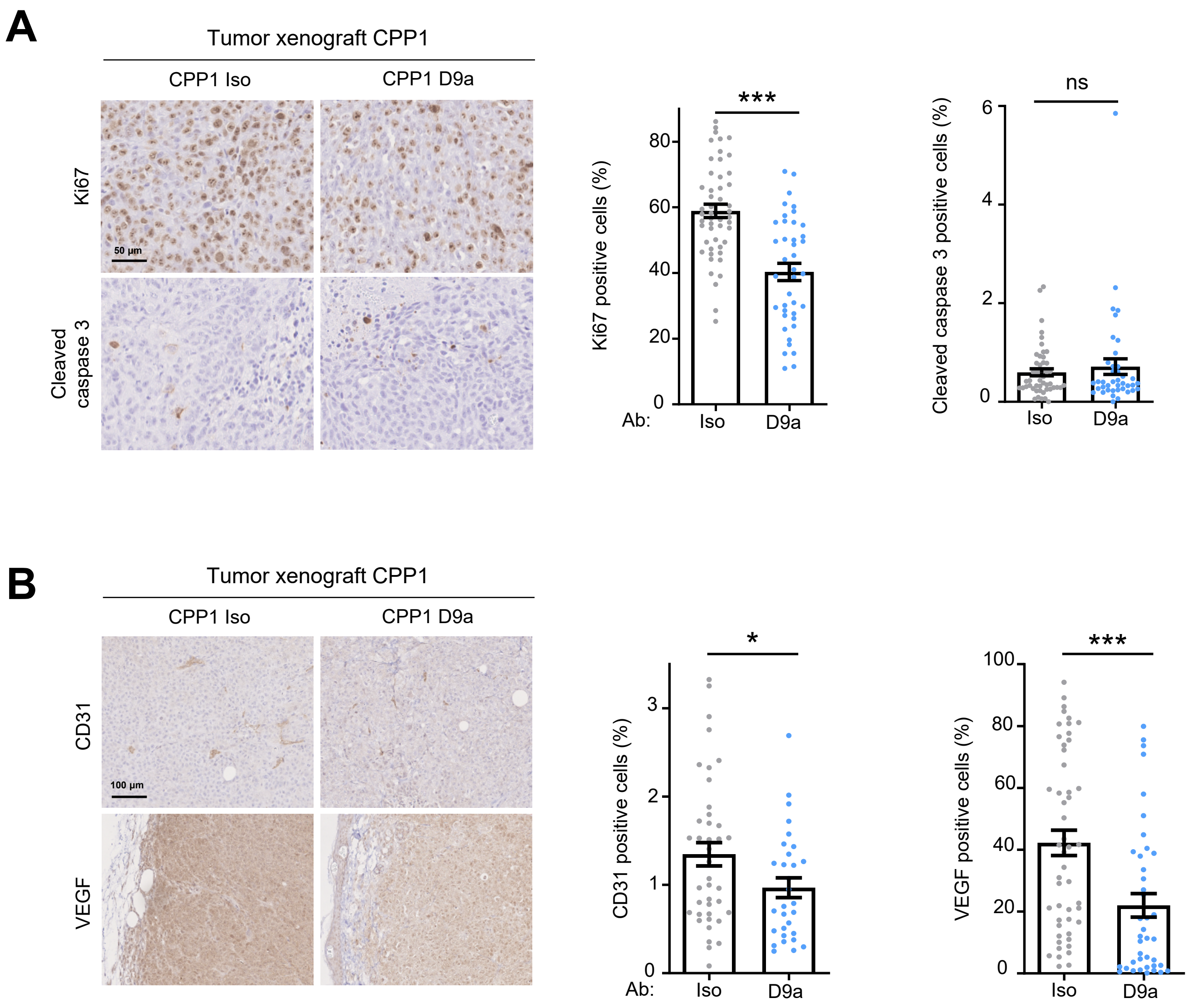

Figure S20: D9a reduces tumor cell proliferation and angiogenesis of CPP1 tumor xenograft. A: IHC analysis of tumor cell proliferation and apoptosis of CPP1 tumors. **B:** IHC analysis of indicated angiogenic markers in CPP1 tumors. A representative example and quantification (% positive cells) of CPP1 tumors (mean ± SEM of 10 area/mouse, n=4-5 mice per group; ns: p>0.05; *p<0.05; ***p<0.001 Mann Whitney test).

Figure 1 A, D and E

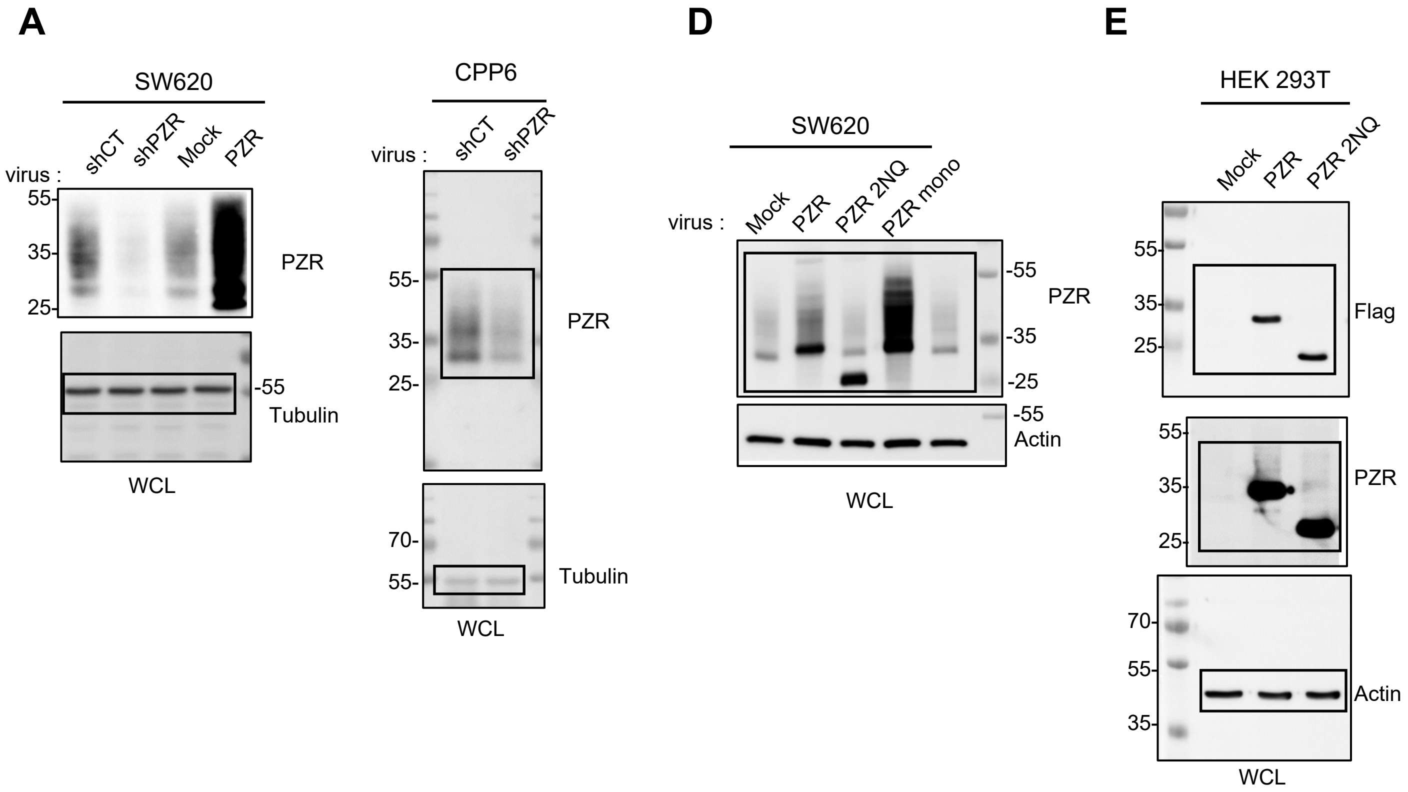

Figure 2A and B

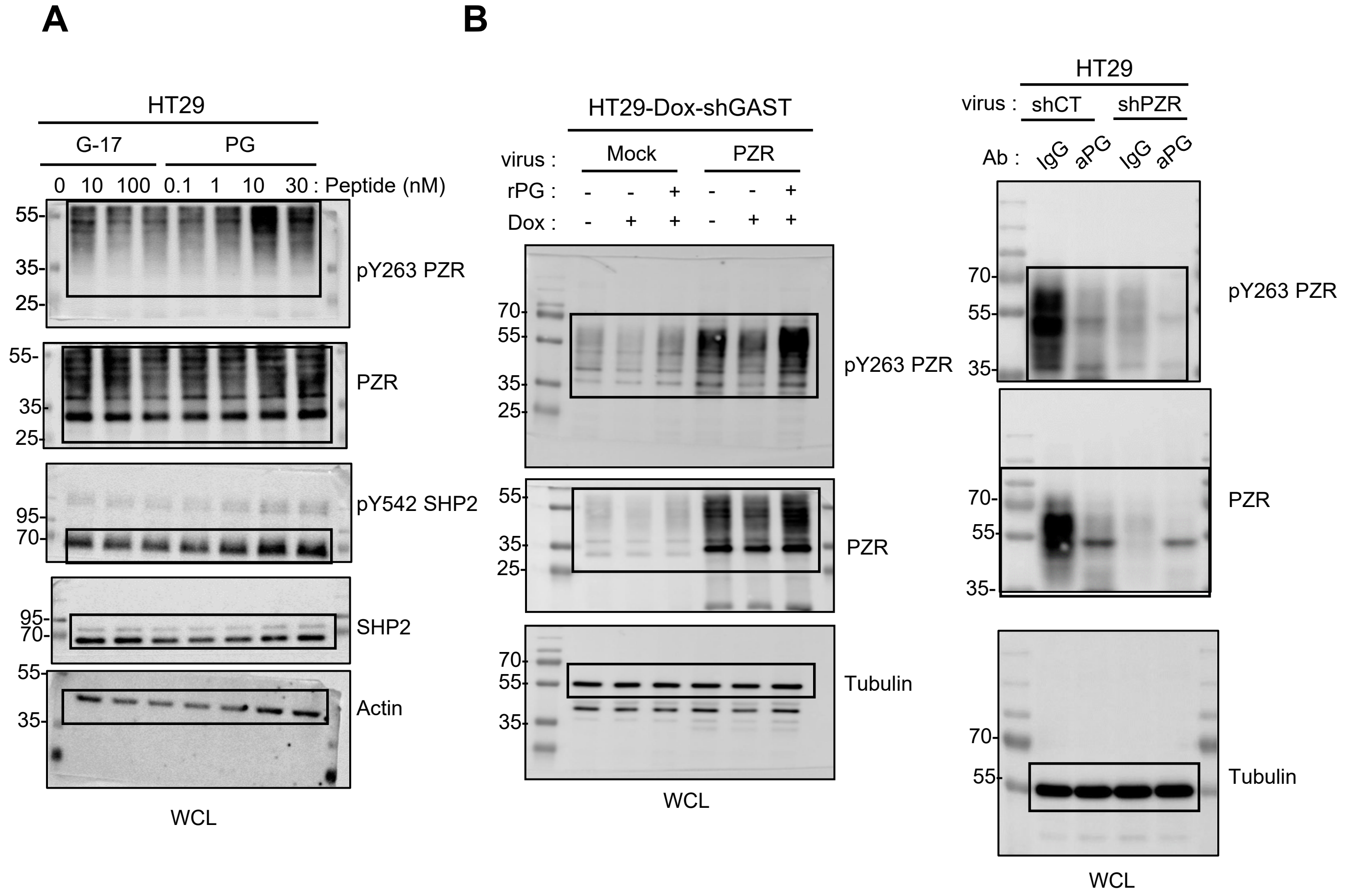

Figure S21: raw blots of Figures 1, 2, 4 and Figures S1, S4, S9, S10 and S13.

Figure 2C

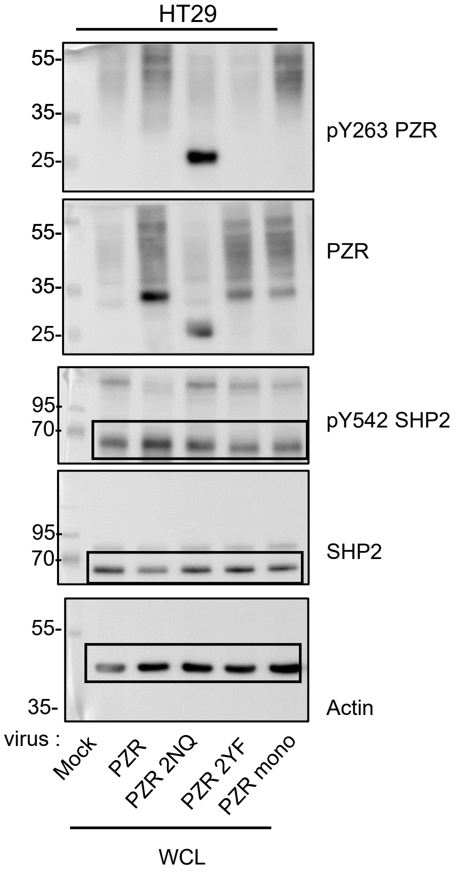

Figure 4D, E and F

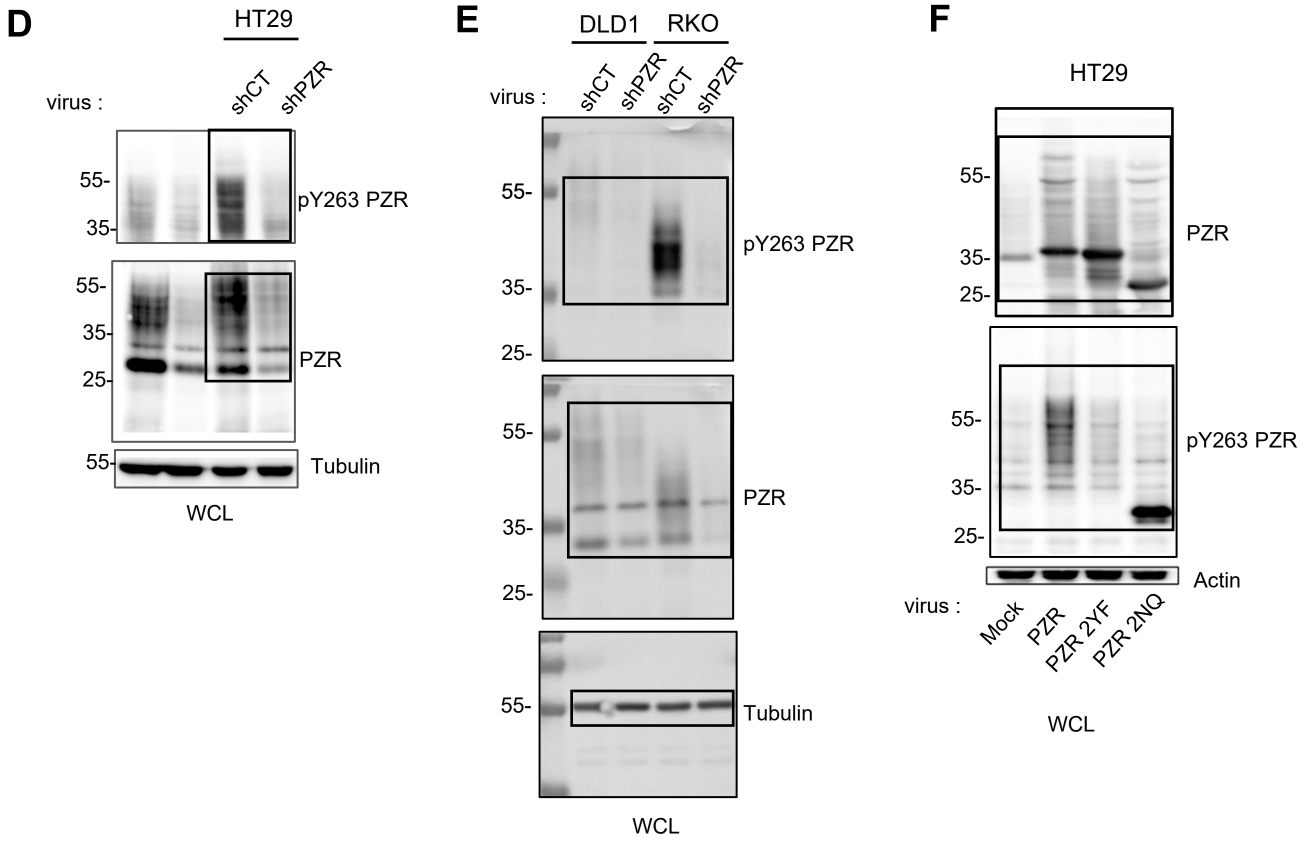

Figure S1A and B

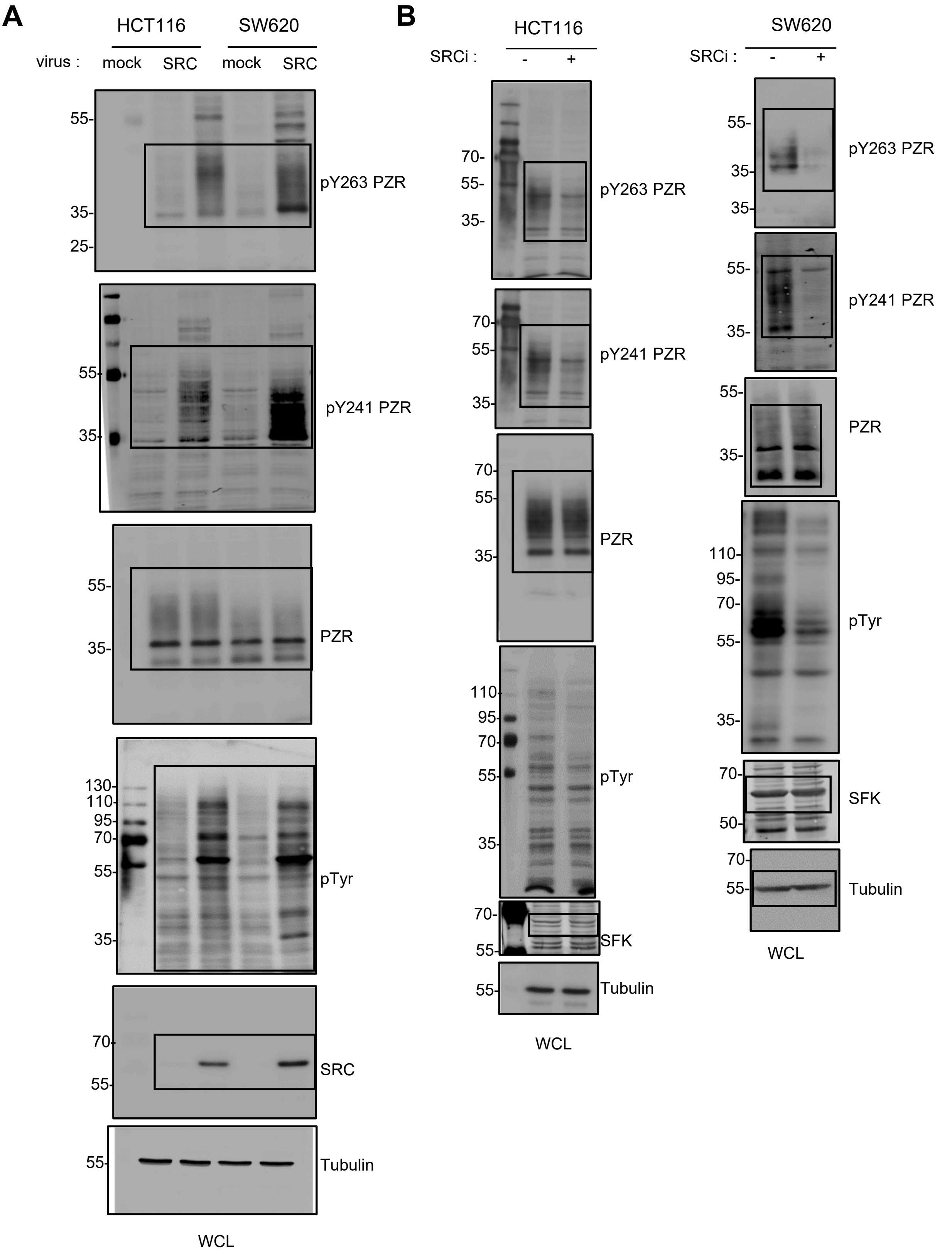

Figure S3B and C

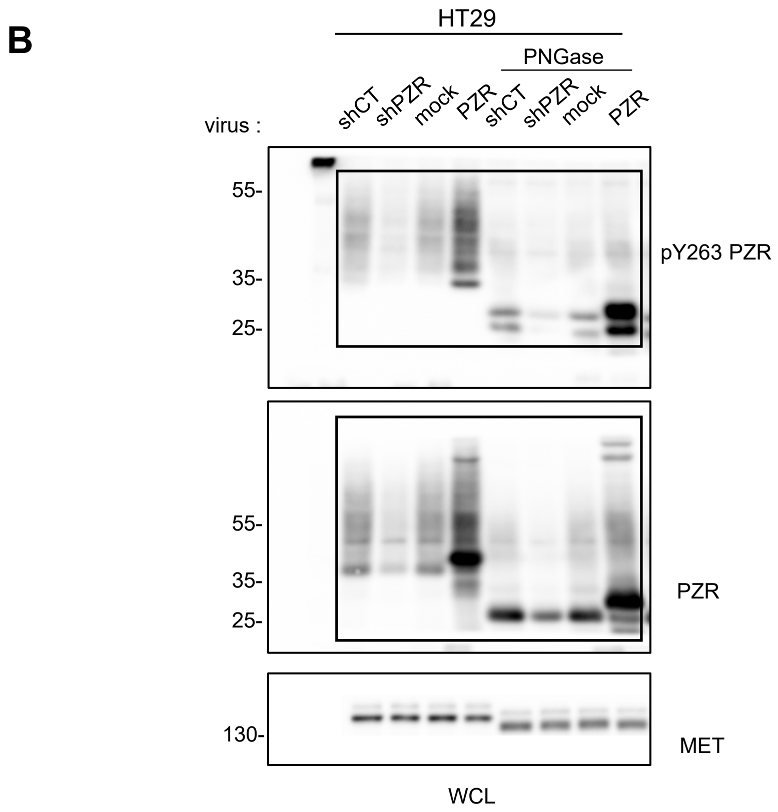

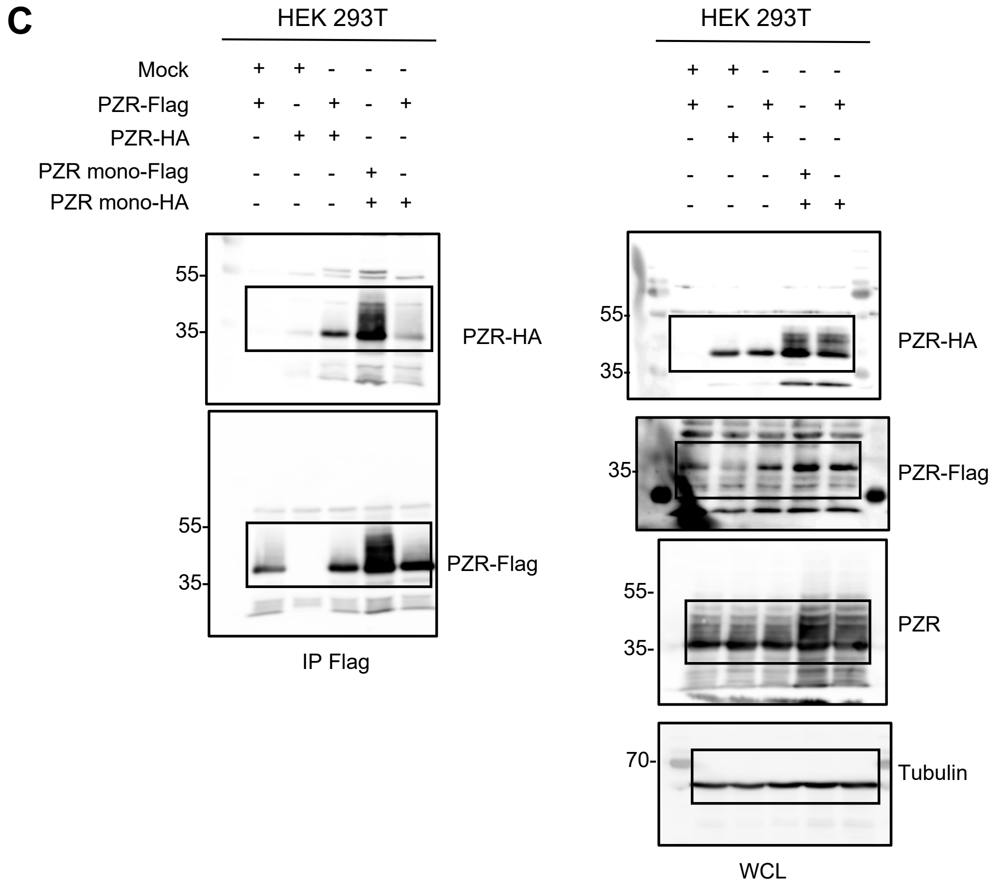

Figure S4A and B

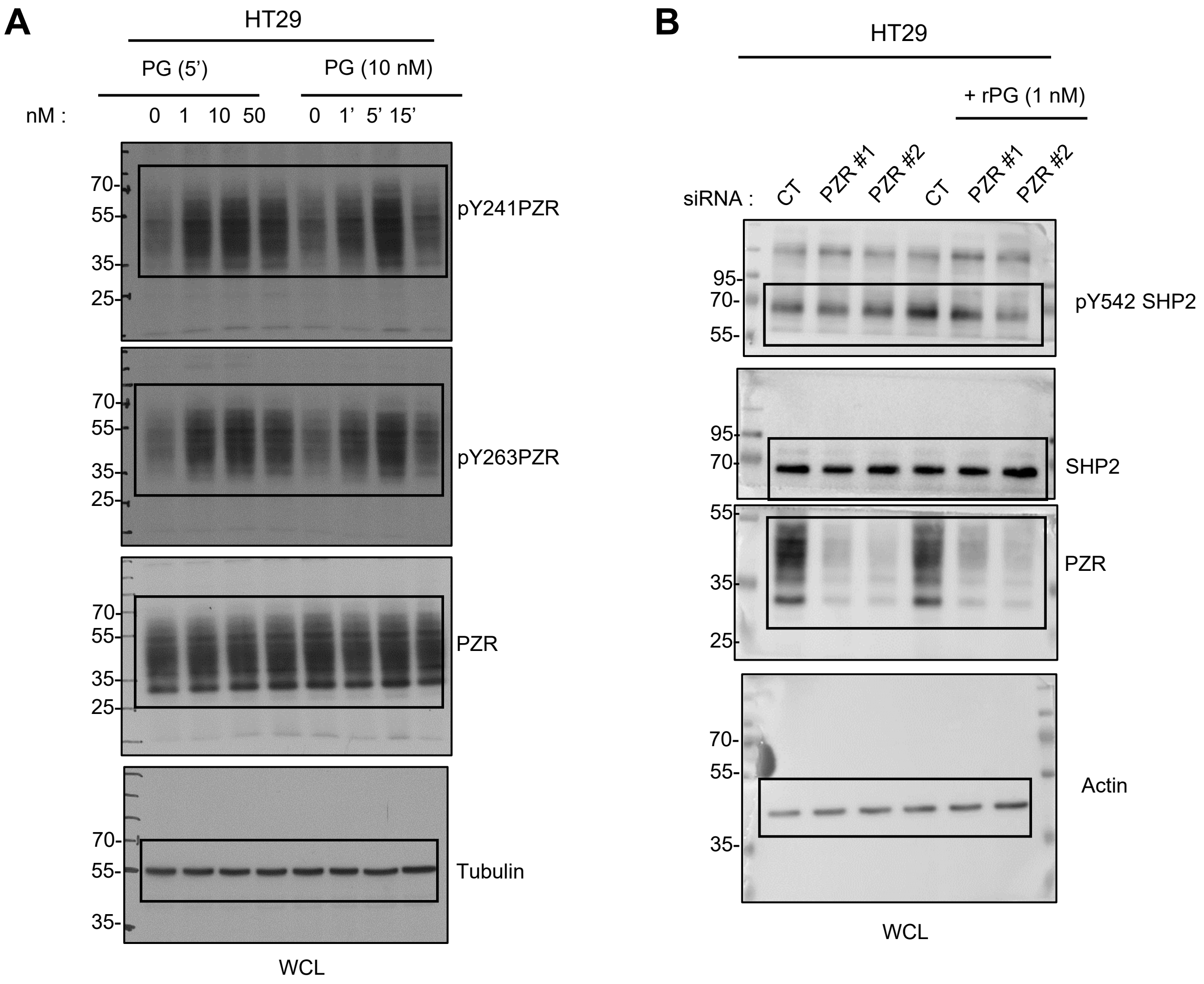

Figure S9C

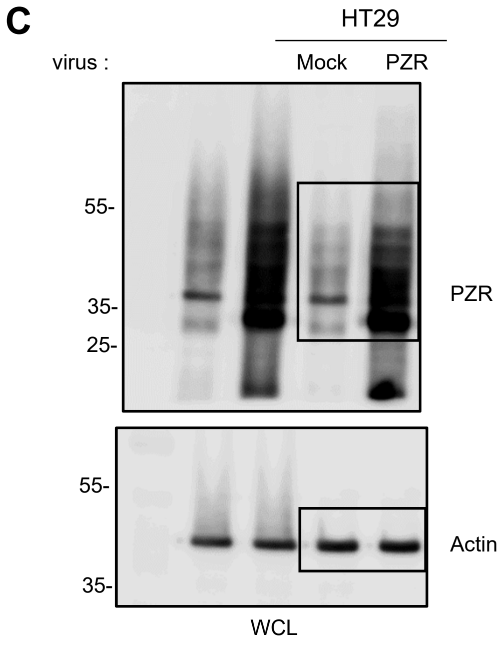

Figure S10A and B

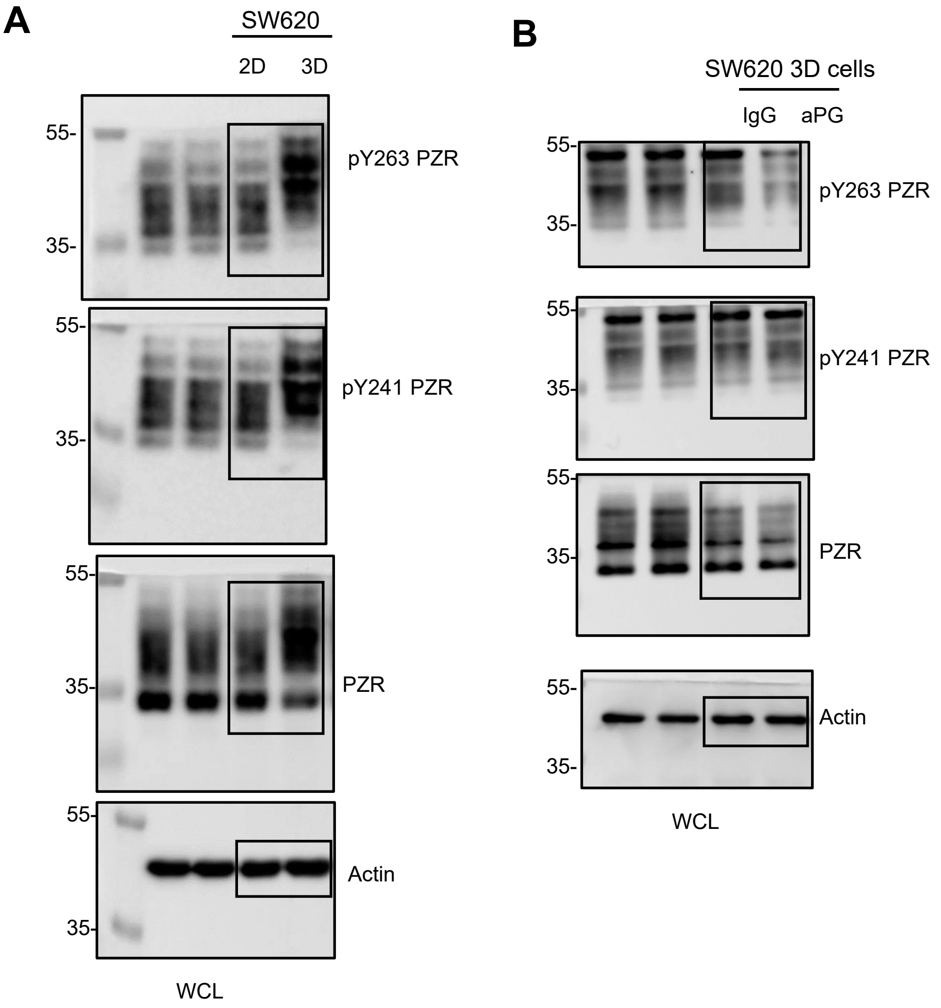

Figure S13A, E and F

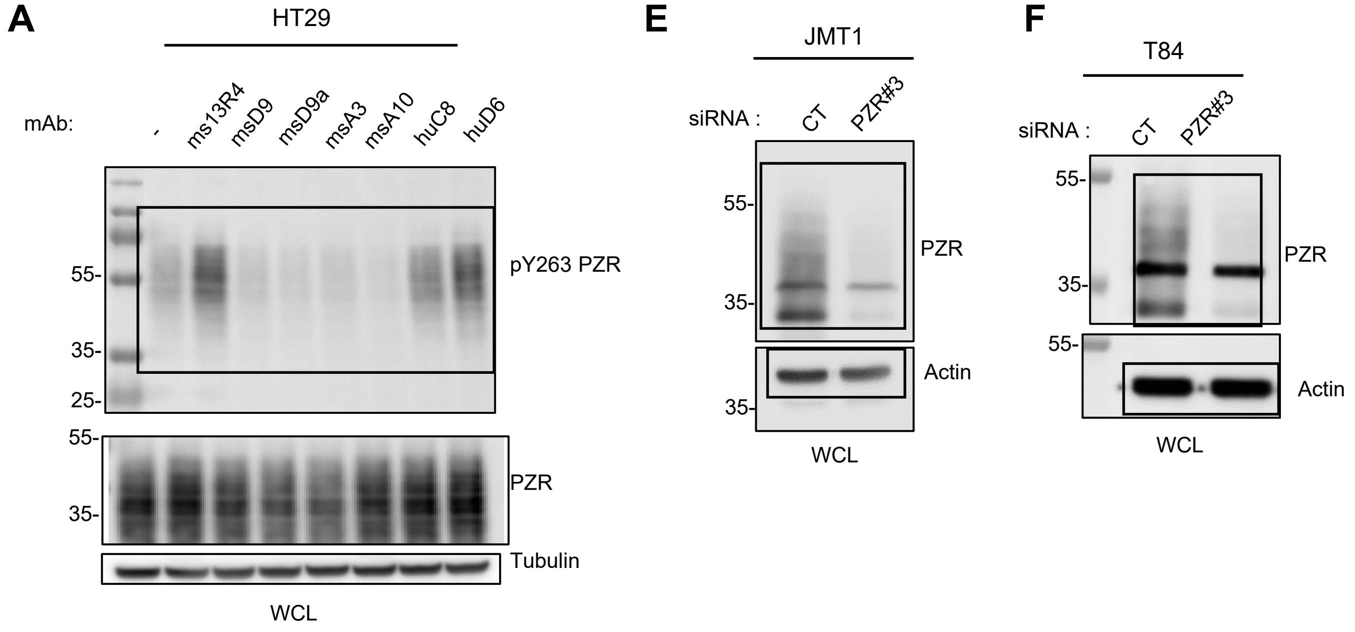

Table S1. List of primers for mutagenesis

Mutation	Forward primer	Reverse primer
PZR S86G	GGGGGCCGACACTACTGTGGGGTTTTTCCACTACTCCCAAGGG	CCCTTGGGAGTAGTGGAAAAACCCCACAGTAGTGTCGGCCCCC
PZR V145G/Q146K/P147T/G148S	CCCTCCTGACATCGTTGGCAAGACTTCACACATTAGGCTCTATGTCG	CGACATAGAGCCTAATGTGTGAAGTCTTGCCAACGATGTCAGGAGGG
PZR N50Q	GCCAAAAGAAATCTTCGTGGCACAGGGTACACAAGGGAAGC	GCTTCCCTTGTGTACCCTGTGCCACGAAGATTTCTTTTGGC
PZR N130Q	CATAGAAAATATGCAGTTTATACACCAGGGCACCTATATCTGTGATGTCAAA	TTTGACATCACAGATATAGGTGCCCTGGTGTATAAACTGCATATTTTCTATG
PZR Y241F	CACCAGGGCCCAGTCATATTTGCACAGTTAGACC	GGTCTAACTGTGCAAATATGACTGGGCCCTGGTG
PZR Y263F	CAAGTCAGAGTCTGTGGTGTTTGCGGATATCCG	CGGATATCCGCAAACACCACAGACTCTGACTTG

Table S2. List of siRNA sequences

siRNA	Target Sequence (5'-3')	
Human siCTRL	UUCUCCGAACGUGUCACGUUU	Eurofins Genomics
Human GAS siRNA	GAAGAAGCCUAUGGAUGGA	Eurofins Genomics
ON TARGET plus Human MPZL1 (9019) siRNA #1	AGUCAUAUAUGCACAGUUA	Dharmacon
ON TARGET plus Human MPZL1 (9019) siRNA #2	GAACUAAGGUUGCCUAAAG	Dharmacon
ON TARGET plus Human MPZL1 (9019) siRNA #3	GAGAAUACCUAGAACAUAU	Dharmacon

Table S3. List of primary antibodies used for IHC, fluorescence and PLA

IHC and fluorescence		
Antibody	Reference	Dilution
Rabbit pPZR Y263	Affinity #AY-AF7166	1/100
Rabbit pSHP2 Y542	Cell Signaling #3751S	1/100
Rabbit pSRC Y416	Cell Signaling #2101S	1/100
Rabbit Nuclear β-catenin	Cell Signaling #8814S	1/500
Rabbit Jagged1 (D4Y1R)	Cell Signaling #70109S	1/200
Rabbit GFP	Invitrogen #A6455	1/400
Rabbit Ki-67	Abcam #ab16667	1/200
Rabbit Cleaved caspase-3	Cell Signaling #9661S	1/400
Rat β-galactosidase	CNIO #3A9A10F8	1/200
Rabbit CD31	Abcam #ab28364	1/50
Mouse VEGF	Thermofisher #MA5-13182	1/20
Mouse BrdU (MoBU-1)	Thermofisher #35128	1/200
Mouse β-catenin	Santa Cruz #sc-7963	1/250
Rat CD45	Biolegend #103101	1/400

PLA			
PLA Binding	Mouse Anti Flag	Sigma-Aldrich #F3165	1/500
	Rabbit anti-PG N (1,7 mg/ml)		1/100
PLA Dimerisation	Rabbit anti-Flag	Sigma-Aldrich #F7425	1/250
	Mouse anti-myc	Cell Signaling #9B11	1/500
PLA D9a	Mouse Isotype control antibody 15,1 mg/ml (1/344)	Leinco Technologies #I-118	1/350

	Mouse D9a 4,4 mg/ml (1/100)	Evitria	1/100
	Rabbit anti-Flag	Sigma-Aldrich #F7425	1/250

Table S4. List of primers

human primers		
Gene name	Forward primer	Reverse primer
Nrarp	TCAACGTGAACTCGTTCGGG	ACTTCGCCTTGGTGATGAGAT
Hes1	GCACAGAAAGTCATCAAAGCCTAT	AATGCCGCGAGCTATCTTTCT
Jag1	CGGGATTTGGTTAATGGTTATC	ATAGTCACTGGCACGGTTGTAGCAC
Gast	CCACACCTCGTGGCAGAC	TCCATCCATCCATAGGCTTC
Gapdh	TCACCAGGGCTGCTTTTAAC	ATCTCGCTCCTGGAAGATGG
β-Actin	GGACTTCGAGCAAGAGATG	TAGCACTGTGTTGGCGTACAG
Mouse primers		
Mpzl1	GCTGACTTGCACATTCGATTC	ACCTGTCCTTGTGAGTAGTGG
Lgr5	GACAATGCTCTCACAGAC	GGAGTGGATTCTATTATTATGG
Ascl2	AAGCACACCTTGACTGGTACG	AAGTGGACGTTTGCACCTTCA
CD166	ATGGCATCTAAGGTGTCCCCT	CTGAGTTGACAGTGTACCATCC
CD133	GTGGAAGGAGCCCAGCTTAG	AGTACCATCCCTCTCCGGTC
CD44	CCACAGCCTCCTTTCAATAACC	GGAGTCTTCGCTTGGGGTA
Aldh1a1	ATACTTGTCGGATTTAGGAGGCT	GGGCCTATCTTCCAAATGAACA
Olfm4	GCAGCTGGAAGTATCCTCTGG	CTACAGCTTCCAAGGGCCAAT
Axin2	TGACTCTCCTTCCAGATCCCA	TGCCCACACTAGGCTGACA
Smoc2	GGAGCAGGGAAAGCAGATGAT	AACTTGCTCGGTCCAGAGTG
Msi	AAGATCCAGGGGTTTCGGC	CGATTGCGCCAGCACTTTAT

β-catenin	GAATGAAGGCGTGGCAACAT	CGACTGAAAGCCGCTTCTTG
Tcf7	CGCGGGATAACTACGGAAAGA	CATTTCTTTTTCCTCCTGTGGTGG
c-Myc	GCCCGCGATCAGCTCTCCTGA	CGTGGCTGTCTGCGGGGTTT
CyclinD1	AAGTGTGACCCGGACTGCCTCC	TGGCCTTGGGGTCGACGTTC
c-jun	GGGTGGGAGGGGTTACAAAC	GCCCTCCCTGCTTTGTGTTA
Fra1	ATGTACCGAGACTACGGGGAA	CTGCTGCTGTCGATGCTTG
Birc5	GAGGCTGGCTTCATCCACTG	ATGCTCCTCTATCGGGTTGTC
Gapdh	GGAGCGAGACCCCACTAACA	ACATACTCAGCACCGGCCTC
Hprt	GCAGTACAGCCCCAAAATGG	GGTCCTTTTCACCAGCAAGCT
Mrpl32	AGGTGCTGGGAGCTGCTACA	AAAGCGACTCCAGCTTTGCT

Table S5. List of genotyping primer sequences

Primer	Sequence	Amplicon size
Mpzl1-wtF	5' CCTCCTGGCGATTCTGAGTTGC 3'	205 pb
Mpzl1-wtR	5' ATAGAGCCTTAAGAATTGATCTGAGC 3'	
LacZ-417-F	5' GCTAATCACGACGCGCTGTATCGCTGG 3'	400 pb
LacZ-418-R	5' TTCATGCAGAACTGGCGATCGTTCGGCG 3'	
ApcLoxP/LoxP F	5' CTGTTCTGCAGTATGTTATCA 3'	250 pb: Flox
ApcLoxP/LoxP R	5' CTATGAGTCAACACAGGATTA 3'	180 pb: WT
Lgr5-CreERT2-IRES-eGFP F	5' GCAGAAGAACGGCATCAAG 3'	138 pb
Lgr5-CreERT2-IRES-eGFP R	5' GCTCAGGTAGTGGTTGTCG3'	
Villin-CreERT2 F	5' CAAGCCTGGCTCGACGGCC 3'	280 pb
Villin-CreERT2 R	5' CGCGAACATCTTCAGGTTCT 3'	
